# Identification of novel diagnostic panel for mild cognitive impairment and Alzheimer’s disease: findings based on urine proteomics and machine learning

**DOI:** 10.1186/s13195-023-01324-4

**Published:** 2023-11-04

**Authors:** Yuye Wang, Yu Sun, Yu Wang, Shuhong Jia, Yanan Qiao, Zhi Zhou, Wen Shao, Xiangfei Zhang, Jing Guo, Bin Zhang, Xiaoqian Niu, Yi Wang, Dantao Peng

**Affiliations:** 1 China-Japan Friendship Hospital, Chinese Academy of Medical Sciences & Peking Union Medical College, Beijing, China; 2 Department of Neurology, China-Japan Friendship Hospital, Beijing, 100029 China; 3 Peking University China-Japan Friendship School of Clinical Medicine, Beijing, China; 4 State Key Laboratory of Proteomics, Beijing Proteome Research Center, National Center for Protein Sciences (Beijing), Beijing Institute of Lifeomics, Beijing, 102206 China

**Keywords:** Urine proteomics, Alzheimer’s disease, Mild cognitive impairment, Biomarker, Diagnostic model, Machine learning

## Abstract

**Background:**

Alzheimer’s disease is a prevalent disease with a heavy global burden. Proteomics is the systematic study of proteins and peptides to provide comprehensive descriptions. Aiming to obtain a more accurate and convenient clinical diagnosis, researchers are working for better biomarkers. Urine is more convenient which could reflect the change of disease at an earlier stage. Thus, we conducted a cross-sectional study to investigate novel diagnostic panels.

**Methods:**

We firstly enrolled participants from China-Japan Friendship Hospital from April 2022 to November 2022, collected urine samples, and conducted an LC–MS/MS analysis. In parallel, clinical data were collected, and clinical examinations were performed. After statistical and bioinformatics analyses, significant risk factors and differential urinary proteins were determined. We attempt to investigate diagnostic panels based on machine learning including LASSO and SVM.

**Results:**

Fifty-seven AD patients, 43 MCI patients, and 62 CN subjects were enrolled. A total of 3366 proteins were identified, and 608 urine proteins were finally included in the analysis. There were 33 significantly differential proteins between the AD and CN groups and 15 significantly differential proteins between the MCI and CN groups. AD diagnostic panel included DDC, CTSC, EHD4, GSTA3, SLC44A4, GNS, GSTA1, ANXA4, PLD3, CTSH, HP, RPS3, CPVL, age, and APOE ε4 with an AUC of 0.9989 in the training test and 0.8824 in the test set while MCI diagnostic panel included TUBB, SUCLG2, PROCR, TCP1, ACE, FLOT2, EHD4, PROZ, C9, SERPINA3, age, and APOE ε4 with an AUC of 0.9985 in the training test and 0.8143 in the test set. Besides, diagnostic proteins were weakly correlated with cognitive functions.

**Conclusions:**

In conclusion, the procedure is convenient, non-invasive, and useful for diagnosis, which could assist physicians in differentiating AD and MCI from CN.

**Supplementary Information:**

The online version contains supplementary material available at 10.1186/s13195-023-01324-4.

## Background

Dementia is an international public health issue. In 2019, 57.4 million people were living with dementia globally. By 2050, the number of people is anticipated to increase to 152.8 million [1]. Alzheimer’s disease (AD) is the most common type of dementia, making up an estimated 60 to 80% of cases [2]. Estimates of the number of dementia and AD patients in China’s senior population aged 60 years and older were 15.07 and 9.83, respectively [3], indicating an unneglectable burden on China’s social and economic status. On the continuum of cognitive decline, mild cognitive impairment (MCI) is referred to as the symptomatic pre-dementia stage and is featured by an objective cognitive decline that is not serious enough to require assistance with daily activities. Early detection of MCI could suggest an elevated risk for AD, and early comprehensive interventions could stop or postpone the progression of MCI to dementia [4].

Based on core clinical criteria for AD dementia, the patients are classified into probable AD dementia and possible AD dementia in clinical practice [5]. Due to the lack of biomarkers, it is difficult to distinguish Alzheimer’s disease from other dementias [6]. Recently, both European and American associations highlighted the importance of biomarkers in AD which is featured by amyloid-β (Aβ) plaques (A), pathological tau (T), and neurodegeneration (N) [6–8]. A biomarker, aggregated Aβ or related pathologic state, could be evaluated by amyloid positron emission tomography (PET) or CSF Aβ_42_ or Aβ_42_/Aβ_40_ ratio [9]. T biomarker, aggregated tau (neurofibrillary tangles (NFTs)) or related pathologic state, could be reflected by tau PET or CSF phosphorylated tau. N biomarker, neurodegeneration or neuronal injury, could be evaluated by anatomic magnetic resonance imaging (MRI), fluorodeoxyglucose (FDG) PET, or CST total tau [7]. In the MCI stage, CSF-based biomarkers could also predict prognosis [10]. The most accurate way to quantify pathological accumulation in a live brain is using PET imaging, but its expense and complexity prevent it from becoming widely used [11]. Similarly, most patients are unwilling to undergo a lumbar puncture to get CSF since it is invasive. In other words, existing pathological biomarkers are difficult to popularize due to expense, radiation, complexity, and invasiveness which results in low patient acceptance. This emphasizes the need for less expensive and invasive methods.

Proteomics is the comprehensive study of the varied properties of proteins and peptides to fully describe the structure, function, and regulation of biological systems in both health and disease status [12]. Establishing human disease proteomics could contribute to clinical diagnosis and therapy [13]. The study and validation of biomarkers as well as the discovery and development of new medications might both benefit from proteomics [14]. As for applications in AD, unprecedented proteome coverage of bio-fluids, including cerebrospinal fluid and serum [15], yields new potential biomarkers for AD.

Urine is less intrusive, more accessible, and is not subject to homeostatic systems which accommodates several variations that might represent the body’s condition [16]. Besides, it has been suggested that urine was applied in neurodegenerative diseases [17]. In AD, secreted phosphoprotein 1 (SPP1), gelsolin (GSN), and insulin-like growth factor-binding protein 7 (IGFBP7) were suggested to differ in expression in the urine of AD patients and behave as potential biomarkers [18]. Moreover, Alzheimer-associated neuronal thread protein (AD7c-NTP) [19, 20] was often detected in urine in the early stage of AD and MCI which was also suggested to be a biomarker, as well as apolipoprotein C3 (ApoC3) [21] which was validated by enzyme-linked immunosorbent assay (ELISA). Considering these backgrounds, the use of urine proteomics in the AD area is promising.

In this study, we firstly enrolled AD patients, MCI patients, and cognitive normal (CN) subjects. Then, we collected urine samples, and the urine samples were undergone an LC–MS/MS test. We aim to conduct an analysis based on urine proteomics and machine learning to identify novel diagnostic panels for early diagnosis of MCI and AD.

## Methods

### Subject enrollment

This study was a cross-sectional study that enrolled participants from China-Japan Friendship Hospital from April 2022 to November 2022. A total of 162 participants, over 50 years old, including 57 AD patients, 43 MCI patients, and 62 CN subjects were included in the final analysis. Risk factors were collected, and APOE genotypes were classified into ε4 carriers and non-carriers. Sex, living status, education, smoking status, and family histories matched among the groups. Besides, the distribution of hypertension, diabetes, hyperlipidemia, heart diseases, and cerebrovascular diseases among the three groups did not reach statistical significance. Age, the most important risk factor of AD, was more senior in the AD group compared to the CN group. APOE ε4, the main genetic risk factor for sporadic AD, was more prevalent in the AD and MCI groups compared with the CN group. The overall information is summarized in Table 1.

**Table 1:** Basic information and risk factors of included participants

	AD (*n* = 57)	MCI (*n* = 43)	CN (*n* = 62)	*p*
Age (median, P25, P75)	79 (72.5, 82)	74 (68, 78)	70(63.75, 73.5)	**< 0.001**^**a**^
Gender (male/female)	27/30	14/29	22/40	0.253
Living alone (yes/no)	1/56	0/43	2/60	0.482
Education (median, P25, P75)	12 (9, 16)	12 (9, 15)	15 (11, 16)	0.054
Smoking (yes/no)	15/42	8/35	10/52	0.366
Hypertension (yes/no)	22/35	15/28	31/31	0.247
Diabetes (yes/no)	11/46	5/38	16/46	0.199
Hyperlipidemia (yes/no)	23/34	23/20	35/27	0.186
Heart diseases (yes/no)	16/41	10/33	10/52	0.288
Cerebrovascular diseases (yes/no)	14/43	8/35	11/51	0.618
Family history (yes/no)	10/47	7/36	13/49	0.808
APOE (ε4 carrier/non-carrier)	27/30	21/22	14/48	**0.005**^**ab**^

^a^Significant differences between AD and CN (Bonferroni-corrected *p* < 0.05)

^b^Significant differences between MCI and CN (Bonferroni-corrected *p* < 0.05)

All subjects underwent medical history collection, a battery of neuropsychological assessments and apolipoprotein E (APOE) genotype test. Most individuals underwent quantitative electroencephalography (qEEG) and magnetic resonance imaging (MRI). The study protocol was approved by the China-Japan Friendship Hospital ethics committee and institutions (Ethics ID: 2020–31-Y06-32). Consent forms were obtained from all participants.

### Inclusion and exclusion criteria

AD is clinically diagnosed using the 2011 National Institute on Aging-Alzheimer’s Association (NIA-AA) criteria [5]. The contents are as follows: (1) meet the core clinical criteria including interference with the ability to complete daily activities and a decline from previous levels, (2) characterized by insidious onset and clear-cut history of decline of cognition, and (3) excluding dementia due to other etiologies.

MCI is defined with the 2011 NIA-AA diagnostic criteria [22], as the following shows: (1) concern about a cognition decline compared with the previous status, reported by the patient himself, the informant, or a skilled physician; (2) decline in at least one cognitive domain after age and education adjustment; (3) maintenance of independent function in daily life activities; and (4) not meeting the diagnostic criteria for dementia.

CN controls were those who performed normally on the standardized neuropsychological tests and with or without cognitive complaints or concerns during the structured interview.

Briefly, MMSE cutoff points for dementia/non-dementia were 16/17 for illiterate, 19/20 for individuals with 1–6 years of education, and 23/24 for individuals with 7 or more years of education [23]. The ADL cutoff was 26. The definition of cognitive decline in domains was a decrease of more than 1.5 standard deviations in at least one test. Besides, medical history and imaging evidence were taken into consideration. In summary, patients were diagnosed according to the clinical criteria based on comprehensive assessments.

The exclusion criteria are as follows: (1) cognitive decline caused by severe psychiatric disorders or mental retardation; (2) cognitive impairment caused by other neurological diseases, such as trauma, stroke, tumor, parkinsonism, encephalitis or epilepsy, or other types of dementia, such as frontotemporal dementia (FTD), Lewy body dementia (LBD), and vascular dementia (VaD); (3) cognitive impairment caused by diseases of other systems such as severe anemia and thyroid disorders; (4) a history of urinary system disorders, malignant tumor, or other severe diseases; and (5) inability to cooperate in completing neuropsychological tests or incomplete clinical data.

### Neuropsychological scale assessment

The neuropsychological test battery included measures of global cognition and cognitive performance in the domains of memory, executive function, attention, language, and visuospatial ability. Participants were administered the Mini-Mental State Examination (MMSE) and Montreal Cognitive Assessment (MoCA) for global cognition. The Activity of Daily Living Scale (ADL) was used for accessing the function ability during daily life. The Rey Auditory Verbal Learning Test-immediate recall (RAVLT-I) and Rey Auditory Verbal Learning Test-delayed recall (RAVLT-D) were administered to assess memory; Digit Span Test (DST)-Backward and Stroop Color and Word Test (SCWT) were used for accessing executive function; DST-Forward and Symbol Digit Modalities Test (SDMT) were used for accessing attention; Boston Naming Test (BNT) and Verbal Fluency Test (VFT) were administered to assess language. In addition, the Clock Drawing Test (CDT) and Rey Complex Figure Test (RCFT) were utilized to assess visuospatial ability. The above scales have been applied in clinical practice and published in previous articles from our team [24].

### Urine sample preparation

A midstream of random urine was collected and stored at − 80 °C. A biosafety level II lab was used to prepare samples. The pellet from the urine was obtained after being centrifuged at 176,000* g* for 1 h and then was re-suspended using 40 μL of resuspension buffer containing 50 mmol L^−1^ Tris–HCl, 250 mmol L^−1^ sucrose, pH 8.5, and then reduced with 50 mmol L^−1^ dithiotheitol (DTT) at 65 °C for 30 min. After adding 160 μL wash buffer (10 mmol L^−1^ Tris–HCl, pH 7.4, 100 mmol L^−1^ NaCl), a second ultracentrifugation at 176,000* g* was performed for 30 min. The pellet was re-suspended with 30 μL 50 mM NH_4_HCO_3_, heated for 3 min at 95 °C, cooled to room temperature, and then digested by trypsin at a protease-to-protein ratio of 1:100 (w/w), incubating overnight at 37 °C.

### LC–MS/MS analysis

The digested peptides were vacuum-dried in a SpeedVac. Then, samples were stored at − 80 °C until further use. Peptide samples were re-dissolved in 0.1% formic acid (FA)-H_2_O. One-microgram peptide samples were loaded onto a trap column (100 μm × 2 cm, homemade; particle size, 3 μm; pore size, 120 Å; SunChrom, USA). Solvent A was 0.1% FA in H_2_O, and solvent B was 0.08% FA and 20% H_2_O in Acetonitrile (ACN). Peptides were separated by a homemade silica microcolumn (150 μm × 10 cm, particle size, 1.9 μm; pore size, 120 Å; SunChrom, USA) with a gradient of 5–35% solvent B at a flow rate of 800 nL/min for 30 min. Liquid chromatography coupled to tandem mass spectrometry (LC–MS/MS) was performed on a Q Exactive HF-X mass spectrometer (Thermo Fisher Scientific, USA). The instrument was run in the data-dependent acquisition (DDA) mode. The whole scan was processed in the Orbitrap from *m*/*z* 300–1400 at a resolution of 60,000 with an automatic gain control (AGC) target of 3e^6^ and a 20-ms maximum injection time. With a normalized collision energy of 27%, the top 40 most intense ions in each scan cycle were chosen for high-energy collision dissociation (HCD) fragmentation. For the MS/MS scan, the fragment ions were identified in the Orbitrap with a resolution of 7500, an AGC target of 5e^4^, a maximum injection time of 12 ms, and a dynamic exclusion of 15 s. Trypsin digests of 293 T cells were used to prepare quality control samples which were then routinely evaluated to determine the sensitivity and reproducibility of LC–MS/MS. The mass spectrometry proteomics data have been deposited to the ProteomeXchange Consortium (http://proteomecentral.proteomexchange.org) via the iProX partner repository [25, 26] with the dataset identifier PXD044672.

### Protein identification and label-free quantification (LFQ)

The Firmiana platform was used to process the mass spectrometry data [27]. The MASCOT search engine (Matrix Science, version 2.3.01) was used to identify proteins in the NCBI human RefSeq protein database (published on 04/07/2023, 33,118 entries). Precursor ion mass tolerance was set to 20 ppm, while product ion mass tolerance was set at 0.05 Da. Trypsin digestion may miss at most one cleavage. Dynamic modifications included methionine oxidation and N-terminal acetylation. For the following analyses, only ≥ 1 unique and strict peptide, ≥ 2 strict peptides (ion score > 20), or ≥ 3 strict peptides with protein levels equal to 1% FDR were employed. Protein quantification was carried out using the intensity-based absolute quantification (iBAQ) algorithm [28]. We converted the iBAQ to the fraction of total (FOT) to normalize the differences in sample amounts [29], which was calculated by the iBAQ value of each protein divided by the total iBAQ of the sample, multiplied by 10^5^. All missing values were replaced with zeros. Proteins detected in more than 50% of the samples were included for further analysis. A total of 608 proteins were retained, and the imputation of missing values was based on the *k*-nearest neighbor (KNN) method using the “Wu Kong” platform (https://www.omicsolution.org/wkomics/main/).

### Statistical analysis and bioinformatics analysis

SPSS 23.0 was used for statistical analysis. The Shapiro–Wilk test was used to examine the normality of quantitative data. The mean (*x* ± *s*) was used for the description of normal data while non-normal data used median (P25, P75). Analysis of variance (ANOVA) was used for normal data mean comparison while the Kruskal–Wallis *H* test was utilized for non-normal data distribution comparison. For post hoc comparisons, *p*-values were Bonferroni-corrected. Besides, Pearson’s chi-square test or Fisher’s exact probability was used for the comparison of the proportions of categorical variables. Statistical significance was defined as a two-tailed *p*-value < 0.05. To construct a protein–protein interaction (PPI) network, we used the stringApp in *cytoscape*, and BiNGo in *cytoscape* was used for Gene Ontology (GO) enrichment with Benjamini–Hochberg corrected *p*-value < 0.05. In parallel, R (4.1.0) was used for bioinformatics analysis. Differential urinary proteins were filtered utilizing *limma* package [30] with a threshold of *p* < 0.05 and the absolute value of log2 fold change (log2FC) > 0.58 after log_2_ transformation and normalization. Heatmap was presented using *pHeatmap* [31], and the volcano plot was presented using *EnhancedVolcano* [32]. The expression levels of selected proteins were shown in the boxplot by *ggpubr* [33] package. Gene set enrichment analysis (GSEA) was used to investigate various GO terms and Kyoto Encyclopedia of Genes and Genomes (KEGG) pathways that might be related with AD or MCI when compared to CN in all proteins. *clusterProfiler* package [34, 35] was utilized for enrichment analysis while *enrichplot* package [36] was utilized for visualization. Moreover, the *corrplot* [37] package was used for the visualization of the correlation relationship.

### Machine learning

In order to distinguish AD from CN and MCI from CN, machine learning was utilized to determine the best multivariate signatures, which included both proteins and demographic information (age and APOE 4 status) as input parameters. The classifier consisted of feature selection and classifiers [38]. Briefly, the dataset was separated into a training set (0.7) and a test set (0.3). The least absolute shrinkage and selection operator (LASSO) was utilized to select the “n” top input variables that best differentiated AD or MCI diagnostic groups with minimum mean square error (MSE). On top of these “n” characteristics, support vector machine (SVM) classifiers were built to forecast the result under tenfold cross-validation. Linear, polynomial, radial, and sigmoid kernel functions were compared. Accuracy and area under the curve (AUC) (receiver operating characteristic (ROC) curve) were used for the diagnostic value evaluation when testing the model in the test set.

## Results

### Clinical characteristics of enrolled participants

Table 2 presented the cognitive assessment results, percentage of abnormal qEEG, and medial temporal lobe atrophy (MTA) scales of each group. As for neuropsychological assessments, the results showed that there were significant differences among the three groups using the Kruskal–Wallis *H* test (*p* < 0.001). For post hoc comparisons, there were differences between the AD and CN groups as well as the MCI and CN groups in global cognition as indicated by MMSE and MoCA, memory domain as indicated by RAVLT-I and RAVLT-D, executive function as indicated by DST-Backward and SCWT, attention domain when indicated by SDMT, language as indicated by VFT and BNT, and visuospatial processing as indicated by CDT and RCFT. There were only differences between the AD and CN groups in ADL and DST-Forward. The individual basic information and results of neuropsychological tests for each participant were uploaded as Additional file 6: Table S1. Besides, the percentage of abnormal qEEG was higher in the AD group than in the CN group (*p* < 0.05). In parallel, there were differences between AD and CN in MTA scales (*p* < 0.001) in which the left-sided hippocampus atrophy of patients was more severe.

**Table 2:** Neuropsychological assessment and other clinical indicators of included participants

	AD	MCI	CN	*p*
Global cognition				
MMSE	15 (9.5, 19)	24 (22, 26)	27 (26, 28)	**< 0.001**^**ab**^
MoCA	7 (4, 12)	18 (13.75, 21)	23 (21, 26)	**< 0.001**^**ab**^
ADL	36.5 (29, 49.75)	20 (20, 23)	20 (20, 20)	**< 0.001**^**a**^
Memory				
RAVLT-I	8.5 (2, 16.25)	20.5 (14.75, 24.25)	32.5 (26.75, 41.5)	**< 0.001**^**ab**^
RAVLT-D	0 (0, 0)	0 (0, 0)	6 (4, 8)	**< 0.001**^**ab**^
Executive function				
DST-Backward	3 (1.5, 3)	3 (3, 4)	4 (3, 4)	**< 0.001**^**ab**^
SCWT	24 (7, 41)	46 (38, 49)	50 (46.25, 50)	**< 0.001**^**ab**^
Attention				
DST-Forward	7 (6, 8)	8 (6.75, 9)	8.5 (8, 9)	**< 0.001**^**a**^
SDMT	0 (0, 7.75)	20 (13.5, 29)	28 (20, 39.25)	**< 0.001**^**ab**^
Language				
BNT	10 (7, 16.25)	19 (15, 23)	26 (23, 27.25)	**< 0.001**^**ab**^
VFT	15 (8.75, 21)	26 (23, 34)	40 (34, 49)	**< 0.001**^**ab**^
Visuospatial ability				
CDT	5 (1, 17)	22 (15, 28)	26 (24, 30)	**< 0.001**^**ab**^
RCFT	0 (0, 0)	2 (0, 8.5)	11 (5, 17)	**< 0.001**^**ab**^
qEEG (abnormal%)	64.6	51.5	35.9	**0.041**^**a**^
MTA-Left	3 (2, 3)	1.5 (1, 2)	0 (0, 1)	**< 0.001**^**a**^
MTA-Right	2 (2, 3)	1 (1, 1.75)	0 (0, 1)	**< 0.001**^**a**^

*MMSE* Mini-Mental Status Examination, *MoCA* Montreal Cognitive Assessment, *ADL* Activity of Daily Living Scale, *RAVLT-I* Rey Auditory Verbal Learning Test-Immediate, *RAVLT-D* Rey Auditory Verbal Learning Test-Delay, *DST* Digit Span Test, *SCWT* Stroop Color and Word Test, *TMT* Trail Making Test, *SDMT* Symbol Digit Modalities Test, *BNT* Boston Naming Test, *VFT* Verbal Fluency Test, *CDT* Clock Drawing Test, *RCFT* Rey Complex Figure Test, *qEEG* quantitative electroencephalography, *MTA* Medial Temporal Lobe Atrophy Scale

^a^Significant differences between AD and CN (Bonferroni-corrected *p* < 0.05)

^b^Significant differences between MCI and CN (Bonferroni-corrected *p* < 0.05)

### Identified proteins and differential urinary proteins

The proteomics analysis performed was a LFQ quantitative analysis in DDA mode. In total, 3366 proteins were identified. Only the protein that could be detected in the majority (more than 50%) of the samples was included, and at last, a total of 608 proteins were included for further analysis (Additional file 7: Table S2). After imputing missing values using the KNN method, a complete expression matrix was constructed. GSEA results of all proteins were shown in Additional file 1: Fig. S1. In AD samples, a number of biological pathways and processes related to the immune system were enriched, whereas in MCI samples, a number of biological pathways and processes related to metabolism were enriched.

**Fig. 1:**
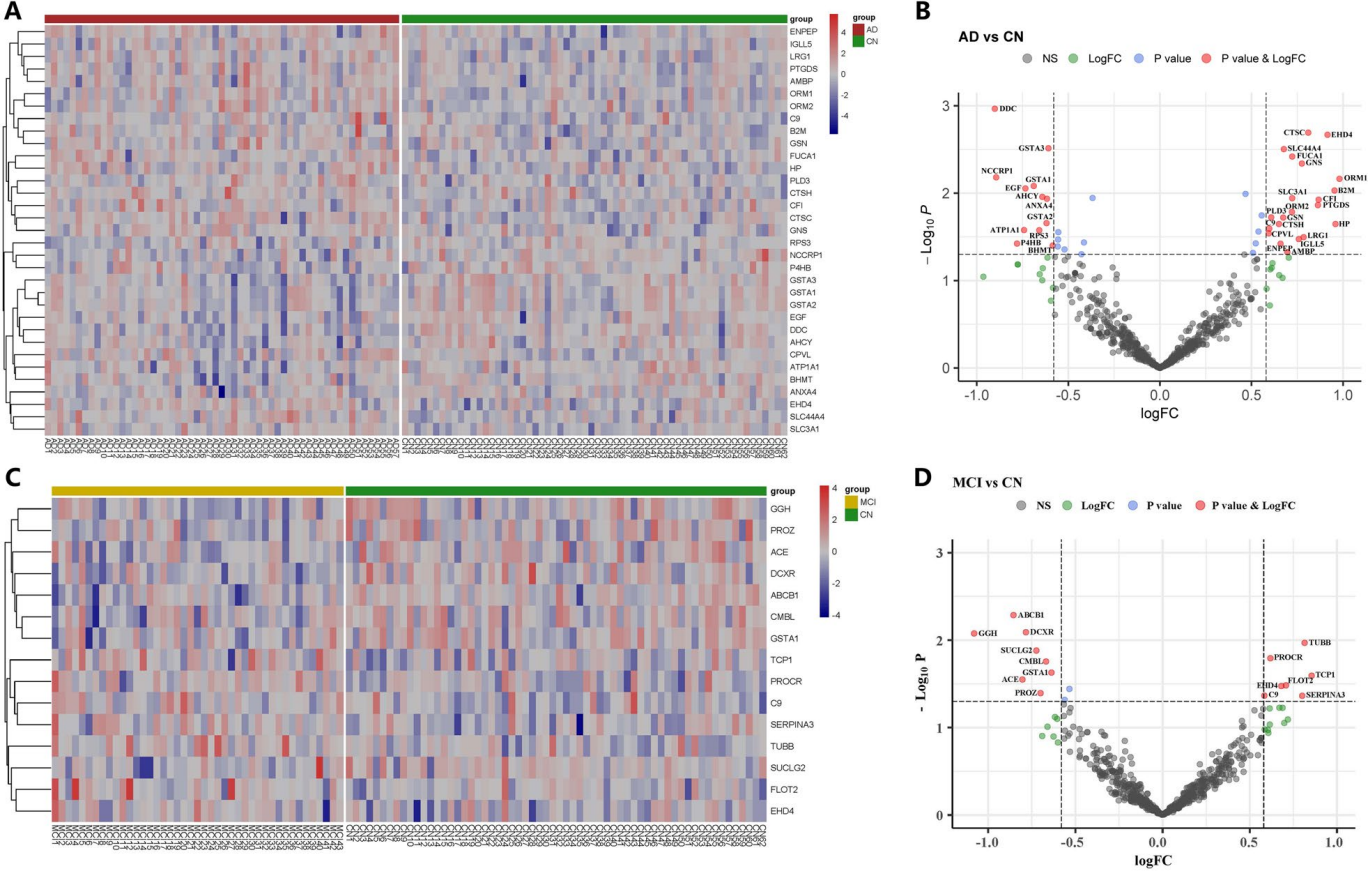
Differential urinary proteins in the AD and MCI groups compared to CN. **A** Heatmap of a total of 33 differential proteins between AD and CN. **B** Volcano plot showed the distribution of all proteins between AD and CN. The red dots were coincident with the left heatmap. **C** Heatmap of a total of 15 differential proteins between MCI and CN. **D** Volcano plot shows the distribution of all proteins between AD and CN. The red dots were coincident with the left heatmap. The horizontal dashed line indicates the threshold of *p*-value (− log_10_0.05 ≈ 1.3). The vertical dashed line indicates the threshold of fold change (± log_2_1.5 ≈ ± 0.58)

The protein expression levels of the samples were log2 transformed and normalized. Differential urinary proteins were filtered with a threshold of *p* < 0.05 and the absolute value of log_2_ fold change (log_2_FC) > 0.58. Compared to the CN group, significantly differential proteins were filtered in the AD group and MCI group by setting the threshold above. A table with the log_2_FC, *p*-values, and Benjamini–Hochberg corrected *p*-values of the 608 proteins included in the analysis was uploaded as Additional file 8: Table S3. The expression of the differential proteins in the AD group was displayed as a heatmap and a volcano plot (Fig. 1A, B) while the expression of the differential proteins in the MCI group was shown in Fig. 1C, D. There were 33 significantly differential proteins between the AD and CN groups among the 608 proteins included in the analysis, including 21 upregulated ones and 12 downregulated ones. In parallel, there were 15 significantly differential proteins between the MCI and CN groups among the 608 proteins included in the analysis, including 7 upregulated ones and 8 downregulated ones. These differential proteins were respectively inputted in LASSO for diagnostic panel selection. GSTA1 was downregulated in both AD and MCI while EHD4 and C9 were both upregulated in AD and MCI urine samples. The differential proteins between the AD and MCI groups were shown in Additional file 2: Fig. S2. A Venn diagram showing the intersection between the groups was shown in Additional file 3: Fig. S3.

### Protein–protein interaction network construction

**Fig. 2:**
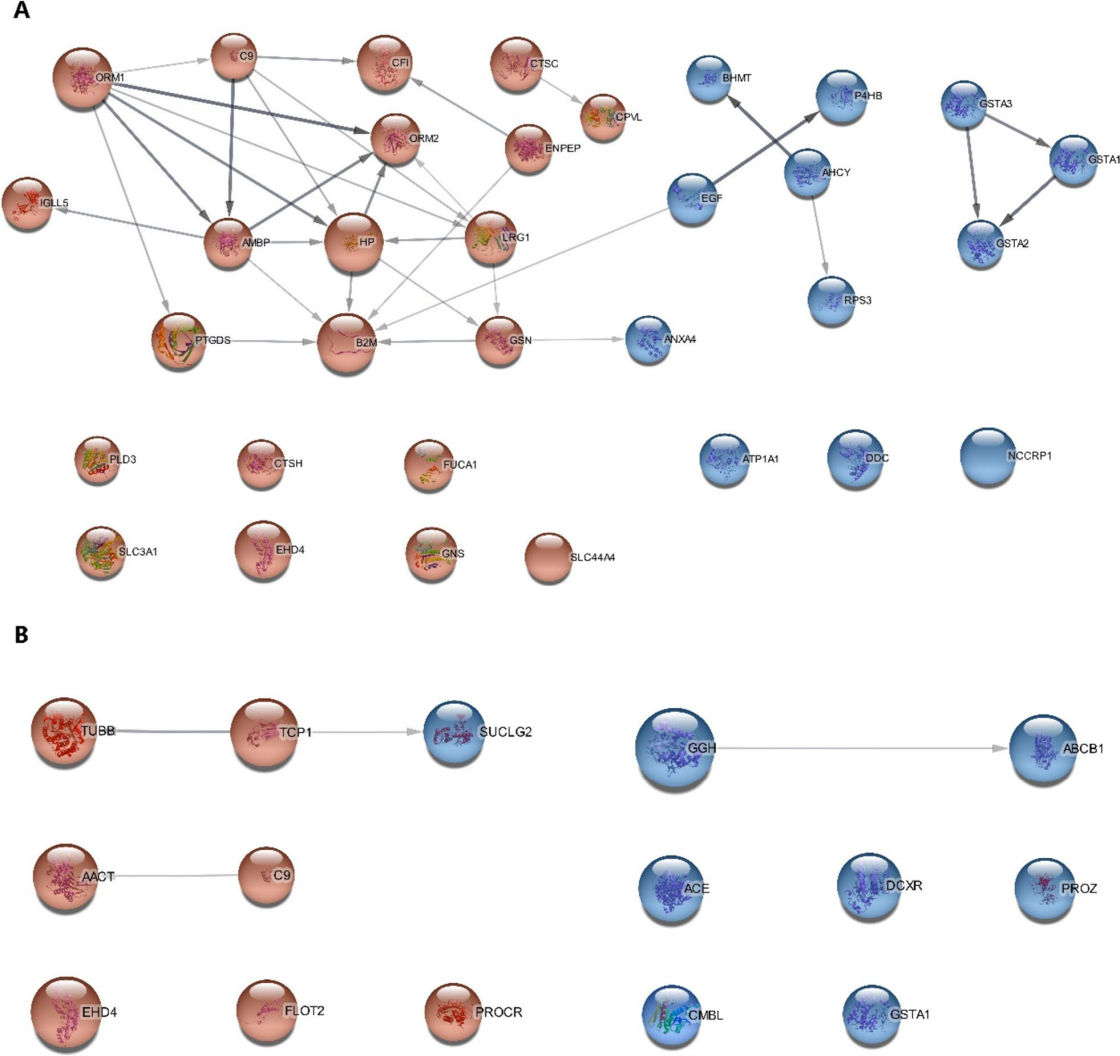
Protein–protein interaction network of significantly differential proteins. **A** Network of AD-CN differential proteins. **B** Network of MCI-CN differential proteins. The size of the node indicated relative fold change of differential proteins when compared to the controls. Red indicated upregulation, and blue indicated downregulation

With the help of stringApp in *cytoscape*, differential proteins were inputted, and the PPI network was constructed (Fig. 2). While proteins with an unknown 3D structure were represented by empty nodes, those with a known or predicted 3D structure were represented by filled nodes. The red nodes indicated upregulated proteins, and the blue nodes indicated downregulated proteins. The size reflected relative fold change when compared to CN. Besides, 33 biological processes in the AD-CN group and 67 biological processes in the MCI-CN group mainly related to the immune system and metabolism were enriched (Benjamini–Hochberg corrected *p*-value < 0.05). The enrichment networks are shown in Additional file 4: Fig. S4, and relative details are shown in Additional file 9: Table S4.

### Identification of a novel diagnostic panel based on the LASSO model

**Fig. 3:**
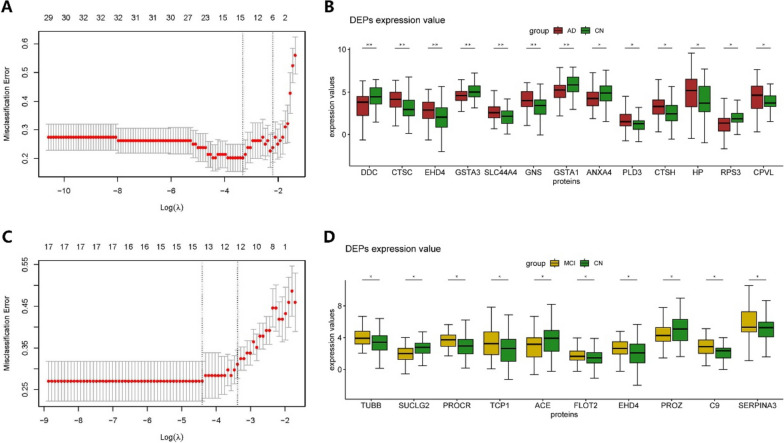
Diagnostic panel constructed by LASSO model. **A** LASSO model for variable selection in the AD-CN group. **B** Boxplot of the included diagnostic proteins for AD diagnosis. **C** LASSO model for variable selection in the MCI-CN group. **D** Boxplot of the included diagnostic proteins for MCI diagnosis. *p < 0.05; **p < 0.01. DDC, dopa decarboxylase; CTSC, cathepsin C; EHD4, EH domain containing 4; GSTA3, glutathione *S*-transferase alpha 3; SLC44A4, solute carrier family 44 member 4; GNS, glucosamine (*N*-acetyl)-6-sulfatase; GSTA1, glutathione *S*-transferase alpha 1; ANXA4, annexin A4; PLD3, phospholipase D family member 3; CTSH, cathepsin H; HP, haptoglobin; RPS3, ribosomal protein S3; CPVL, carboxypeptidase vitellogenic like; TUBB, tubulin beta class I; SUCLG2, succinate-CoA ligase GDP-forming subunit beta; PROCR, protein C receptor; TCP1, T-complex 1; ACE, angiotensin I-converting enzyme; FLOT2, flotillin 2; PROZ, protein Z, vitamin K-dependent plasma glycoprotein; C9, complement C9; SERPINA3, serpin family A member 3

Based on previous analysis, we extracted all differential proteins (33 in the AD-CN group and 15 in the MCI-CN group) plus age and APOE ε4 status to construct the LASSO model. For the AD-CN model, 13 proteins, age, and APOE ε4 status were identified when MSE reached minimum with the value of lambda (min) equaling 0.03225 (Fig. 3A). DDC, CTSC, EHD4, GSTA3, SLC44A4, GNS, GSTA1, ANXA4, PLD3, CTSH, HP, RPS3, CPVL, age, and APOE ε4 status were included in AD diagnostic panel. The boxplots showed the expression value of these proteins (Fig. 3B). Similarly, for the MCI-CN model, 10 proteins, age, and APOE ε4 status were identified when MSE reached minimum with the value of lambda (min) equaling 0.0191 (Fig. 3C). TUBB, SUCLG2, PROCR, TCP1, ACE, FLOT2, EHD4, PROZ, C9, SERPINA3, age, and APOE ε4 status were included in the MCI diagnostic panel. The boxplots showed the expression value of these proteins (Fig. 3D). EHD4 was considered valuable for both AD and MCI diagnosis.

### Evaluation of diagnostic value based on the SVM model

**Fig. 4:**
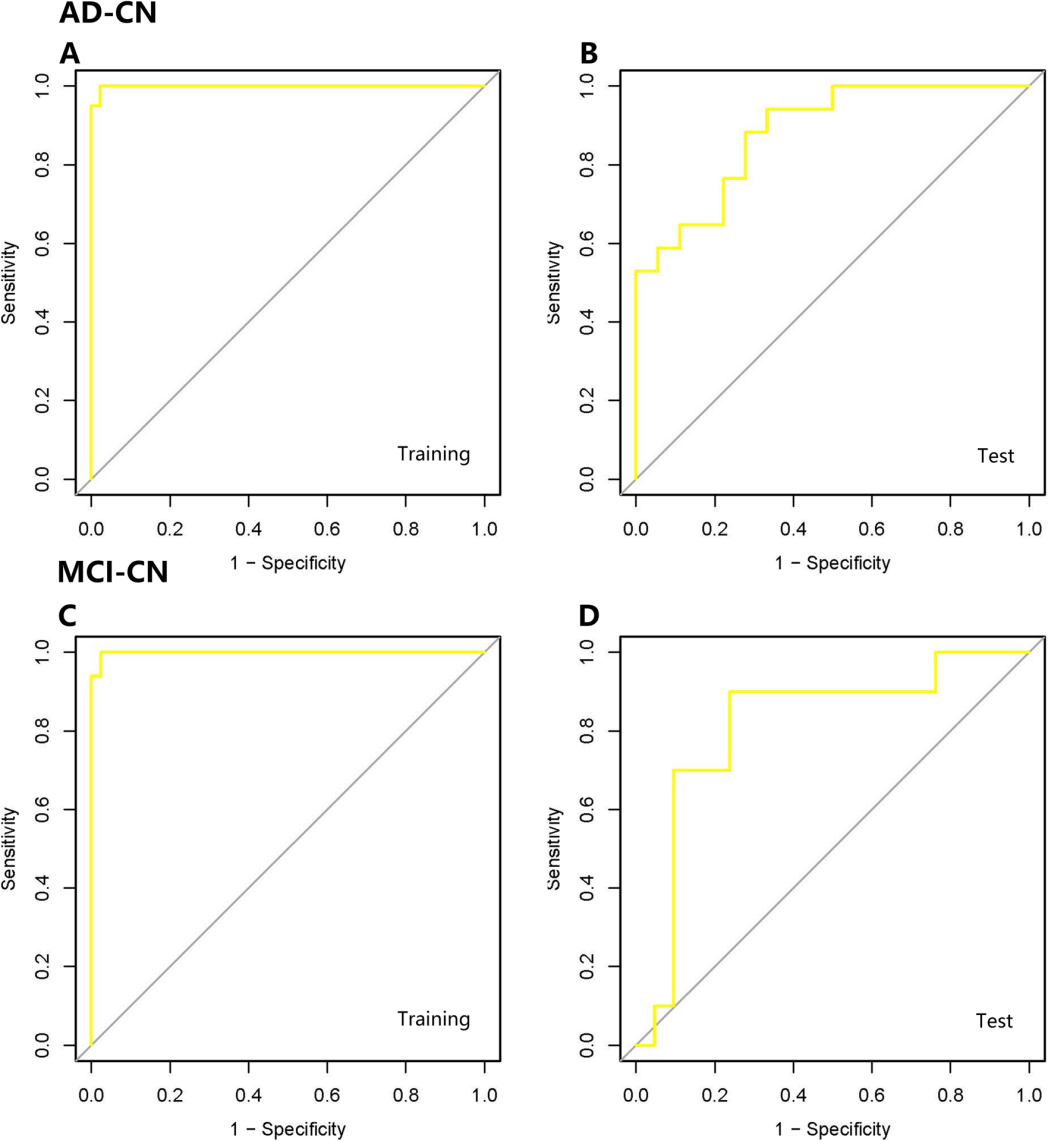
ROC curve for AD and MCI diagnosis in different SVM models. **A** ROC curve for AD diagnosis in the training set. **B** ROC curve for AD diagnosis in the test set. **C** ROC curve for MCI diagnosis in the training set. **D** ROC curve for MCI diagnosis in the test set

Based on LASSO results, we built SVM classifiers with tenfold cross-validation to investigate the ideal multivariate signatures that distinguished AD or MCI from CN. After training in training sets, we compared the relative indicators using different kernel functions in SVM. Radial achieved the highest predictive value with an accuracy of 0.9881, an F1 measure of 0.9876, and an AUC of 0.9739 in the AD-CN group and an accuracy of 0.973, an F1 measure of 0.9688, and an AUC of 0.9985 in the MCI-CN group in the training set. The model achieved a high predictive value with an accuracy of 0.7714, an F1 measure of 0.6923, and an AUC of 0.8824 in the AD-CN group and an accuracy of 0.8387, an F1 measure of 0.7386, and an AUC of 0.8143 in the MCI-CN group in the test set. Figure 4 shows the ROC curve in the training sets and test sets either in the AD-CN group (Fig. 4A, B) or in the MCI-CN group (Fig. 4C, D).

### Diagnostic proteins were correlated with cognitive functions

**Fig. 5:**
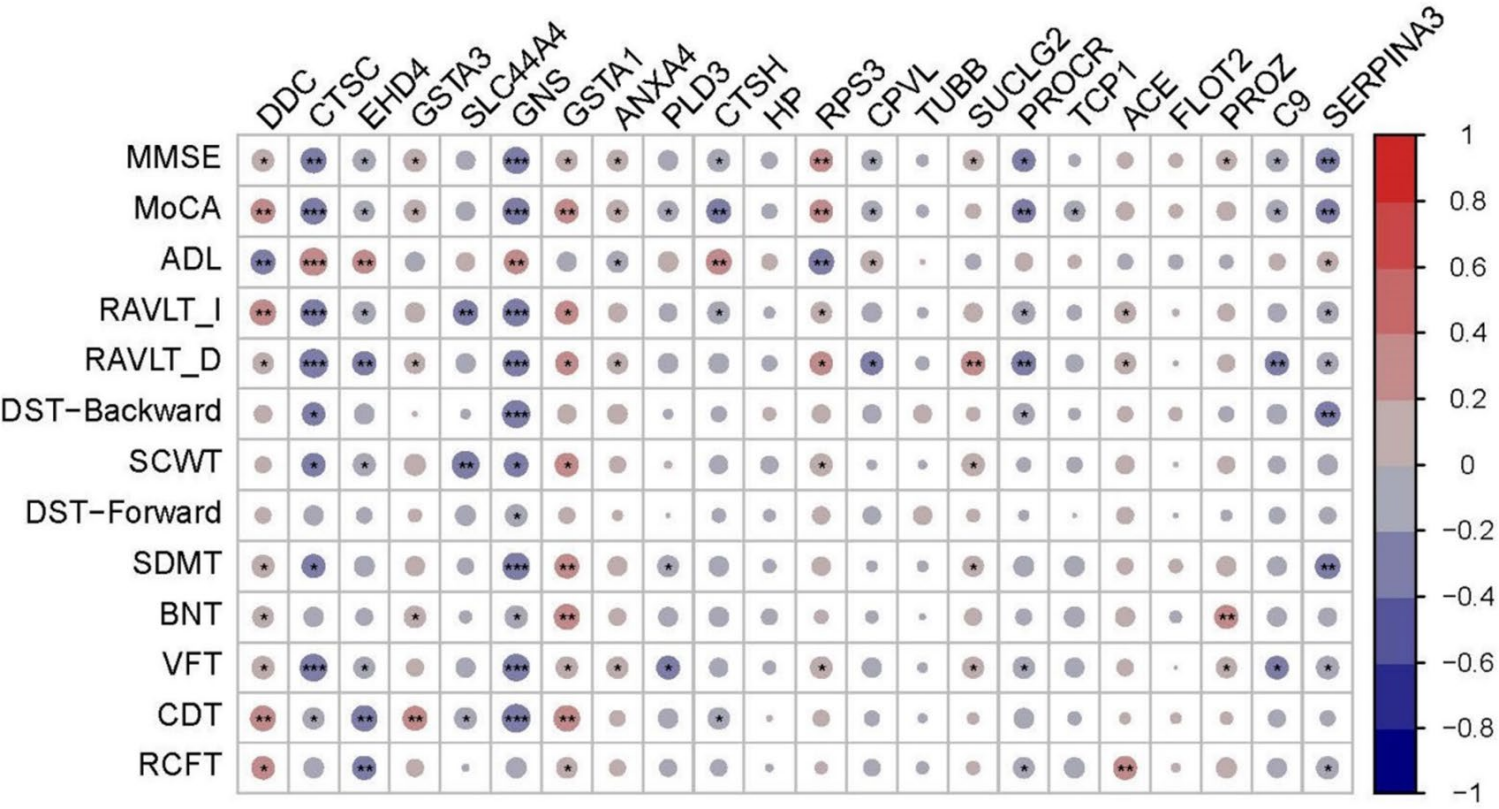
Correlation heatmap between diagnostic proteins and cognition tests. *p < 0.05; **p < 0.01; ***p < 0.001

Diagnostic proteins were found to be correlated with cognitive tests, although most weakly (Fig. 5). Significant labels were shown on the dots. Among 22 diagnostic proteins, DDC, CTSC, EHD4, GNS, GSTA1, RPS3, PROCR, and SERPINA3 were significantly correlated with more than half of cognitive tests while GSTA3, SLC44A4, ANXA4, PLD3, CTSH, CPVL, SUCLG2, TCP1, ACE, PROZ, and C9 were significantly correlated with less than half cognitive tests. Nevertheless, none of the correlations between HP, TUBB, or FLOT2 and cognitive domains reach significance. The relative *ρ* and *p* were shown in Additional file 10: Table S5, and scatter dot plots were shown in Additional file 5: Fig. S5.

## Discussion

In this research, we firstly enrolled 57 AD patients, 43 MCI patients, and 62 CN subjects from China-Japan Friendship Hospital from April 2022 to November 2022, collected urine samples, and conducted an LC–MS/MS analysis. Consistent with previous results, age and APOE ε4 status were remarkable risk factors. Most cognitive tests differed in three groups, and qEEG and MTA scales differed between the AD and CN groups. Then, we reported the identified urine proteins, constructed a PPI network, and conducted differential analysis. There was a total of 608 proteins included in the analysis with which 33 significantly differential proteins between the AD and CN groups, including 21 upregulated ones and 12 downregulated ones. In parallel, there were 15 significantly differential proteins between the MCI and CN groups, including 7 upregulated ones and 8 downregulated ones. Next, we attempted to figure out the novel diagnostic panels based on the LASSO and SVM models. AD diagnostic panel achieved an AUC of 0.8824 in the test set while MCI diagnostic panel achieved an AUC of 0.8143 in the test set. Finally, we conducted a correlation analysis and found that diagnostic proteins were weakly correlated with cognitive functions.

As for basic information collection, different from previous research [3], only the distribution of age and APOE ε4 status varied among the three groups. The difference might be caused by the sample size and the representativeness of samples, such as sources of the patients, in which our research was based on a general hospital in Beijing. As for clinical characteristics, the results of cognitive tests, qEEG, and MRI significantly differed in the three groups which indicated the reliability of our clinical diagnosis.

There were few studies investigating the role of urine proteins in AD. Watanabe et al. [39] identified a total of 1705 unique proteins in 18 AD and 18 controls while only 578 proteins were identified in at least half samples of either group. The number of proteins appearing in half of the samples was similar to our result. Besides, Chen et al. [40] identified 4157 proteins in 9 AD patients and 3977 proteins in 21 normal controls (NC). However, they focused on VaD which compared the results of VaD to AD and NC.

In our study, we identified 2 diagnostic panels. As for AD diagnosis, DDC was reported to elevate in the CSF of Aβ- and p-tau-positive patients compared to controls [41]. CTSC was defined as a risk factor for AD by GWAS which was significantly upregulated in the App^NL−G−F/NL−G−F^ cortex [42, 43]. GSTA3 was significantly elevated in AD rats’ hippocampus by using label-free nano-LC–MS/MS which further speculated the role of diagnosis mechanism and drug discovery [44]. Besides, PLD3 was suggested to be the gene that increases AD risk [45–47] and was downregulated in AD brains which might participate in AD pathogenesis through amyloid precursor protein (APP) processing [48, 49]. PLD3 affected axonal spheroids and network defects in AD [50]. Moreover, in another bioinformatics research, HP was also identified as playing a significant role [51]. In human samples, higher serum levels of HP were observed in AD [52, 53] and MCI [52] patients than controls. Findings from Philbert et al. [54] indicated a pervasive underlying mechanism in which micro-vasculopathy promoted erythrocyte leakage, elevating tissue-free hemoglobin and causing the observed increases in HP in the brains of sporadic AD while Cigliano et al. found that HP interacted with APOE and Aβ and influenced their crosstalk [55]. In rat hippocampus, HP increased with age while further in the U-87 MG cell line, HP was proved to influence Aβ peptide aggregation or clearance [56]. Nevertheless, we failed to search the articles reporting the relationship between EHD4, SLC44A4, GNS, GSTA1, CSTH, RPS3 or CPVL, and AD.

As for MCI diagnosis, there was little research reporting the direct relationship between diagnostic proteins and MCI except for ACE. ACE D-allele may be a genetic risk factor for cognition which increased serum ACE levels [57, 58], and ACE inhibitor is a protective factor against cognitive decline [59]. However, in the continuum of MCI progression, several proteins were suggested to be involved in AD which shares similar alterations. TUBB was identified as a hub gene in AD [60] while according to covalent protein painting, the accessibility of lysine residues for covalent modification in TUBB was altered in human postmortem brain samples of AD patients [61]. By integrating human cortex, CSF, and serum proteomic datasets, SUCLG2 was prioritized as one of the most promising AD signature proteins [62]. Our results provide additional data to the above conclusion. Besides, SUCLG2 (rs62256378) was found to be associated with Aβ1–42 level, and functional microglia experiments showed that SUCLG2 participated in Aβ1–42 clearance [63]. Serum-soluble PROCR levels were higher in AD patients compared with controls while the difference between MCI patients and healthy controls or AD did not reach statistical significance [64]. Moreover, SERPINA3 was identified as a marker gene in AD [65].

In general, some diagnostic proteins were measured in other samples, and some diagnostic proteins were studied in functional studies while the relationship between some diagnostic proteins with AD and MCI remained relatively unexplored. The expression levels of diagnostic proteins in other samples may be consistent or inconsistent with the status in urine, which may be due to gene regulation of expression or to imbalance in urinary excretion. Also, the result may indicate that changes in urine are more sensitive in the early stages of the disease. This suggests that more research is required to determine the mechanisms.

As for the weak correlations among diagnostic proteins and different cognitive domains, generally speaking, compared to laboratory tests, the results of the neuropsychological scales are subjective. There may be situations where patients did not cooperate, or there may be deviations due to the tester’s different judgment. In this case, urine protein results can be used for auxiliary diagnosis, and the results will be more objective, making the diagnostic basis more sufficient.

Due to some limitations, our findings should be reported with caution. First, the patients came from a single site. We lacked real-world research from multiple hospitals and communities. Whether the findings can be applicable to other populations, more research is required. Second, the proteins identified in more than 50% of the samples were relatively few. Detection methods and data processing methods should be improved. Third, no in vivo or in vitro experiments were conducted to investigate the mechanisms of the diagnostic proteins described in this study that participate in AD pathophysiological processes. Besides, one thing to note is that machine learning steps used differential proteins derived from the whole dataset, and therefore, the performance estimation on the test set might be optimistic. Thus, some of these results may be coincidental.

## Conclusions

In conclusion, we performed proteomics analysis based on LC–MS/MS using urine samples from 57 AD patients, 43 MCI patients, and 62 CN subjects. After multiple traditional statistical analyses and bioinformatics analyses, we identified a novel AD diagnostic panel that included DDC, CTSC, EHD4, GSTA3, SLC44A4, GNS, GSTA1, ANXA4, PLD3, CTSH, HP, RPS3, CPVL, age, and APOE ε4 and an MCI diagnostic panel which included TUBB, SUCLG2, PROCR, TCP1, ACE, FLOT2, EHD4, PROZ, C9, SERPINA3, age, and APOE ε4. The urine diagnostic panel could help clinicians differentiate AD and MCI from CN, the method of which is convenient, non-invasive, and valuable for diagnosis.

### Supplementary Information

**Additional file 1: Fig. S1.** GSEA results for all proteins included for analysis (*p*<0.05). AD-CN A-D The results of the AD-CN group. A. Biological processes enriched in AD-CN group; B. Cellular components enriched in AD-CN group; C. Molecular functions enriched in AD-CN group; D. Kegg pathway enriched in AD-CN group. MCI-CN The results of the MCI-CN group. A. Biological processes enriched in MCI-CN group; B. Cellular components enriched in MCI-CN group; C. Molecular functions enriched in MCI-CN group; D. Kegg pathway enriched in MCI-CN group. AD-MCI A-D The results of the AD-MCI group. A. Biological processes enriched in AD-MCI group; B. Cellular components enriched in AD-MCI group; C. Molecular functions enriched in AD-MCI group; D. Kegg pathway enriched in AD-MCI group.

**Additional file 2: Fig. S2.** Differentially urinary proteins in the AD-MCI group. A. Heatmap of total of 19 differential proteins between AD and MCI. B. Volcano plot showed the distribution of all proteins between AD and MCI.

**Additional file 3: Fig. S3.** Venn diagram showing the intersection among different groups.

**Additional file 4: Fig. S4.** GO biological processes enrichment network in AD and MCI compared to CN group. A. Enrichment network in AD-CN group. B. Enrichment network in MCI-CN group. Yellow nodes indicated significant enriched processes (Benjamini-Hochberg corrected *p*-value<0.05).

**Additional file 5: Fig. S5.** Scatter plots of different diagnostic proteins with different cognition tests.

**Additional file 6: Table S1.** Basic information and individual tests results of each participant.

**Additional file 7: Table S2.** Identified urine proteins from enrolled patients. Sheet1. Raw data of all identified proteins. Sheet2. Included total of 608 proteins measured in more than half samples. The dataset was complemented using KNN methods.

**Additional file 8: Table S3.** A table with the log2FC, *p*-values and corrected p-values of the 608 proteins included in the analysis. Sheet 1. AD-CN group; Sheet 2. MCI-CN group; Sheet 3. AD-MCI group.

**Additional file 9: Table S4.** The GO biological processes enrichment details of differential proteins. Sheet 1. AD-CN group; Sheet 2. MCI-CN group.

**Additional file 10: Table S5.** Spearman correlation between diagnostic proteins and cognition tests. Relative correlation coefficient ρ and significance *p* (two-sided).

## Data Availability

All data generated or analyzed during this study are included in this published article and its supplementary information files.The mass spectrometry proteomics data have been deposited to the ProteomeXchange Consortium (http://proteomecentral.proteomexchange.org) via the iProX partner repository with the dataset identifier PXD044672.

## Abbreviations

AD: Alzheimer’s disease
MCI: Mild cognitive impairment
Aβ: Amyloid-β
SPP1: Secreted phosphoprotein 1
GSN: Gelsolin
IFFBP7: Insulin-like growth factor binding protein 7
AD7c-NTP: Alzheimer-associated neuronal thread protein
ApoC3: Apolipoprotein C3
ELISA: Enzyme-linked immunosorbent assay
CN: Cognitive normal
APOE: Apolipoprotein E
qEEG: Quantitative electroencephalography
MRI: Magnetic resonance imaging
NIA-AA: National Institute on Aging-Alzheimer’s Association
FTD: Frontotemporal dementia
LBD: Lewy body dementia
VaD: Vascular dementia
MMSE: Mini-Mental State Examination
MoCA: Montreal Cognitive Assessment
ADL: Activity of Daily Living Scale
RAVLT-I: Rey Auditory Verbal Learning Test-Immediate
RAVLT-D: Rey Auditory Verbal Learning Test-Delay
DST: Digit Span Test
SCWT: Stroop Color and Word Test
TMT: Trail Making Test
SDMT: Symbol Digit Modalities Test
BNT: Boston Naming Test
VFT: Verbal Fluency Test
CDT: Clock Drawing Test
RCFT: Rey Complex Figure Test
DTT: Dithiothreitol
LC–MS/MS: Liquid chromatography coupled to tandem mass spectrometry
DDA: Data-dependent acquisition
AGC: Automatic gain control
iBAQ: Intensity-based absolute quantification
FOT: Fraction of total
KNN: *k*-Nearest neighbor
ANOVA: Analysis of variance
PPI: Protein-protein interaction
GSEA: Gene set enrichment analysis
GO: Gene Ontology
KEGG: Kyoto Encyclopedia of Genes and Genomes
LASSO: Least absolute shrinkage and selection operator
MSE: Mean square error
SVM: Support vector machine
AUC: Area under the curve
ROC: Receiver operating characteristic
MTA: Medial Temporal Lobe Atrophy Scale
DDC: Dopa decarboxylase
CTSC: Cathepsin C
EHD4: EH domain containing 4
GSTA3: Glutathione S-transferase alpha 3
SLC44A4: Solute carrier family 44 member 4
GNS: Glucosamine (N-acetyl)-6-sulfatase
GSTA1: Glutathione S-transferase alpha 1
ANXA4: Annexin A4
PLD3: Phospholipase D family member 3
CTSH: Cathepsin H
HP: Haptoglobin
RPS3: Ribosomal protein S3
CPVL: Carboxypeptidase vitellogenic like
TUBB: Tubulin beta class I
SUCLG2: Succinate-CoA ligase GDP-forming subunit beta
PROCR: Protein C receptor
TCP1: T-complex 1
ACE: Angiotensin I-converting enzyme
FLOT2: Flotillin 2
PROZ: Protein Z: vitamin K-dependent plasma glycoprotein
C9: Complement C9
SERPINA3: Serpin family A member 3
NC: Normal controls
APP: Amyloid precursor protein
