# Supplementary figures and images for: Identification of novel diagnostic panel for mild cognitive impairment and Alzheimer’s disease: findings based on urine proteomics and machine learning
Source: Alzheimers Res Ther. 2023 Nov 4;15:191. doi: 10.1186/s13195-023-01324-4 (PMC10625308; doi:10.1186/s13195-023-01324-4)

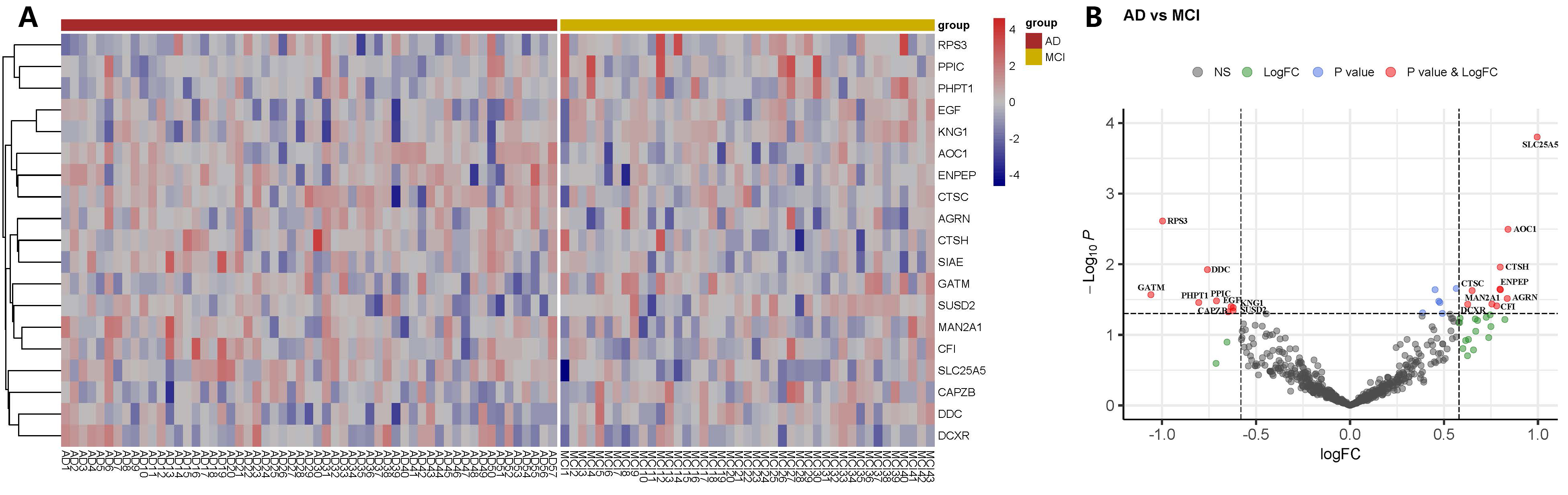

Supplement: Supplementary file 2 — Additional file 2: Fig. S2. Differentially urinary proteins in the AD-MCI group. A. Heatmap of total of 19 differential proteins between AD and MCI. B. Volcano plot showed the distribution of all proteins between AD and MCI. [file 13195_2023_1324_MOESM2_ESM.jpg]

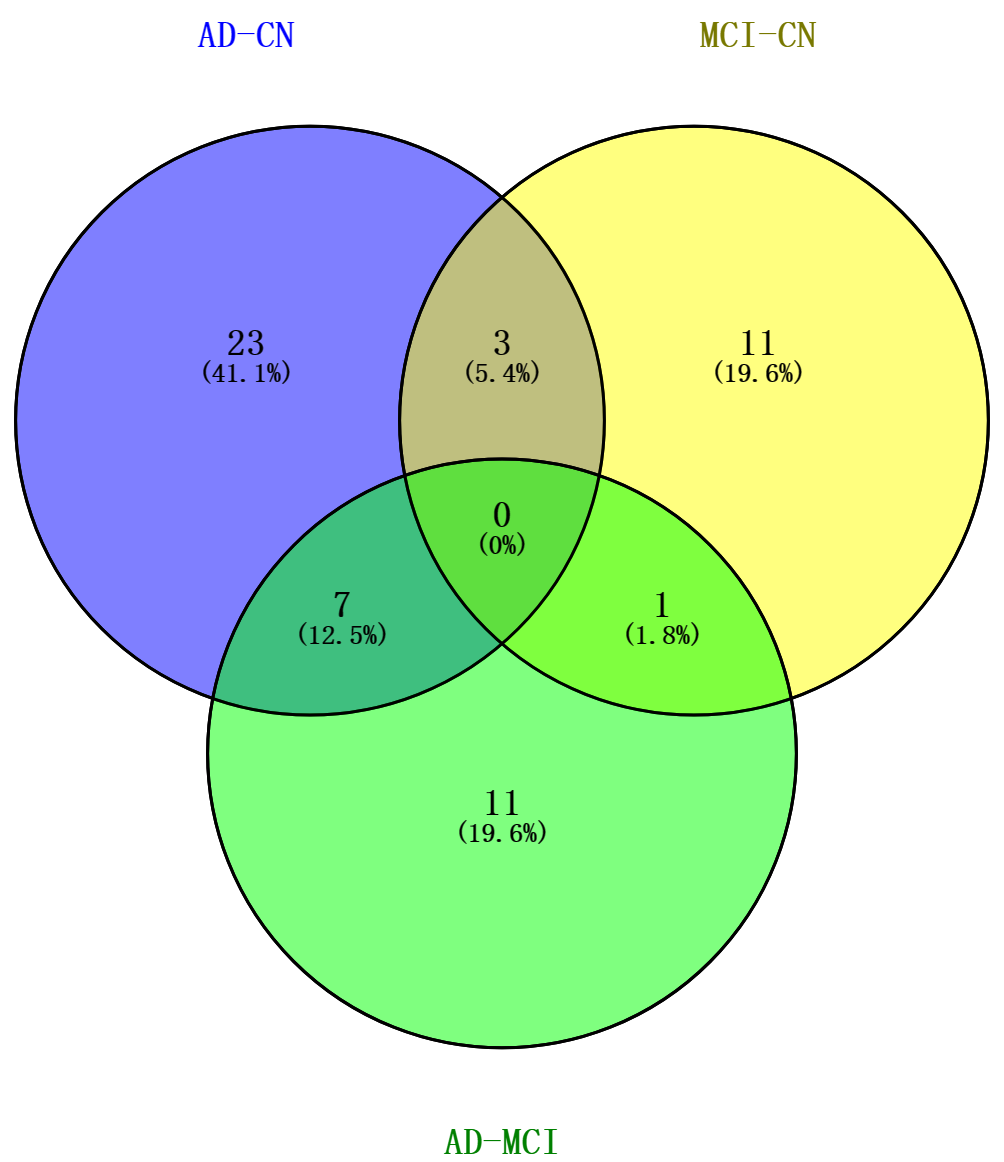

Supplement: Supplementary file 3 — Additional file 3: Fig. S3. Venn diagram showing the intersection among different groups. [file 13195_2023_1324_MOESM3_ESM.png]

A

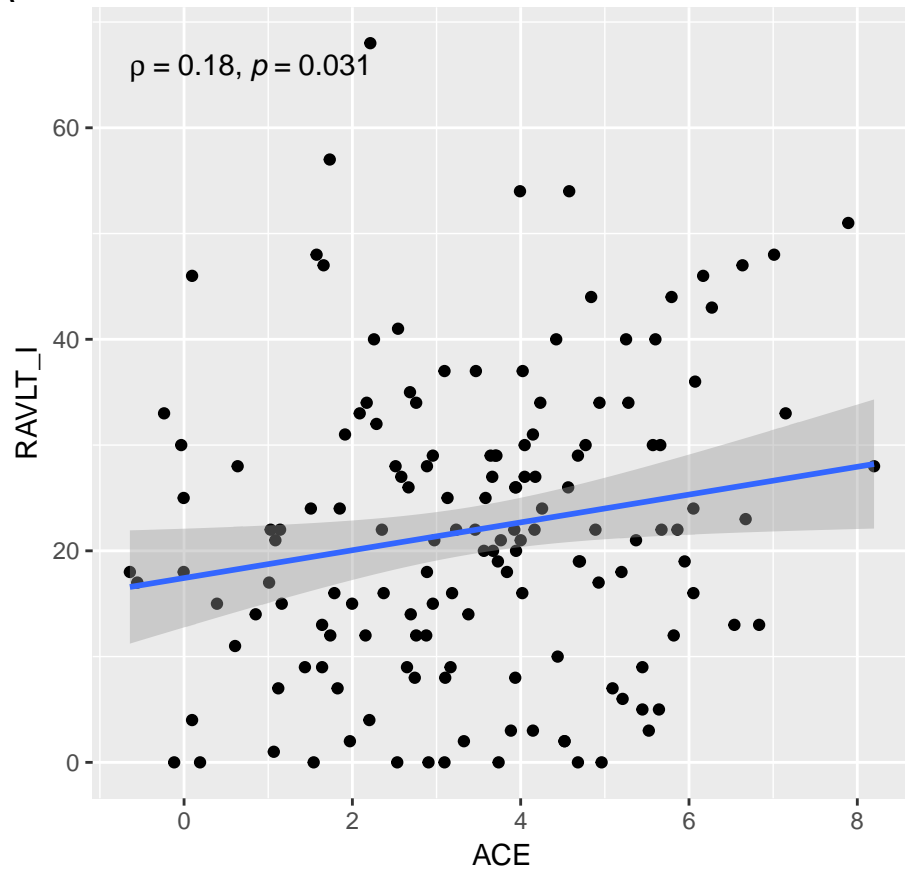

B

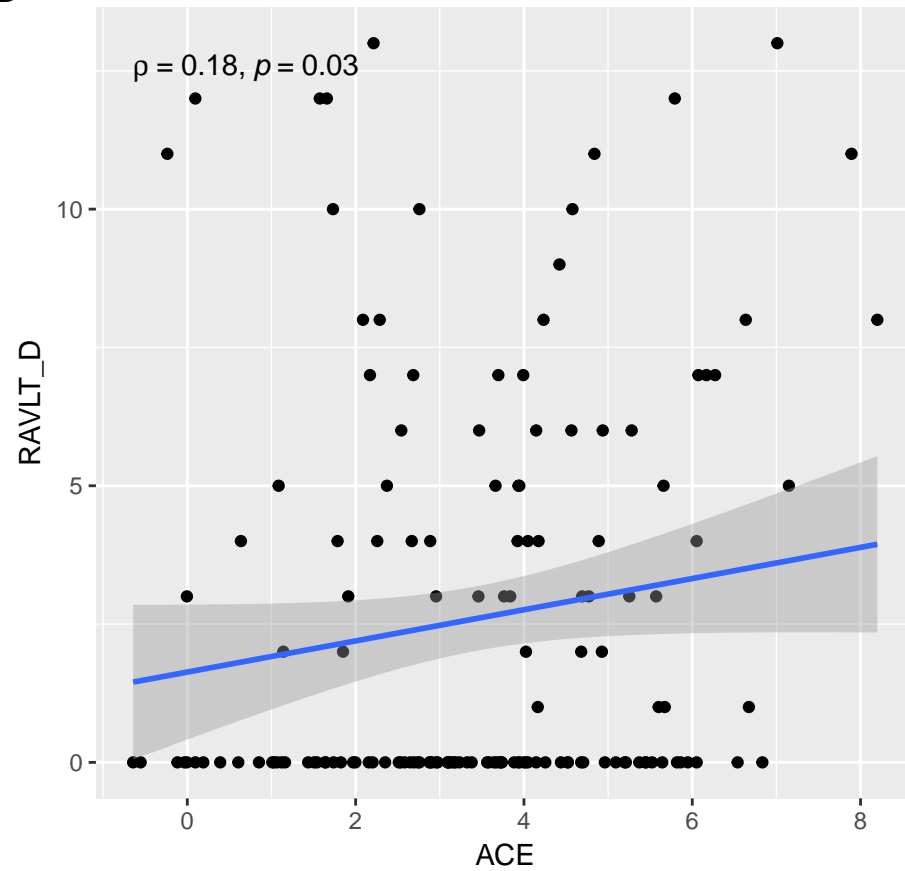

C

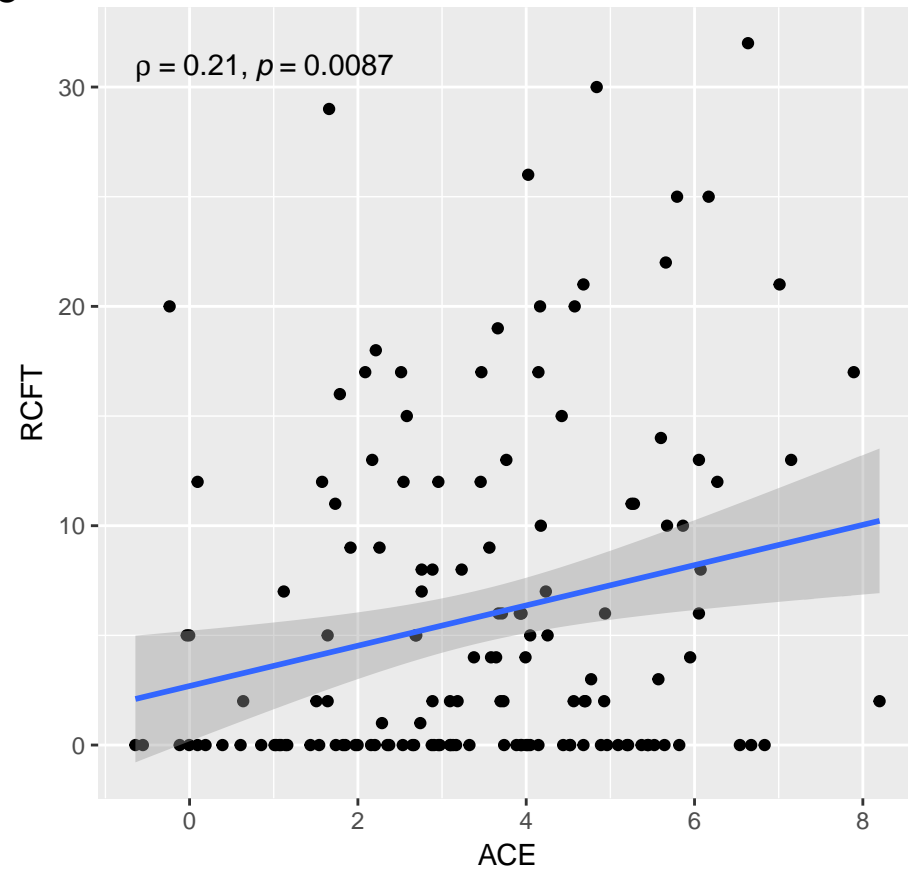

Supplement: Supplementary file 5 — Additional file 5: Fig. S5. Scatter plots of different diagnostic proteins with different cognition tests. [file 13195_2023_1324_MOESM5_ESM.zip › additional Fig 5-ACE.pdf]

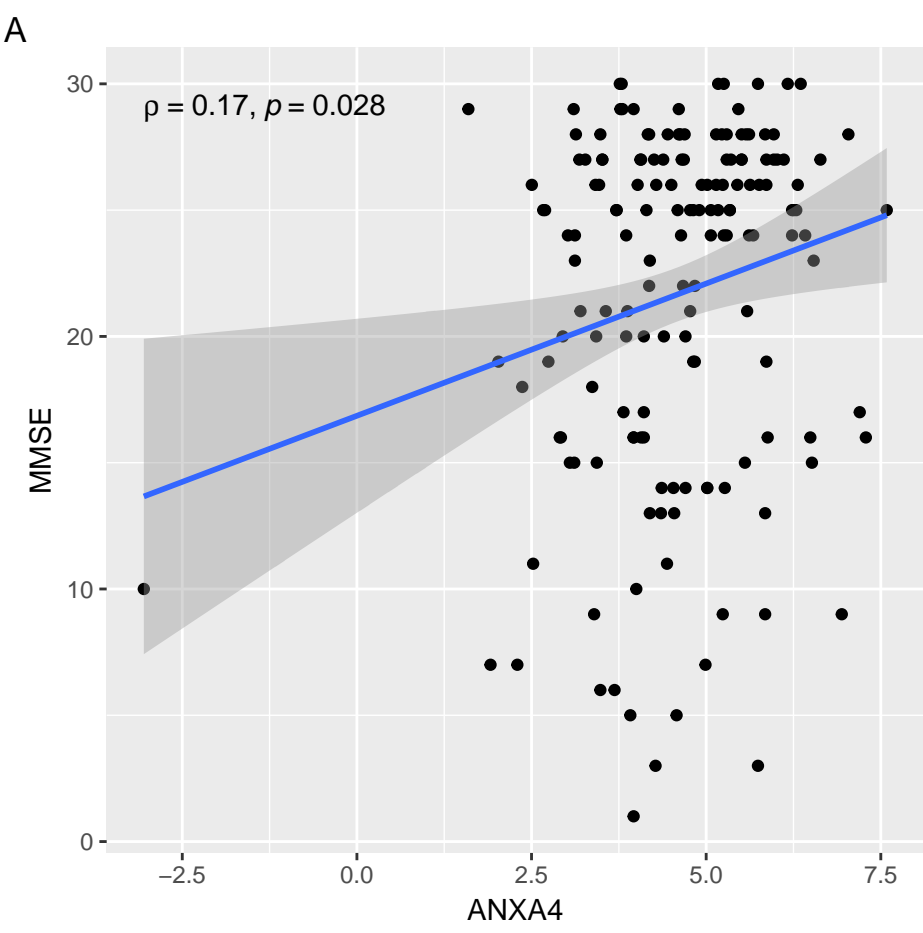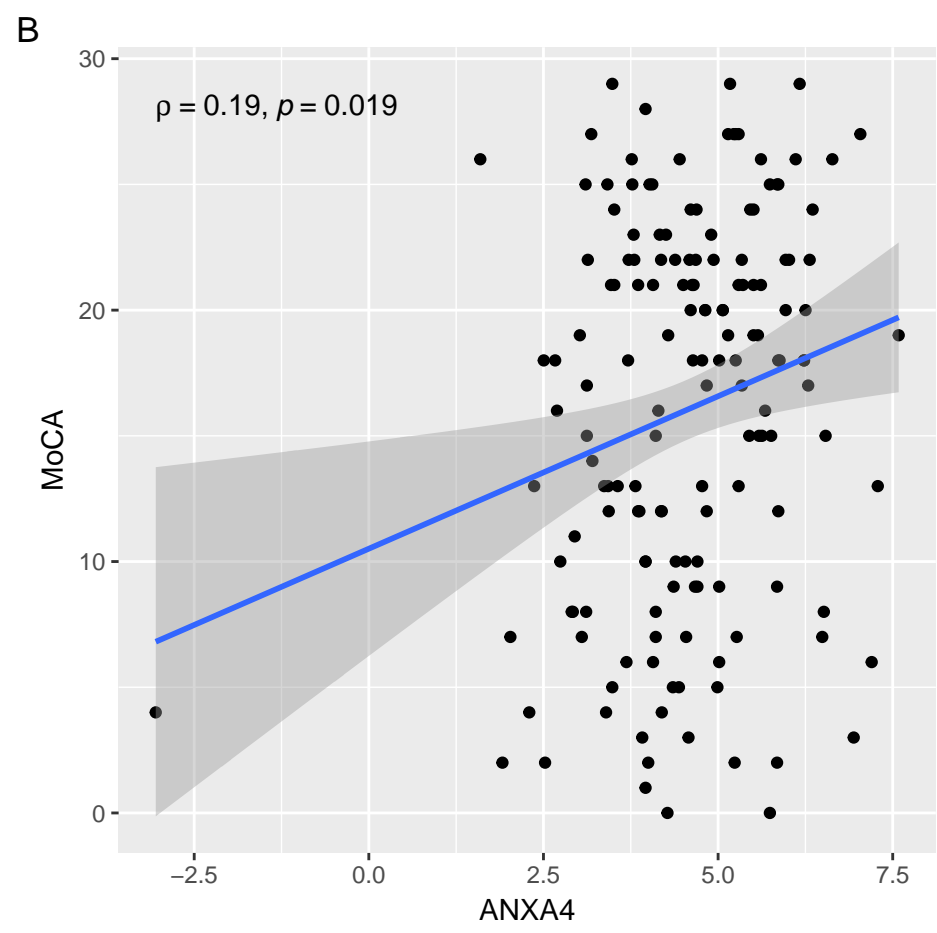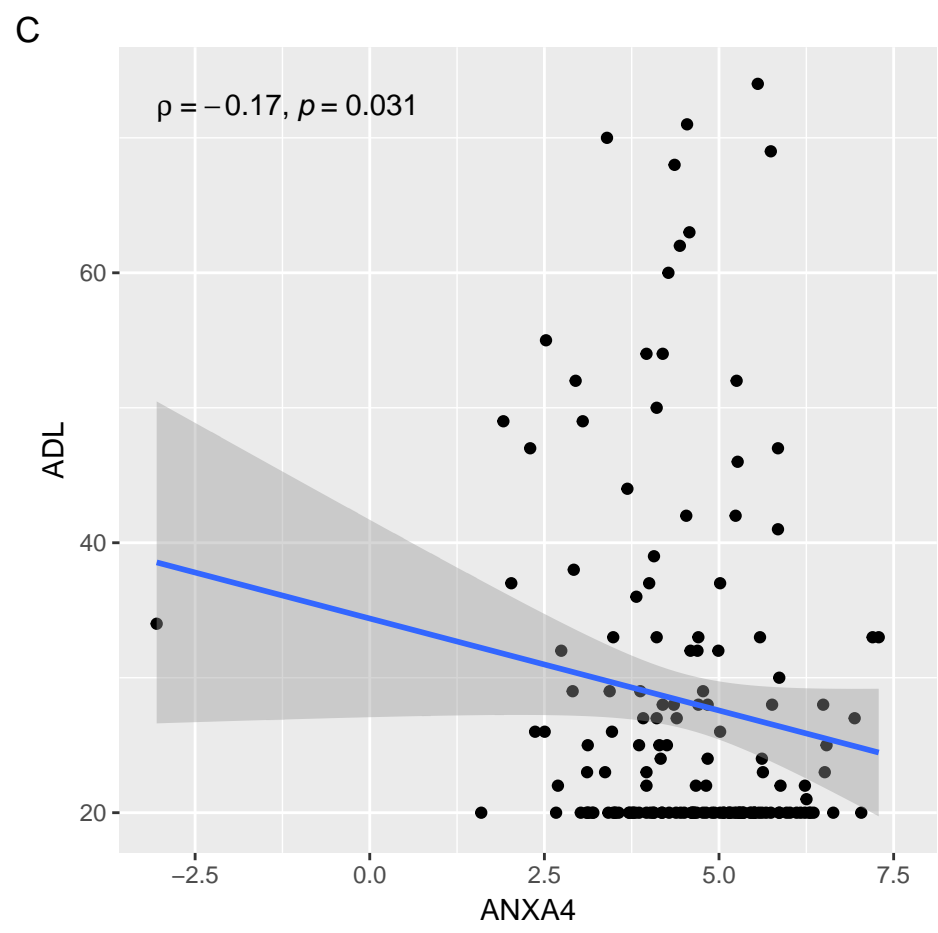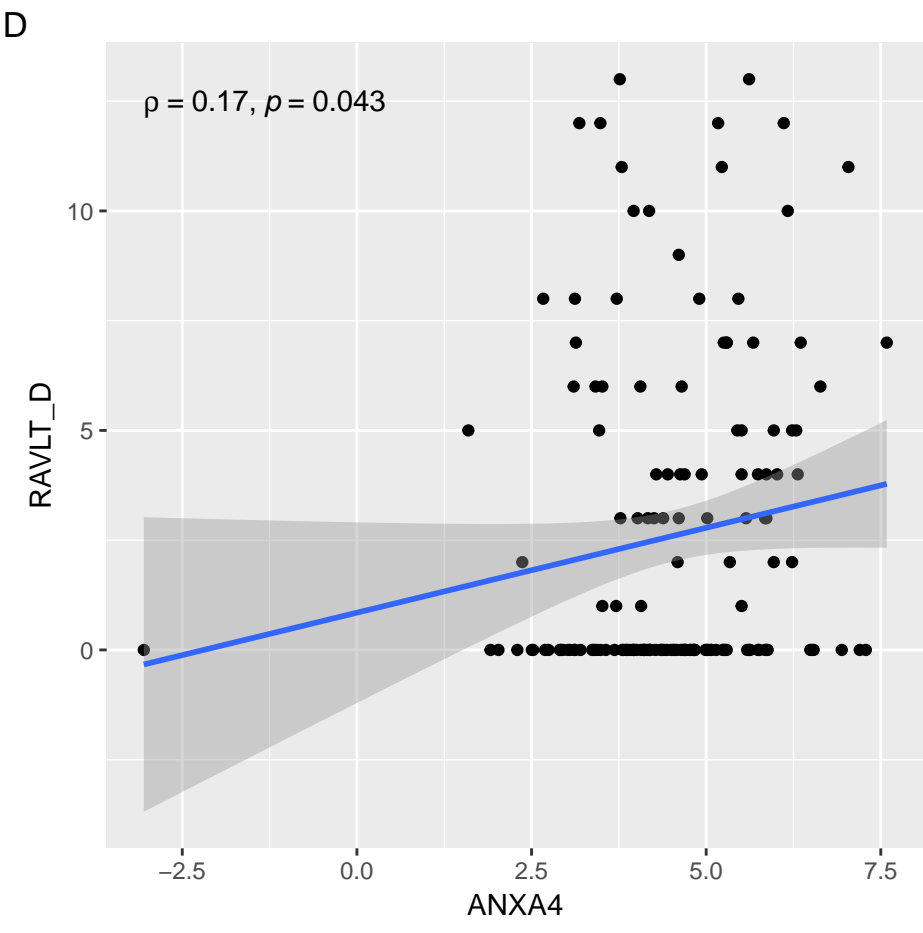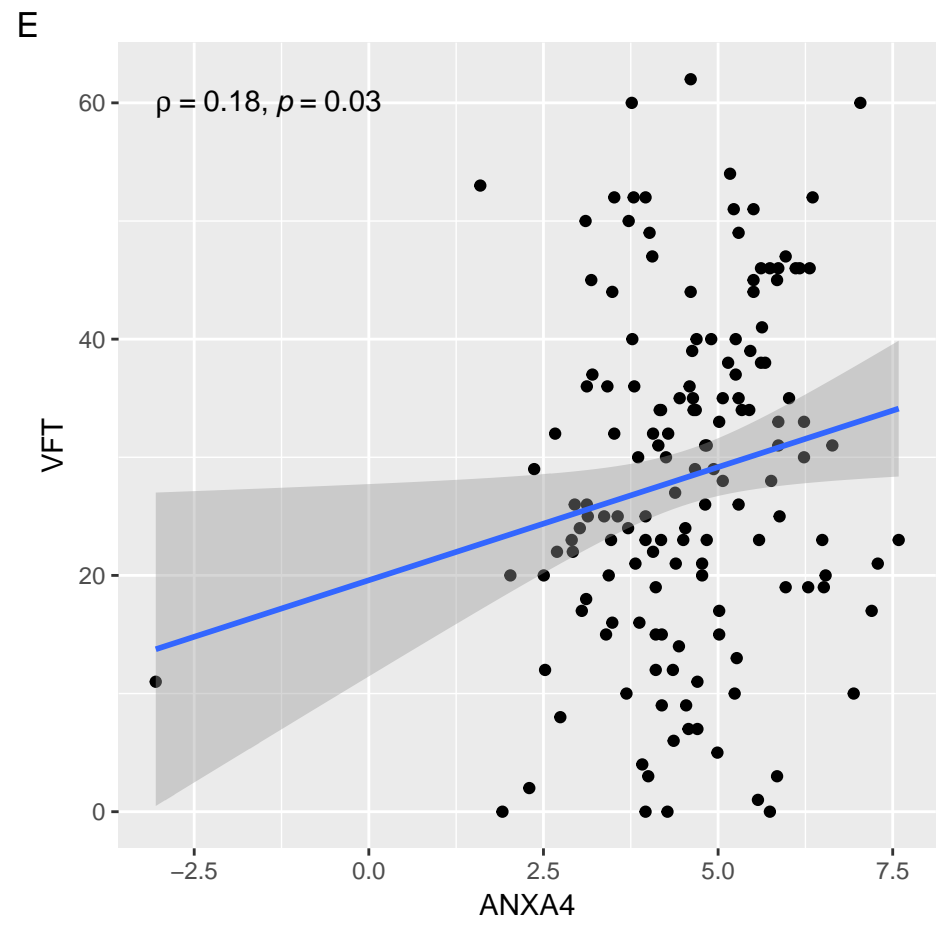

Supplement: Supplementary file 5 — Additional file 5: Fig. S5. Scatter plots of different diagnostic proteins with different cognition tests. [file 13195_2023_1324_MOESM5_ESM.zip › additional Fig 5-ANXA4.pdf]

A

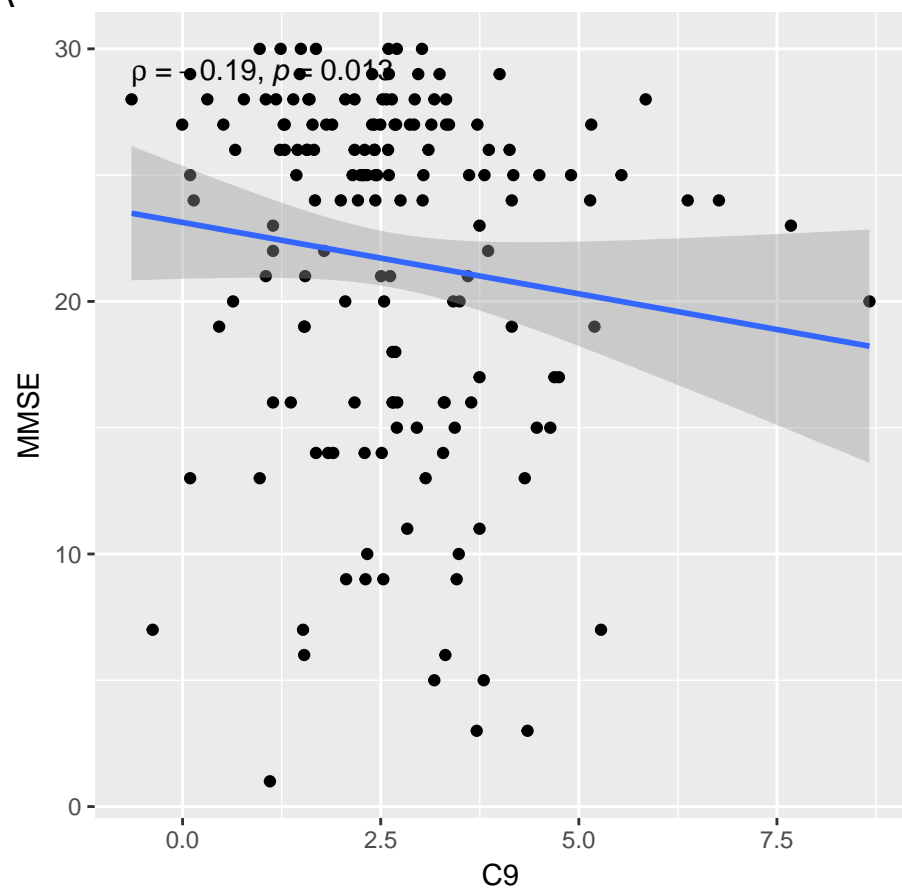

B

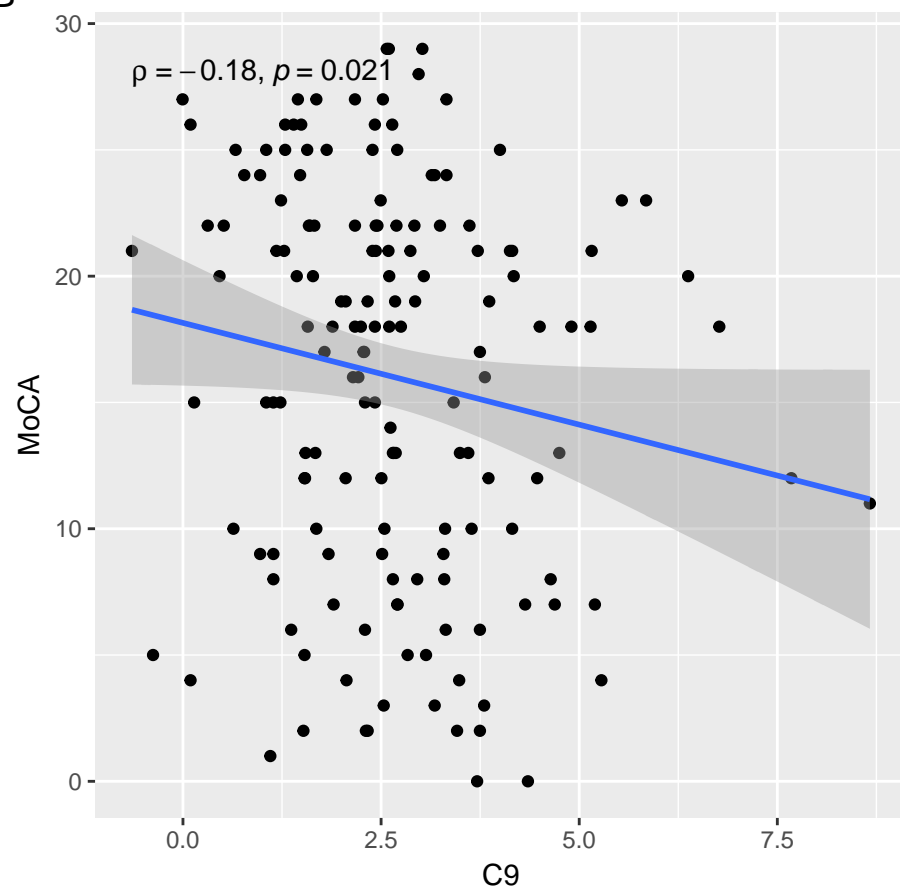

C

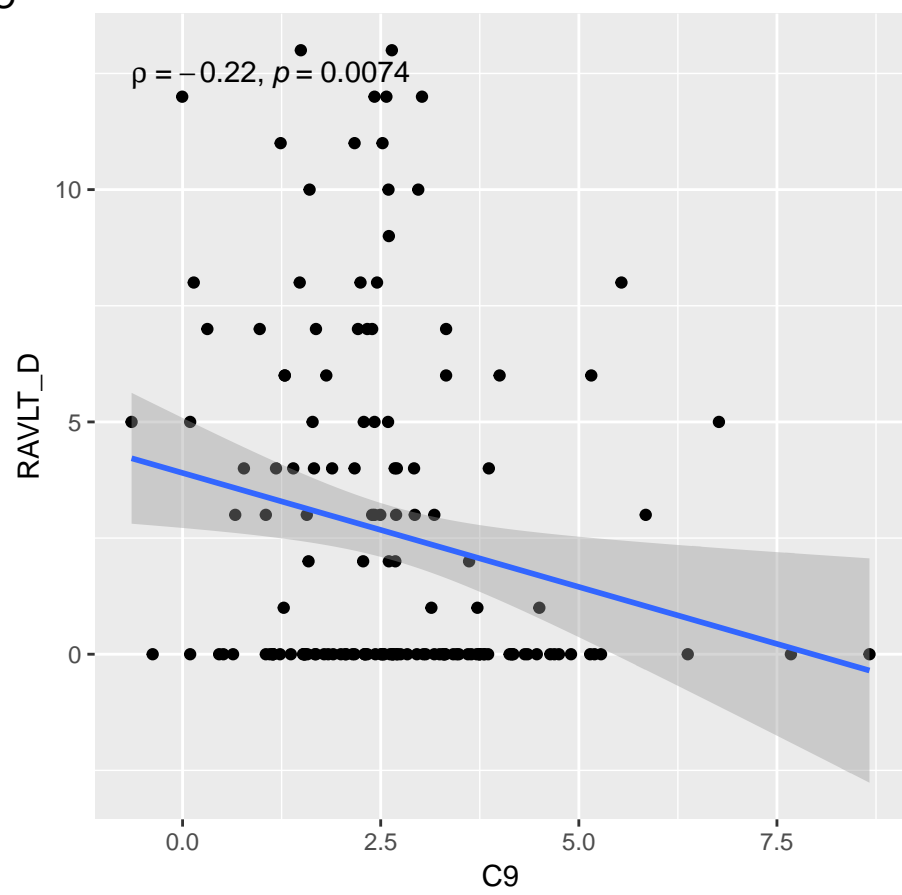

D

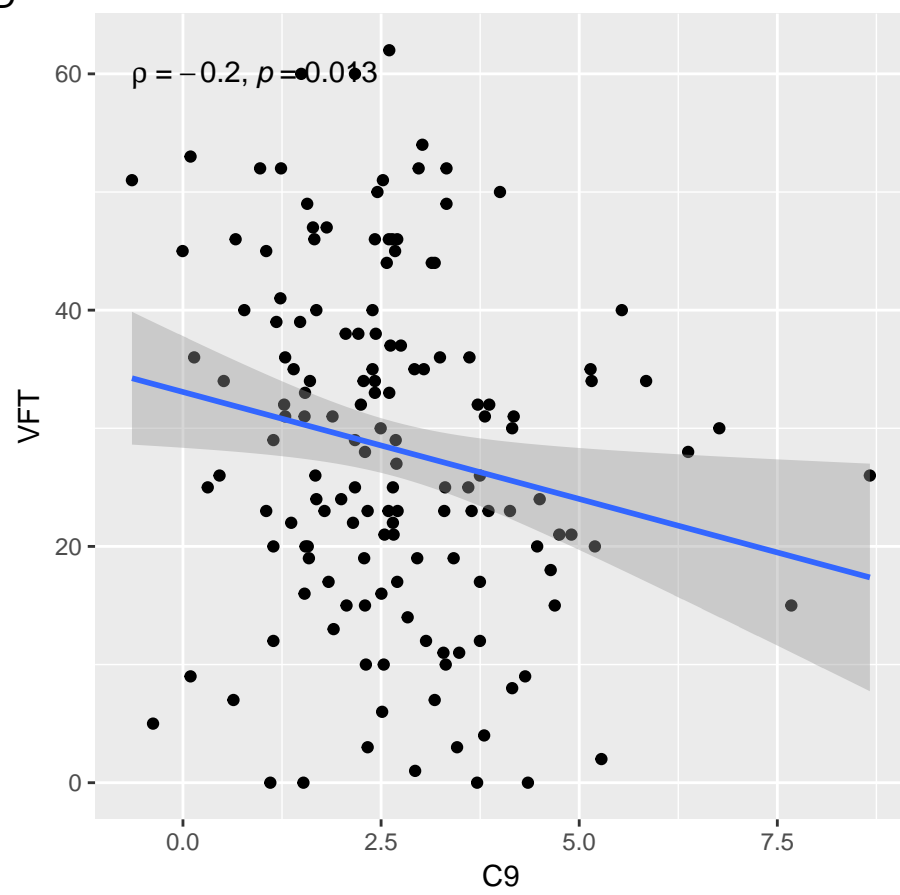

Supplement: Supplementary file 5 — Additional file 5: Fig. S5. Scatter plots of different diagnostic proteins with different cognition tests. [file 13195_2023_1324_MOESM5_ESM.zip › additional Fig 5-C9.pdf]

A

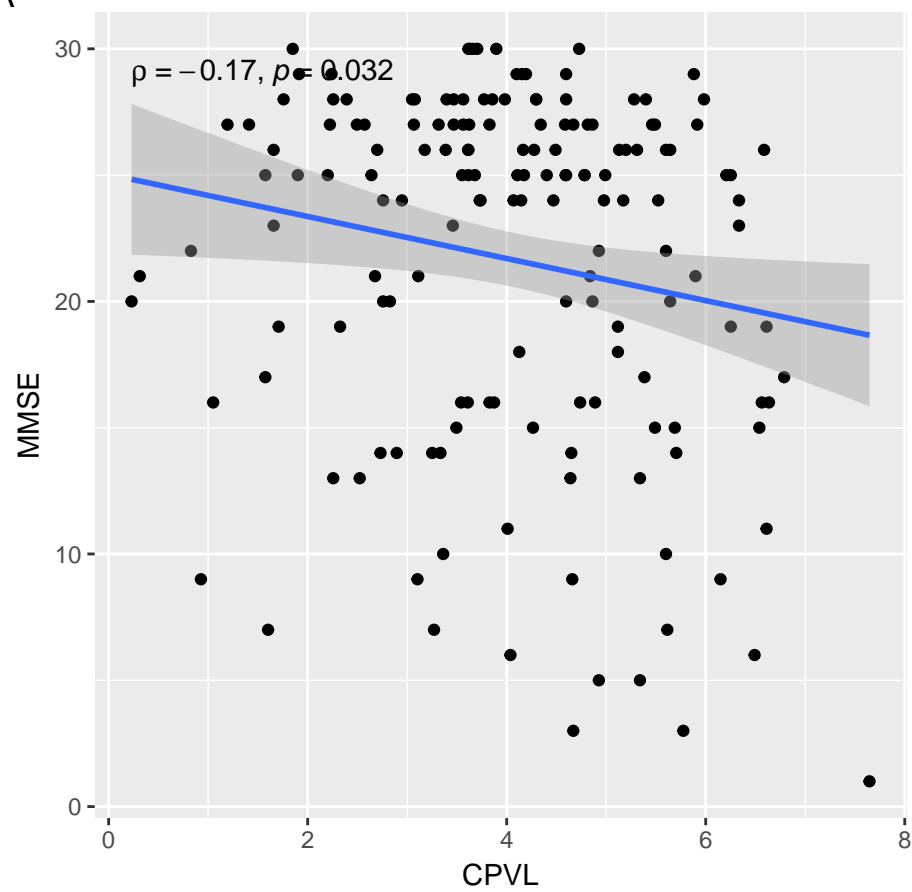

B

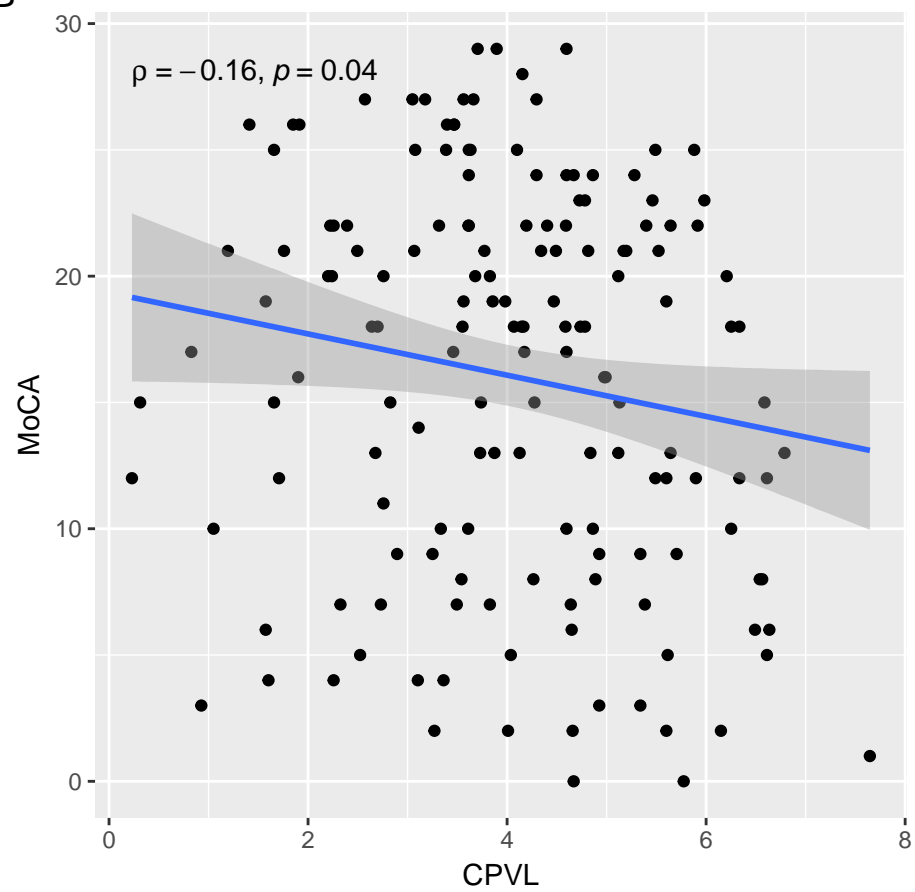

C

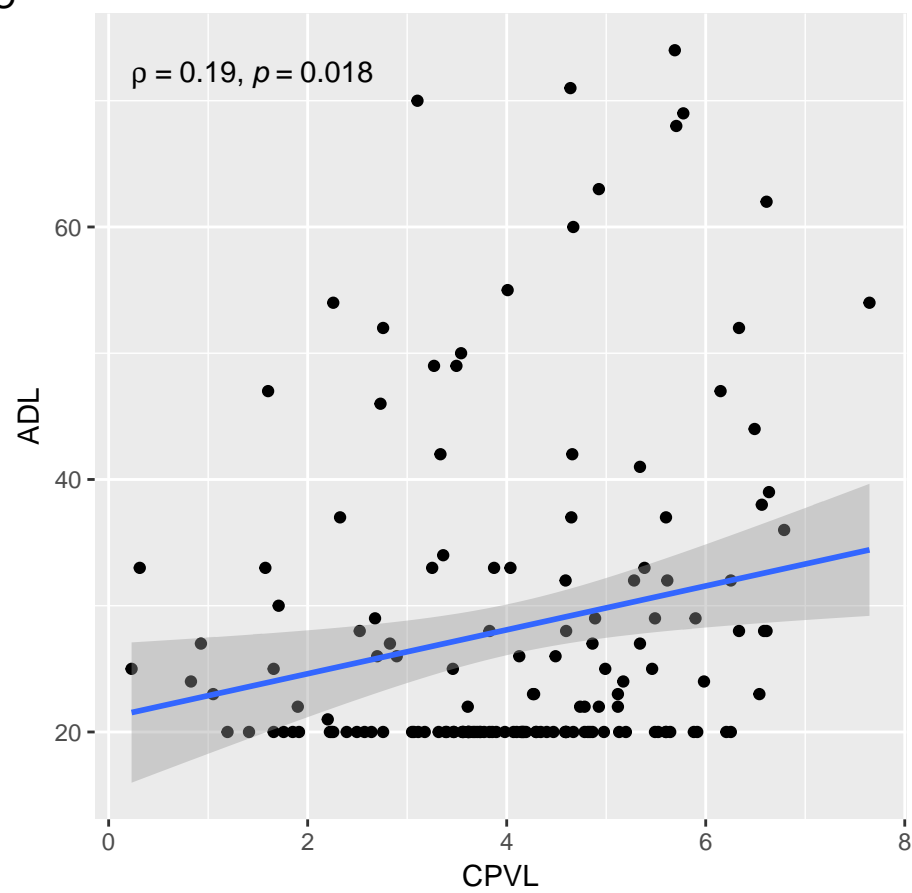

D

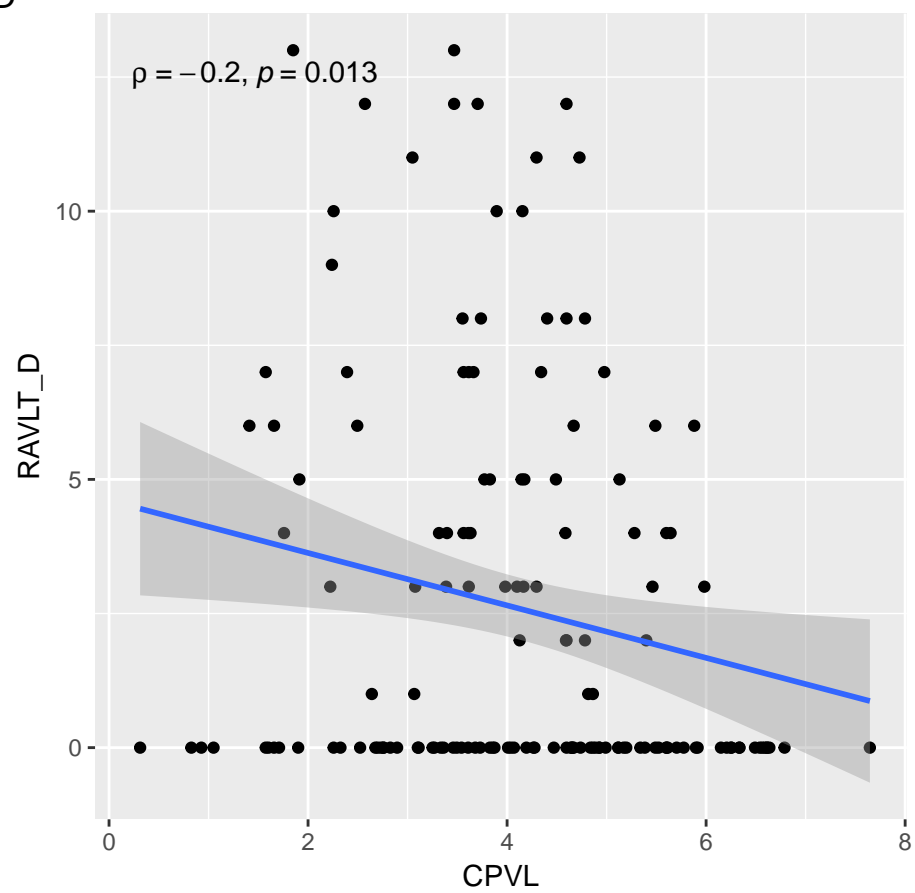

Supplement: Supplementary file 5 — Additional file 5: Fig. S5. Scatter plots of different diagnostic proteins with different cognition tests. [file 13195_2023_1324_MOESM5_ESM.zip › additional Fig 5-CPVL.pdf]

**A**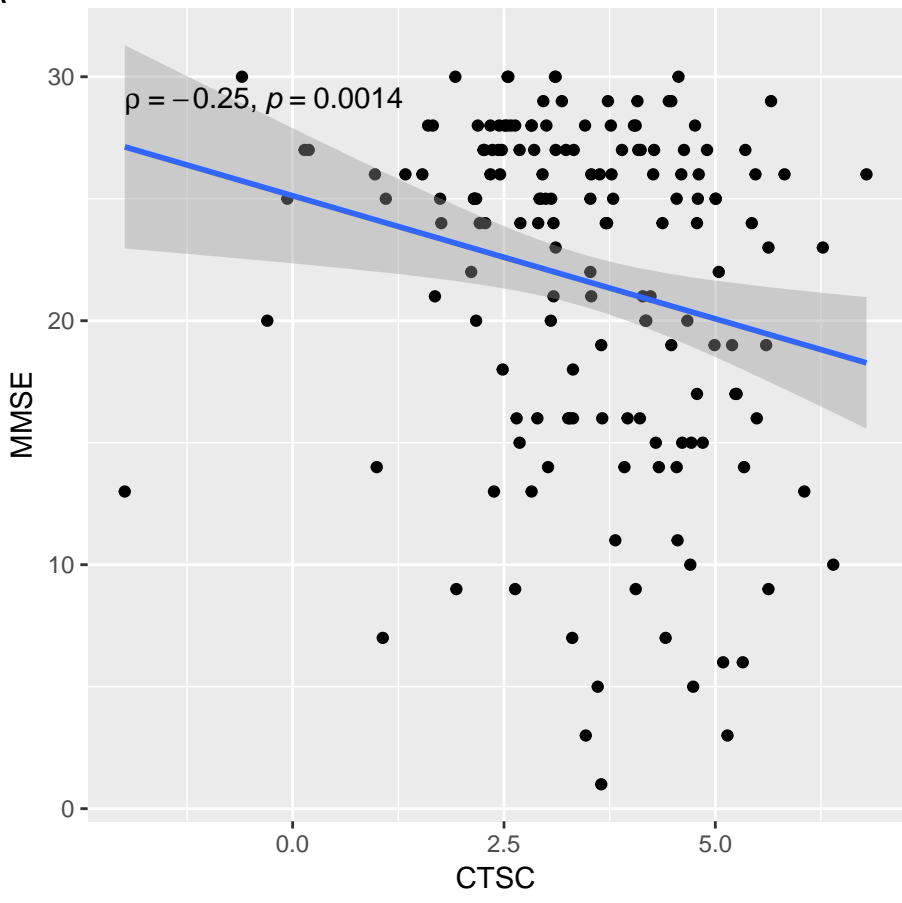**B**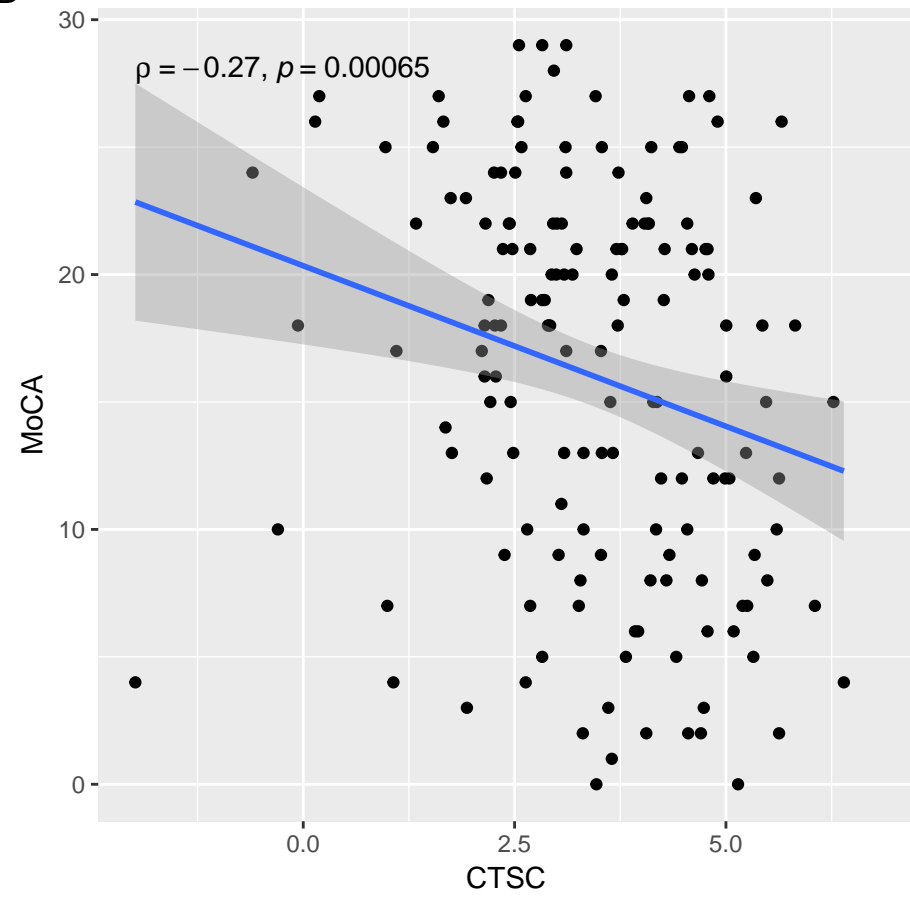**C**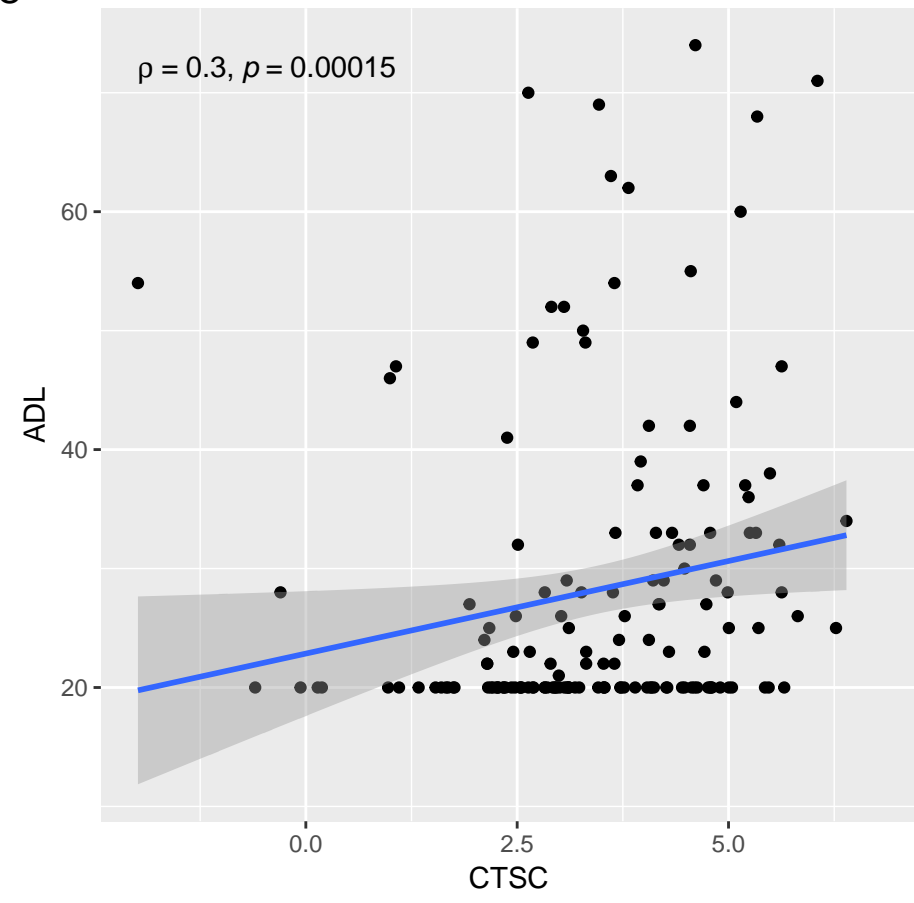**D**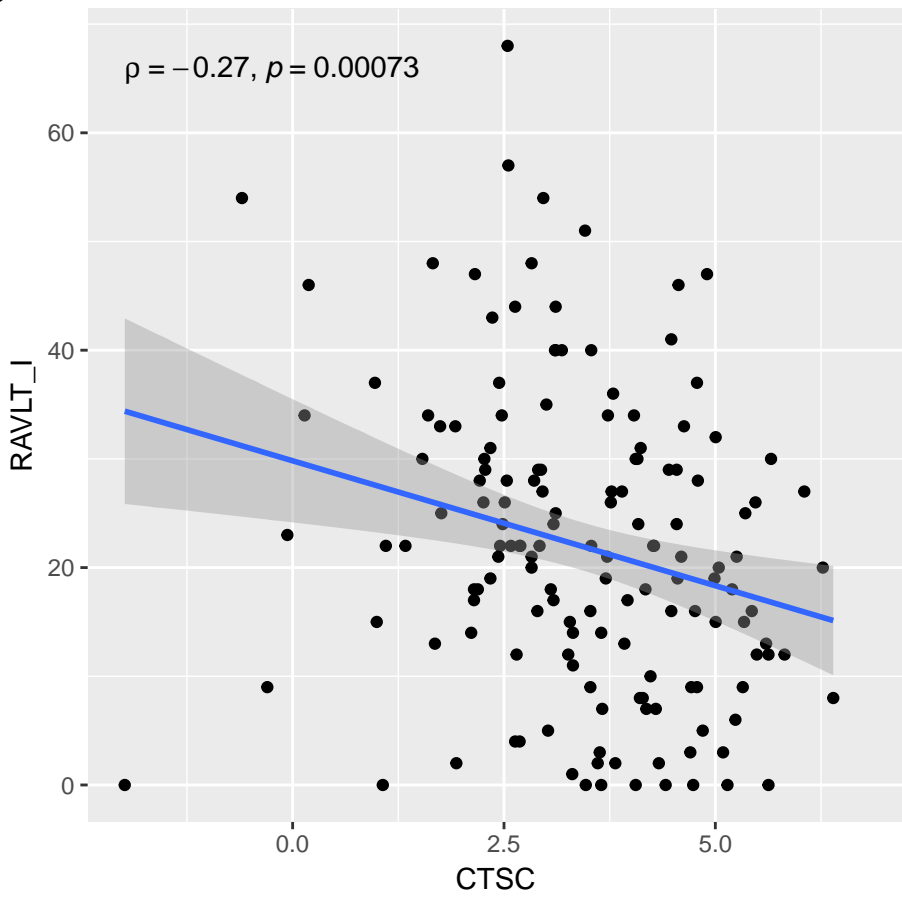**E**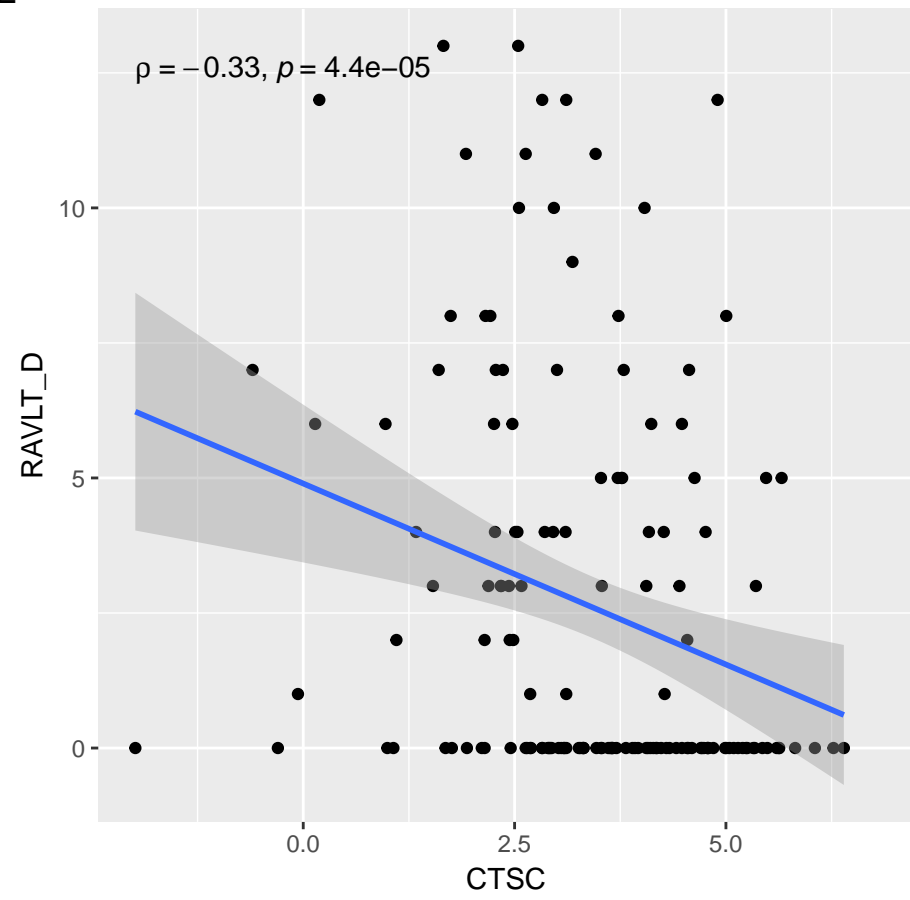**F**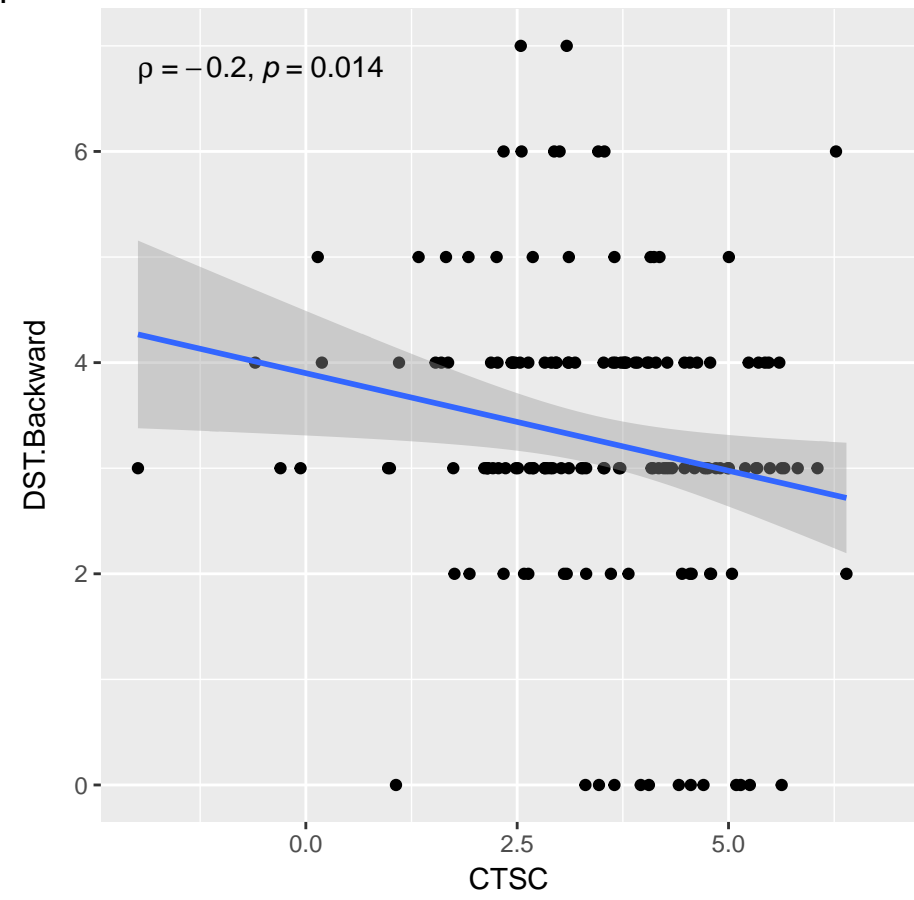**G**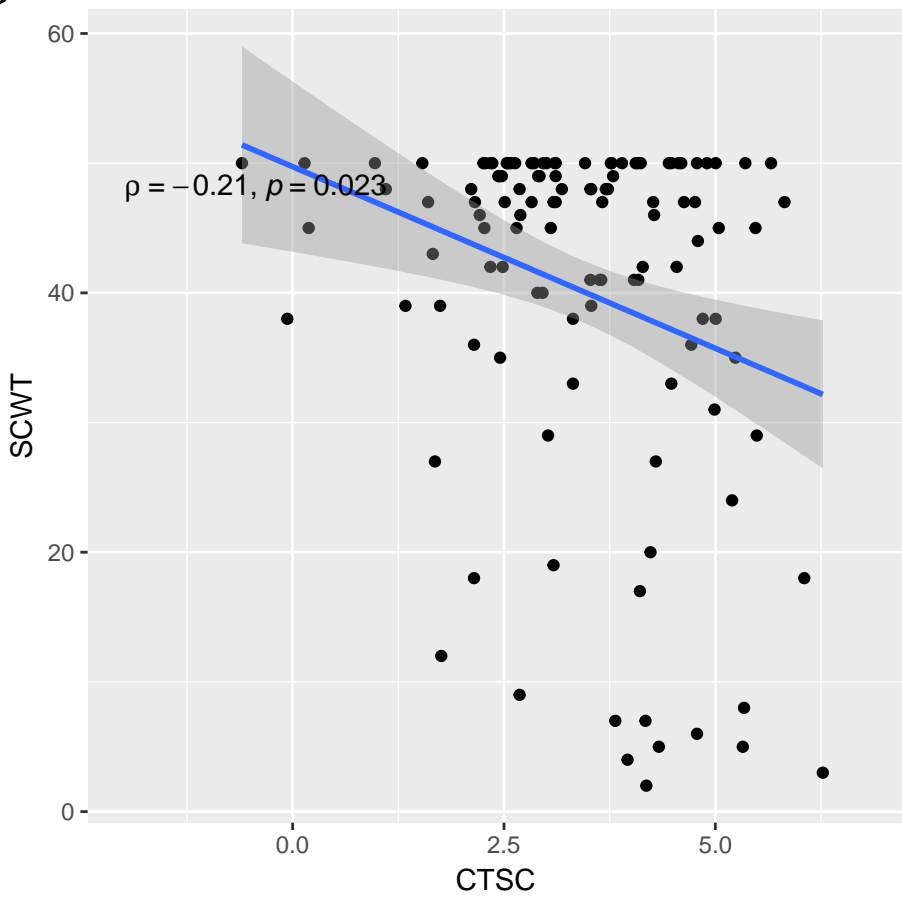**H**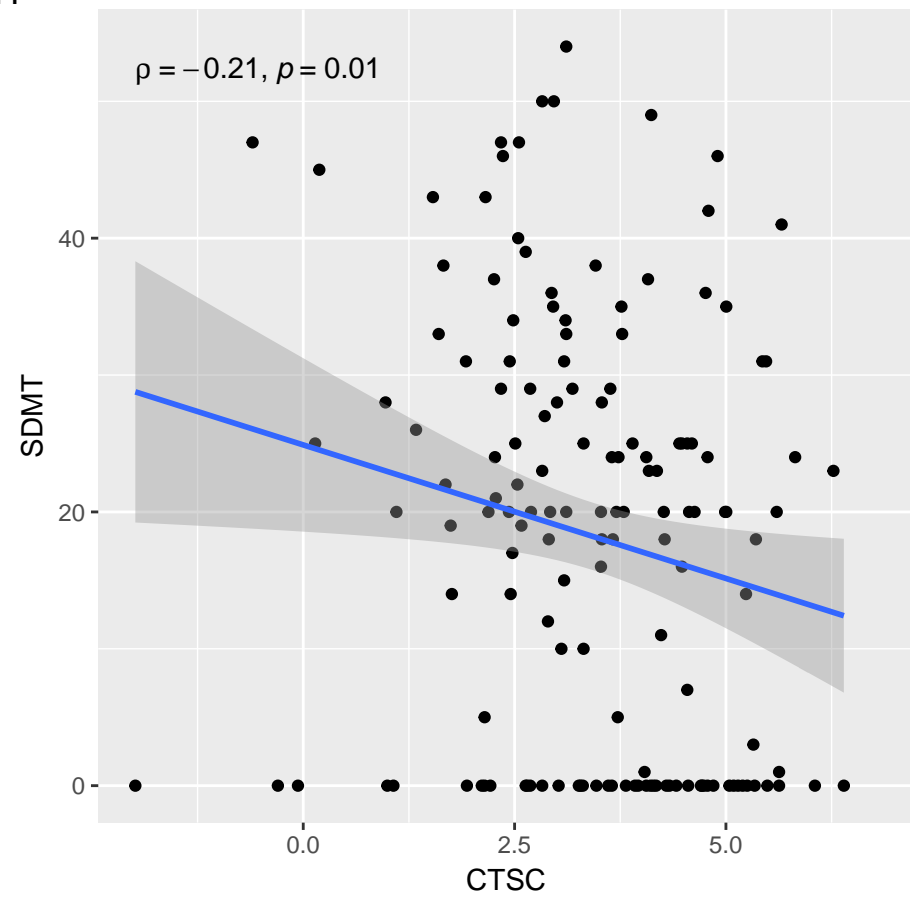**I**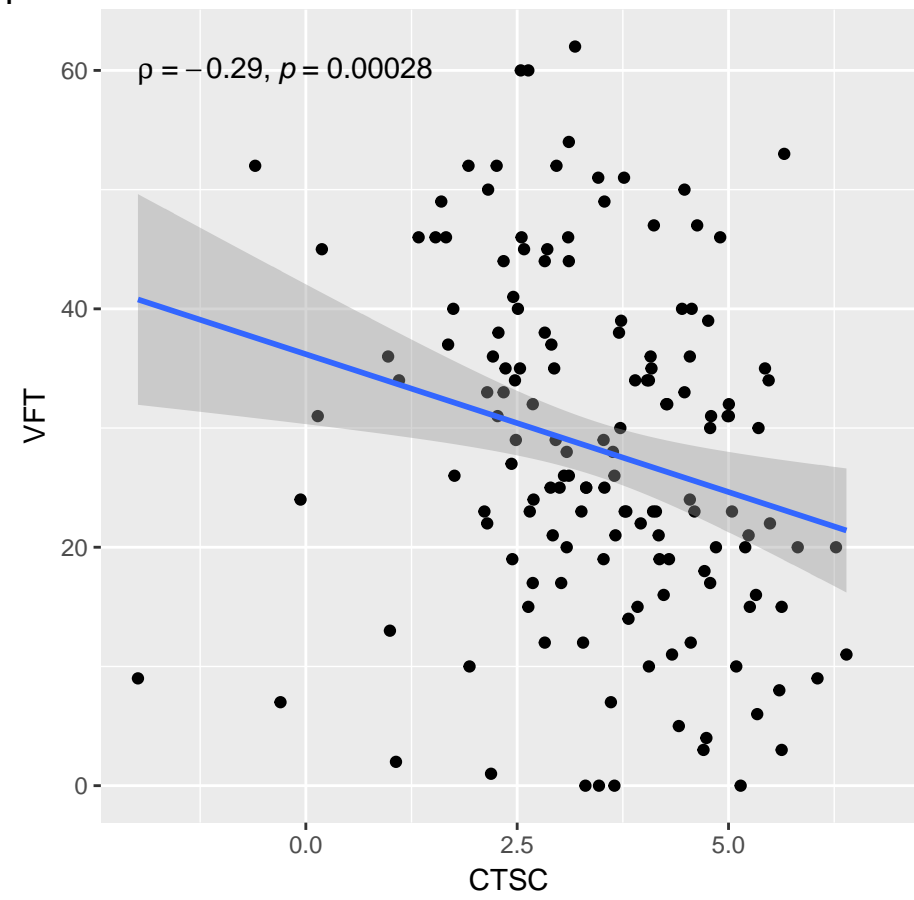**J**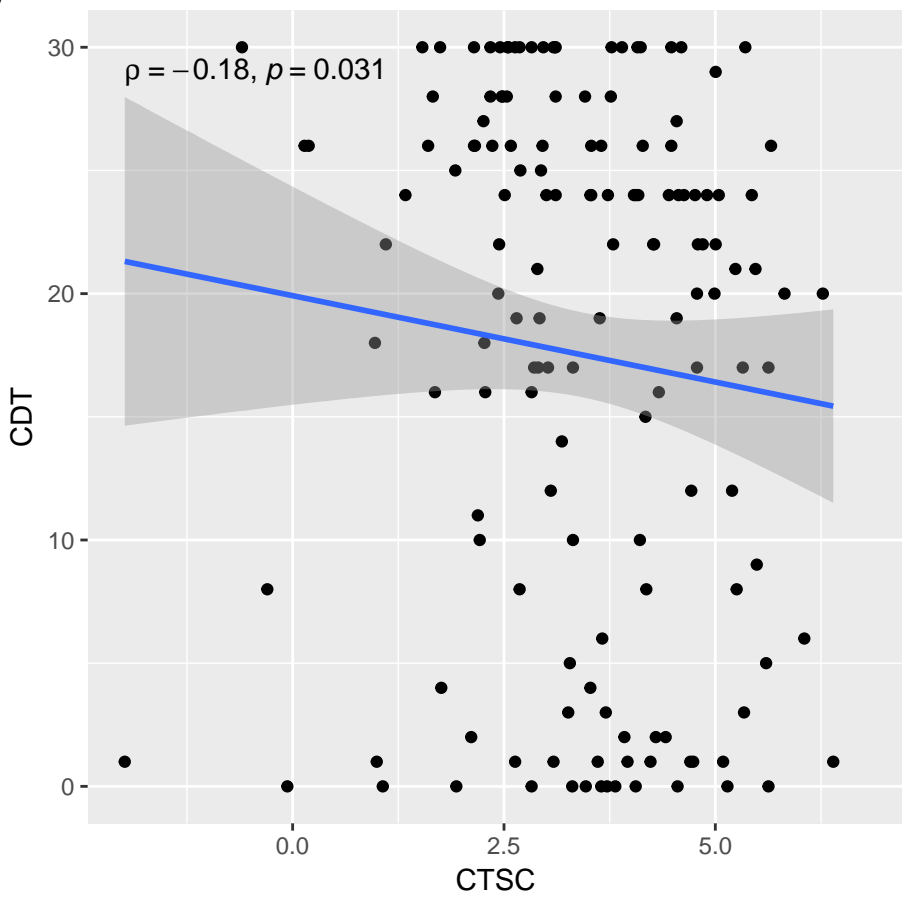

Supplement: Supplementary file 5 — Additional file 5: Fig. S5. Scatter plots of different diagnostic proteins with different cognition tests. [file 13195_2023_1324_MOESM5_ESM.zip › additional Fig 5-CTSC.pdf]

A

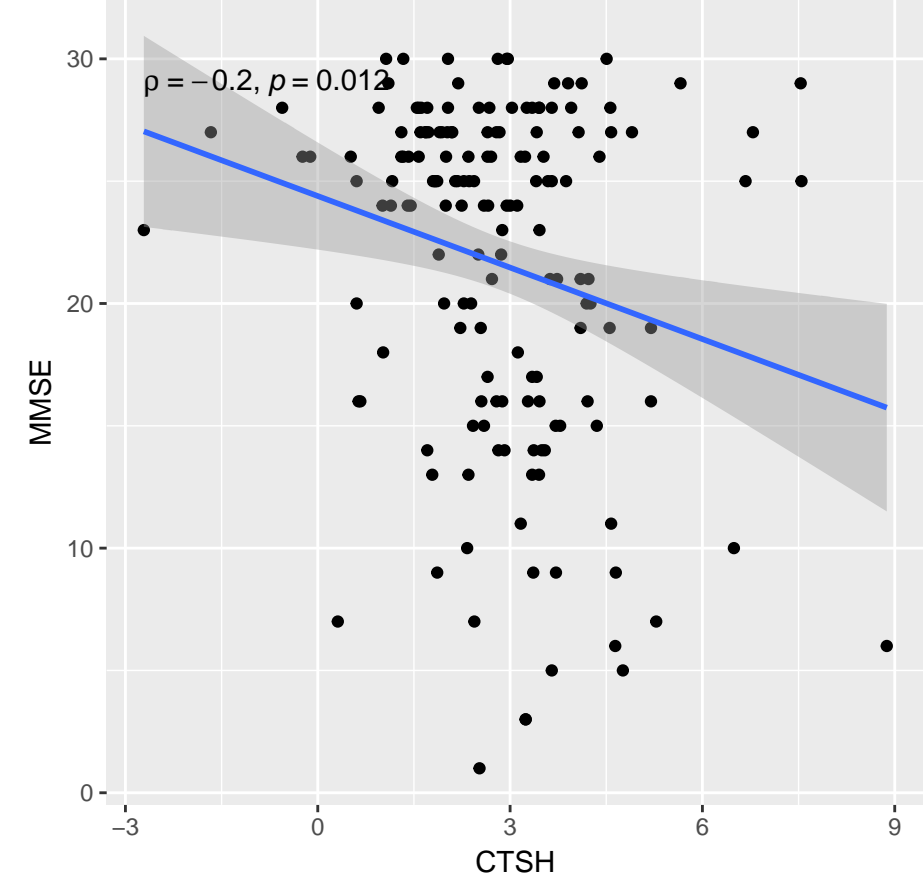

B

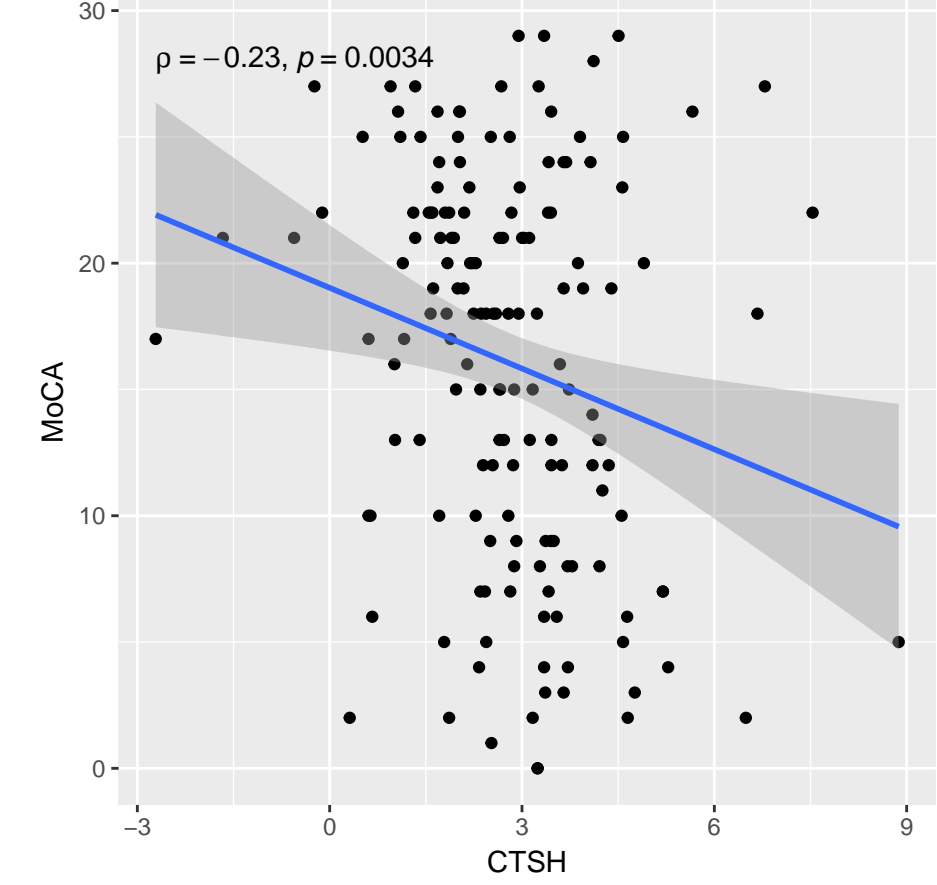

C

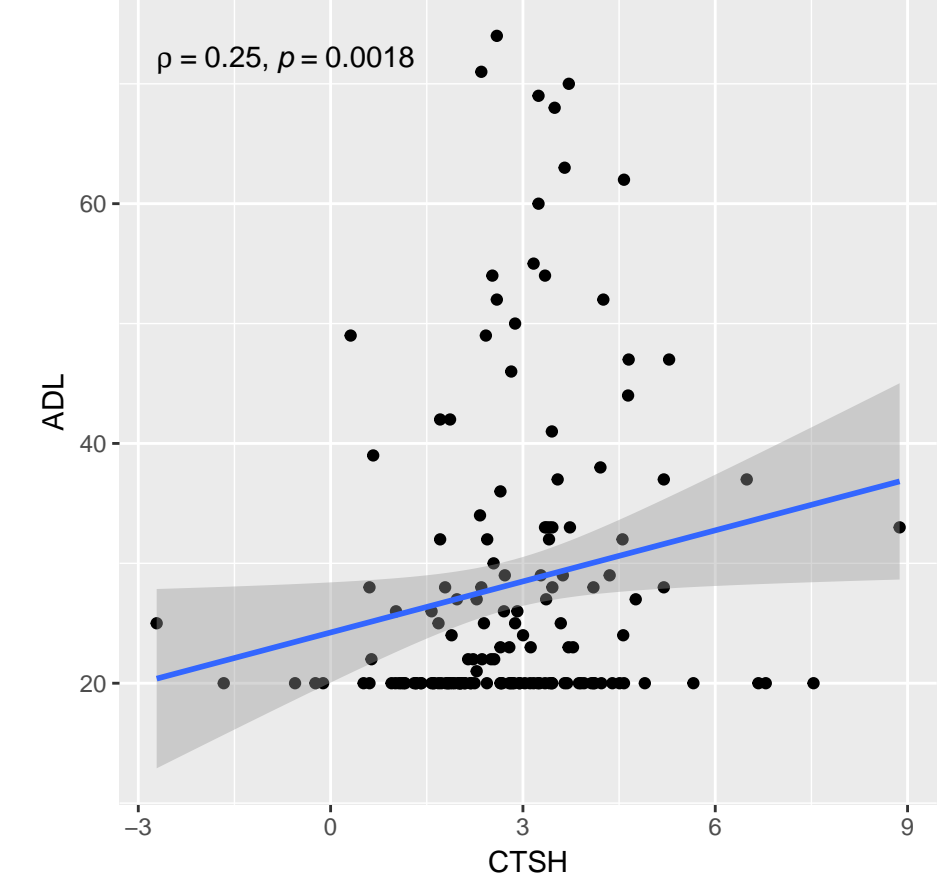

D

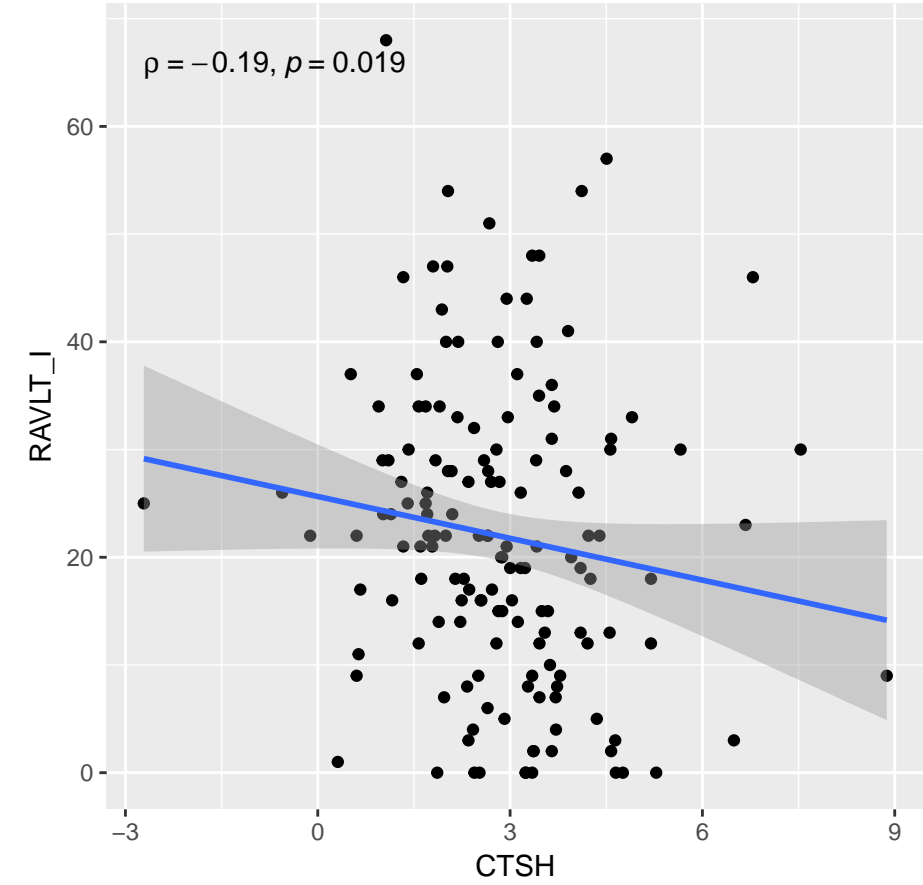

E

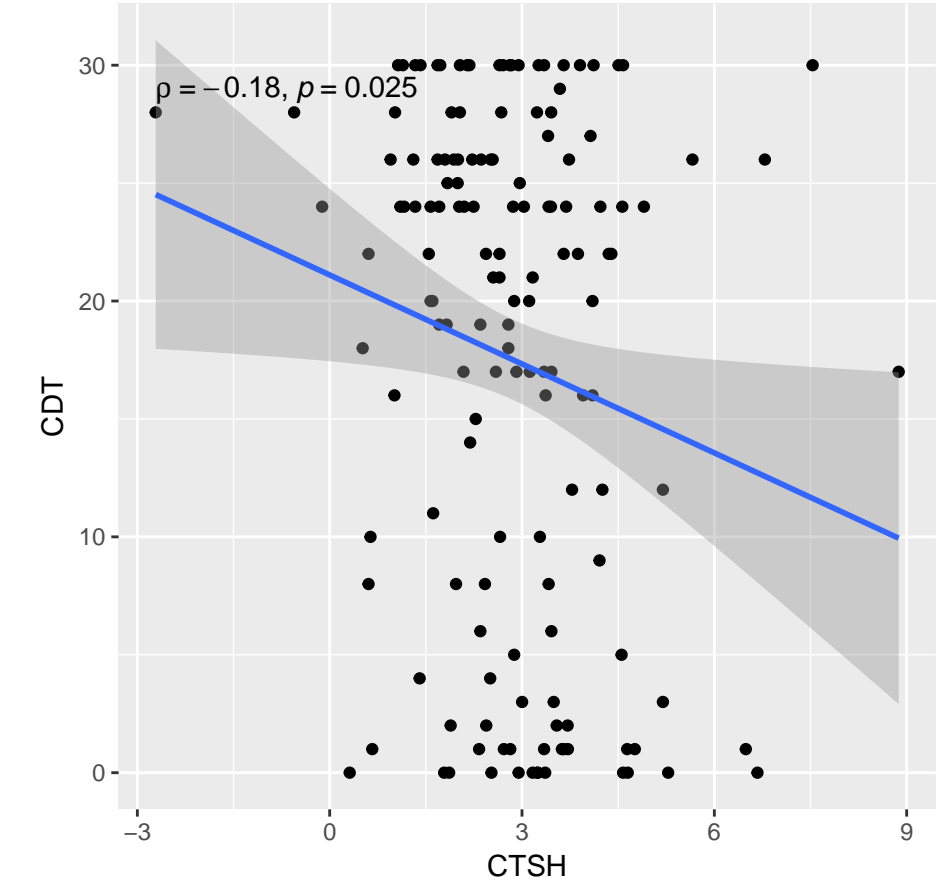

Supplement: Supplementary file 5 — Additional file 5: Fig. S5. Scatter plots of different diagnostic proteins with different cognition tests. [file 13195_2023_1324_MOESM5_ESM.zip › additional Fig 5-CTSH.pdf]

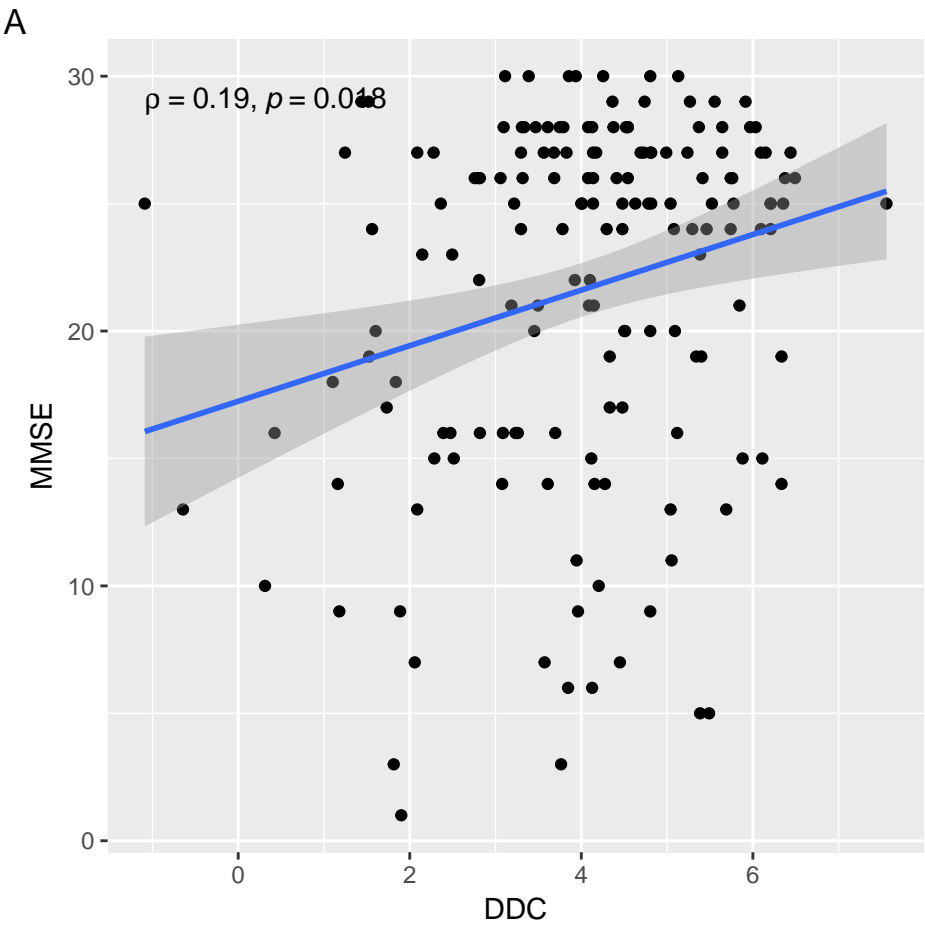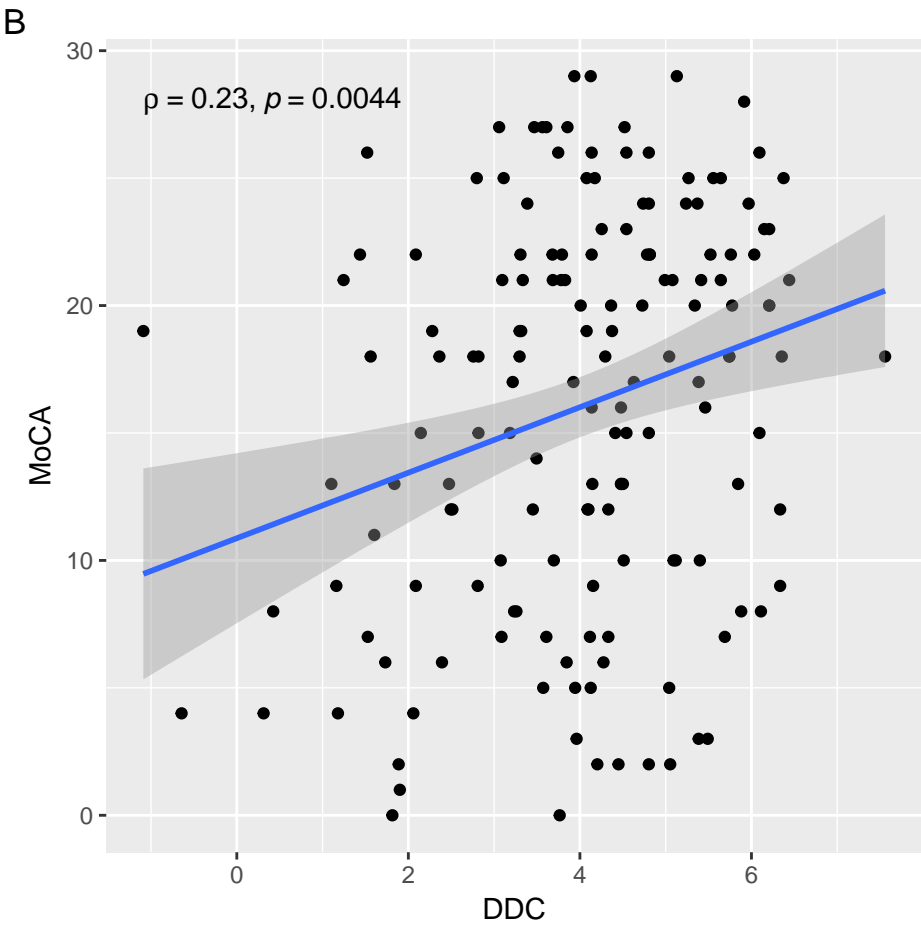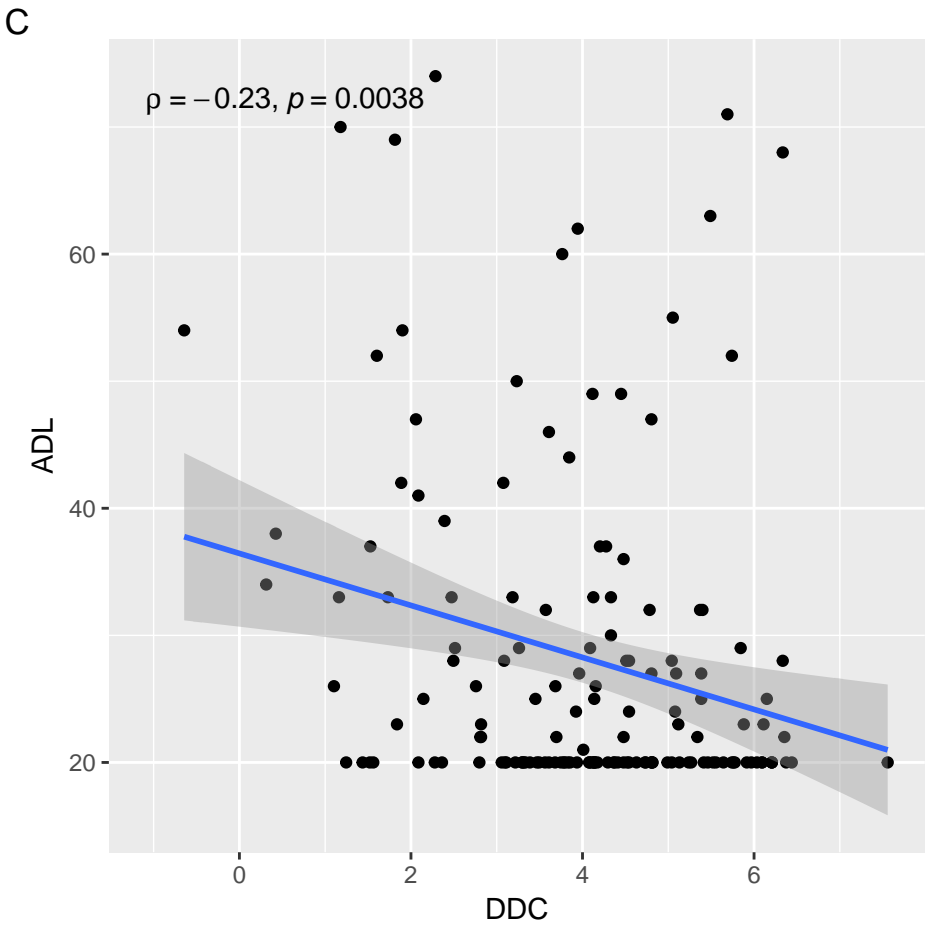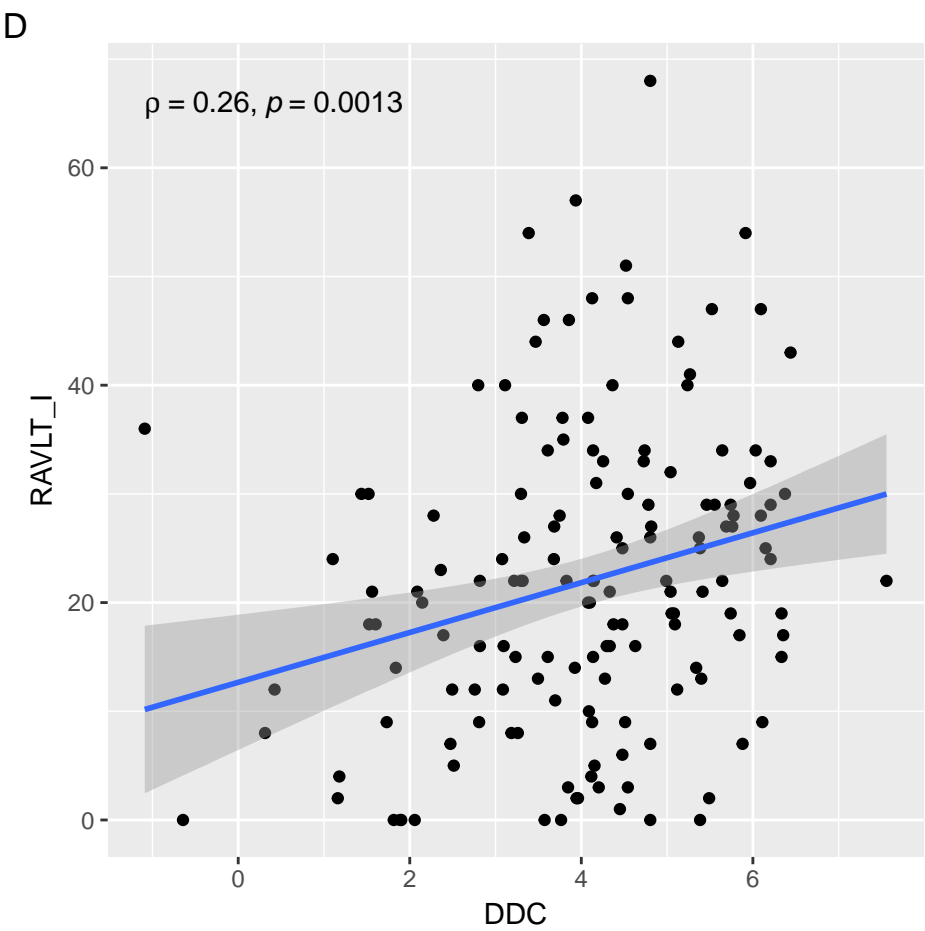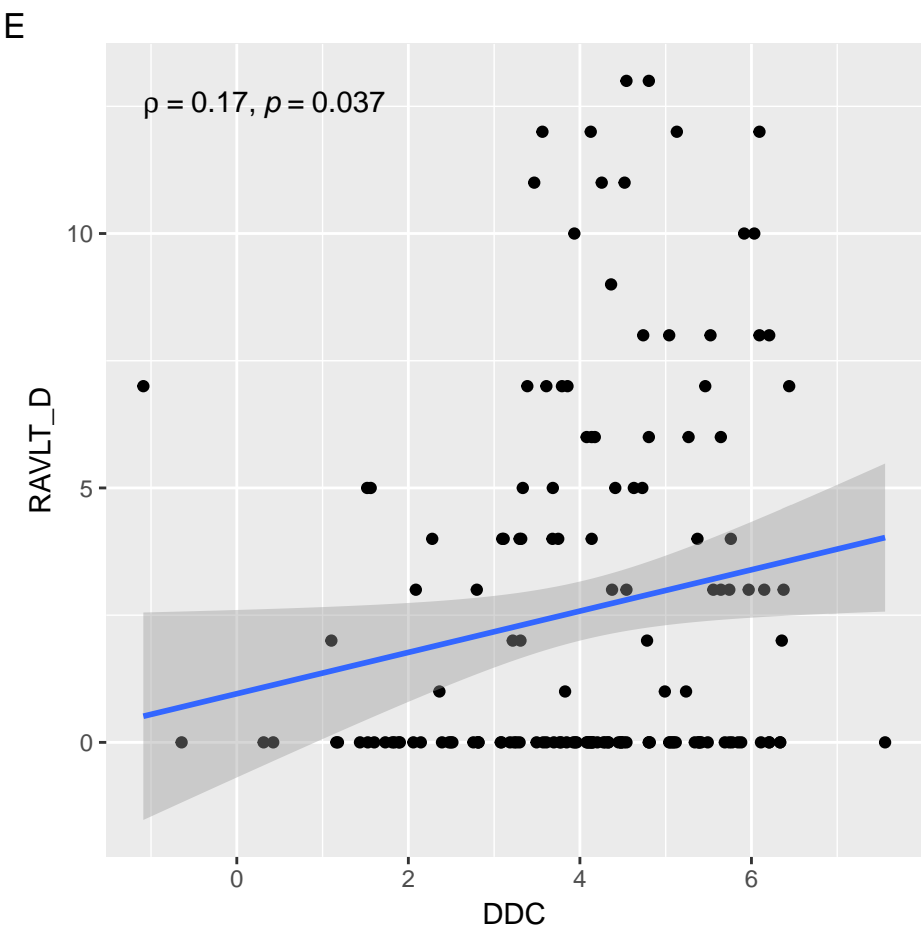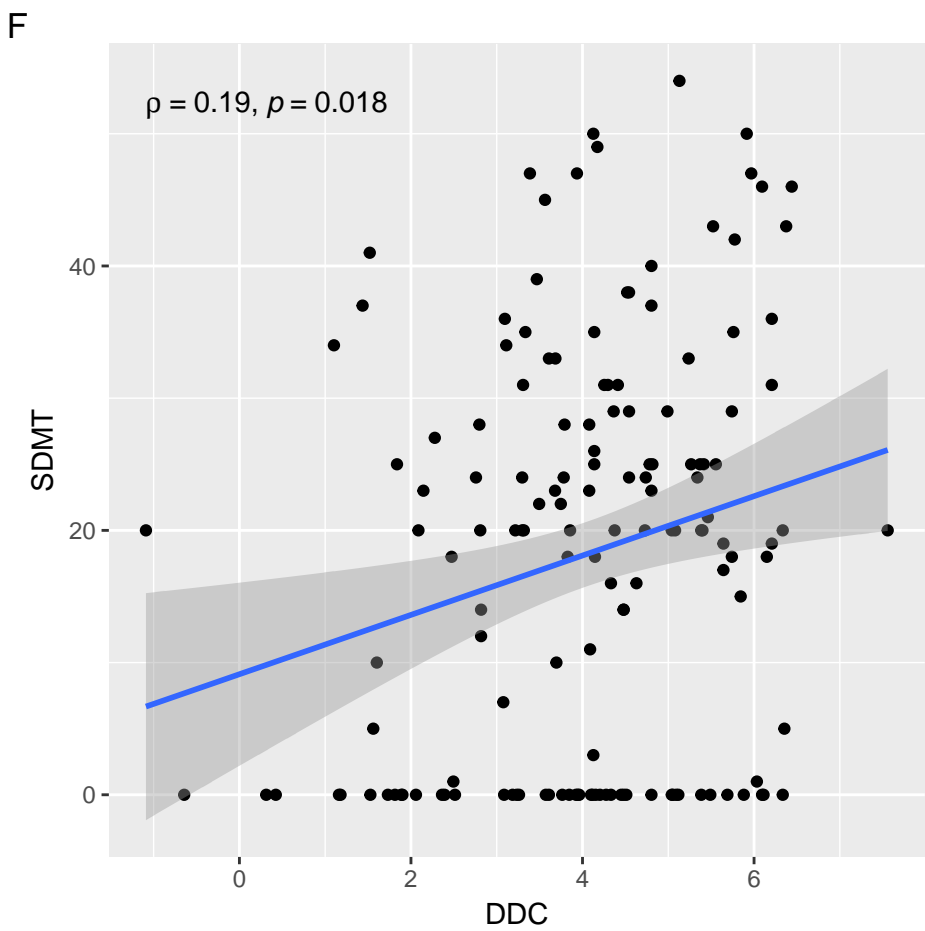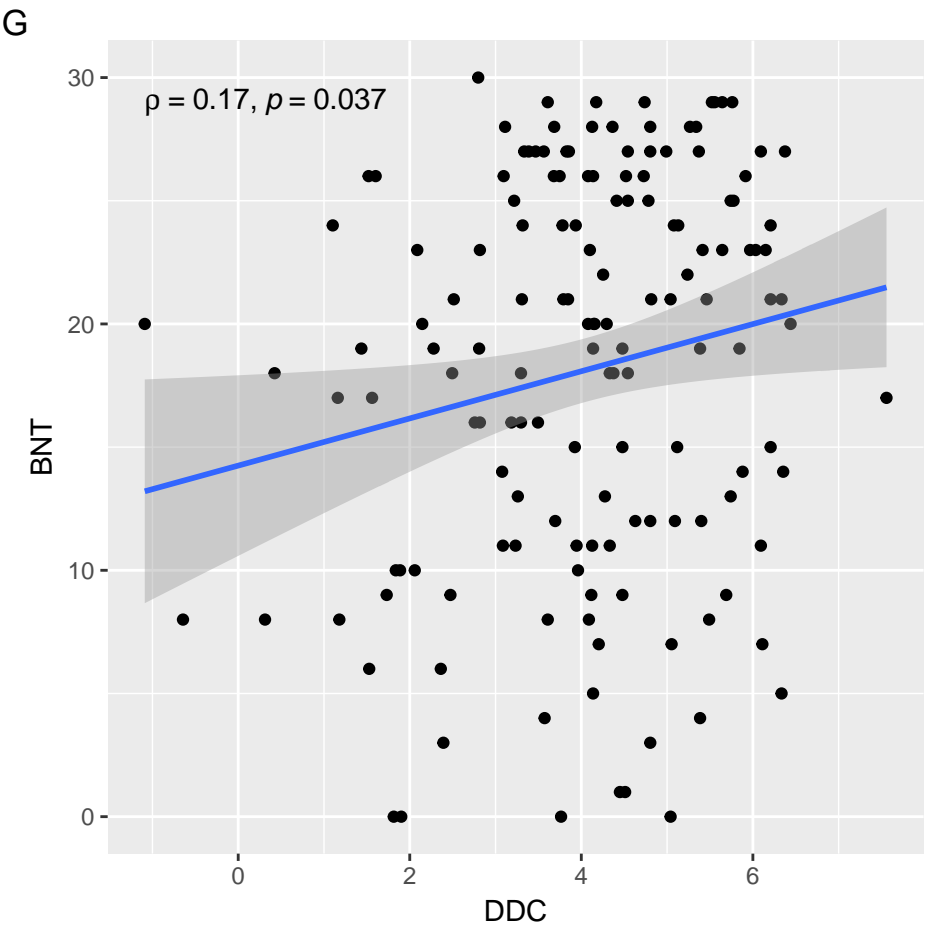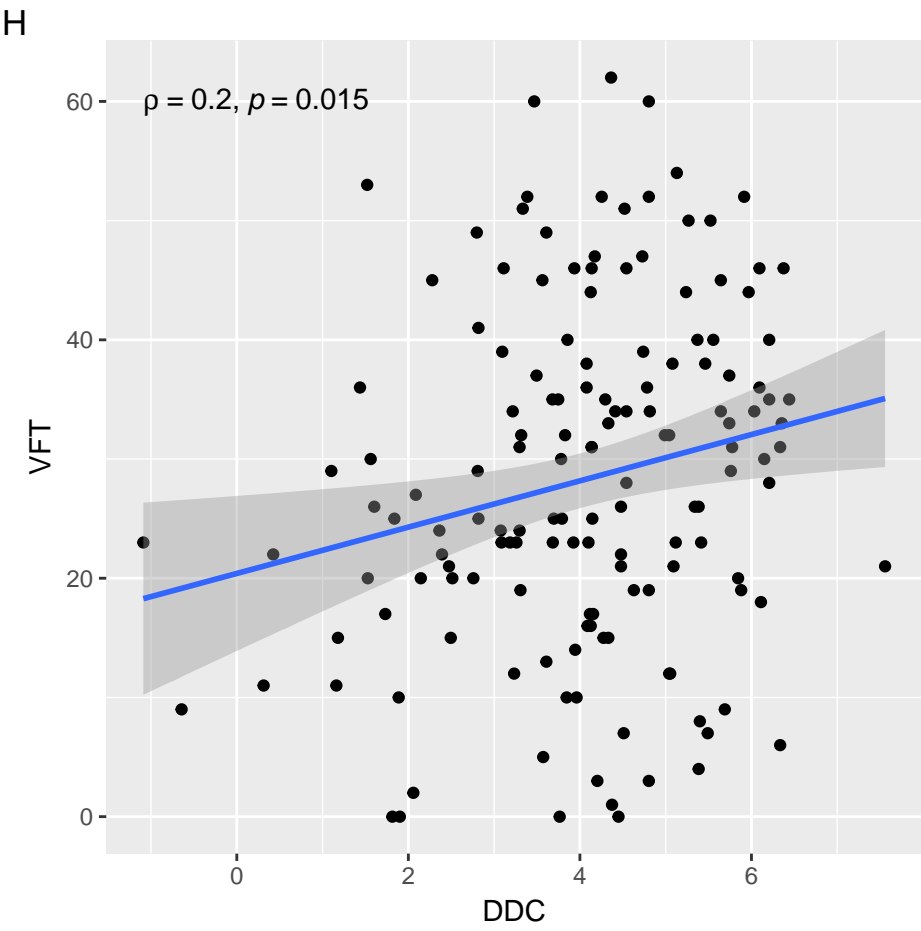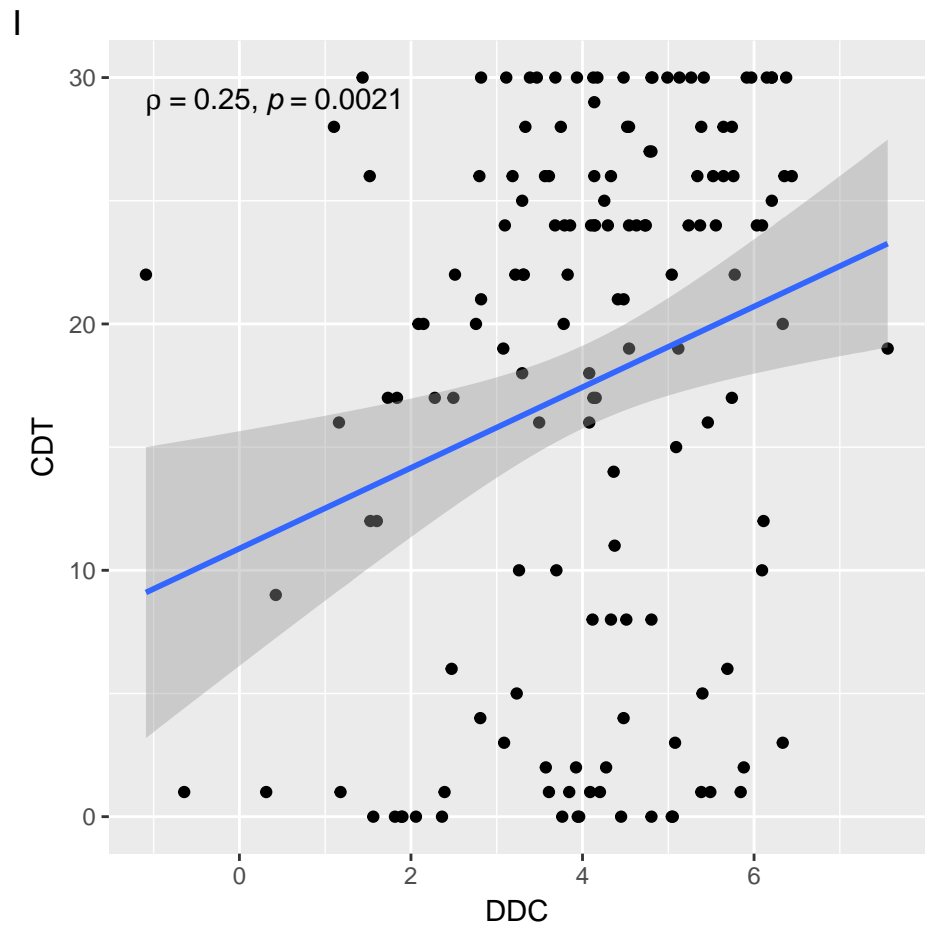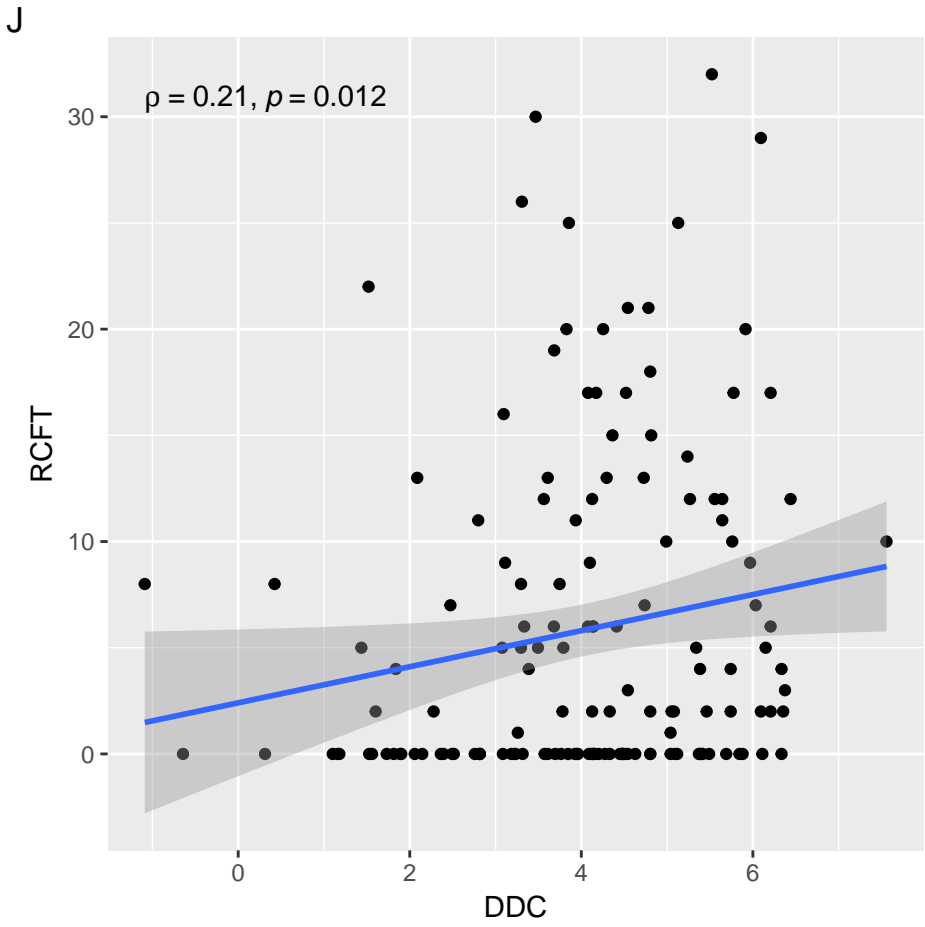

Supplement: Supplementary file 5 — Additional file 5: Fig. S5. Scatter plots of different diagnostic proteins with different cognition tests. [file 13195_2023_1324_MOESM5_ESM.zip › additional Fig 5-DDC.pdf]

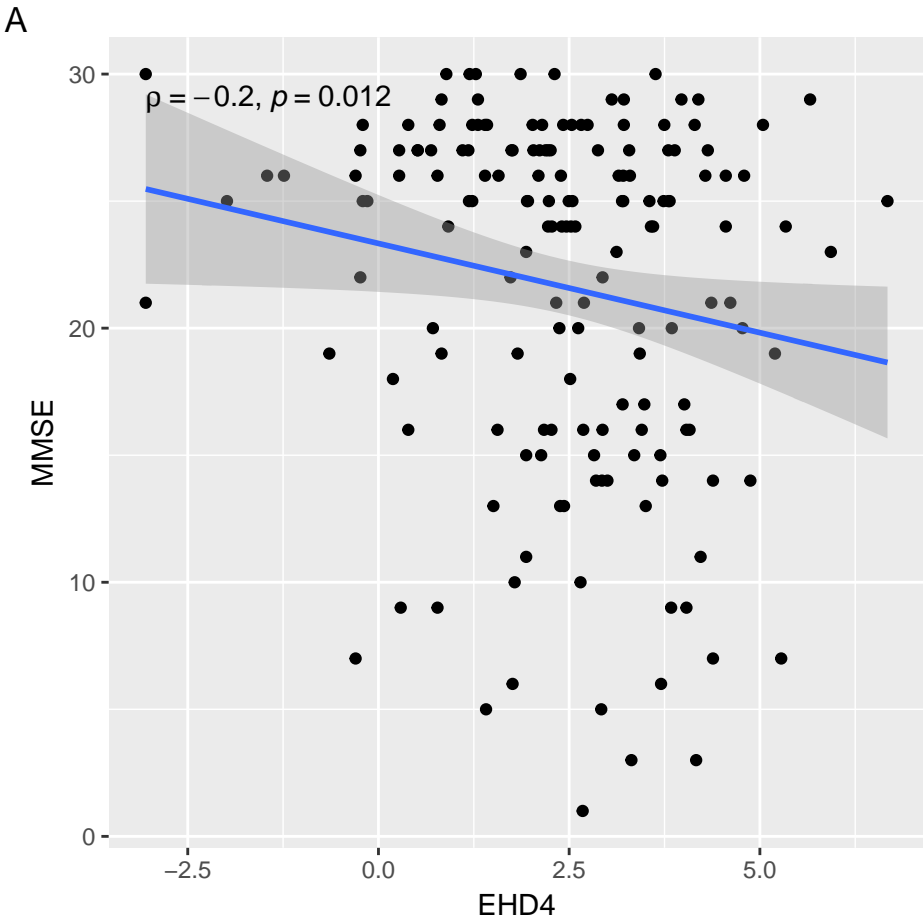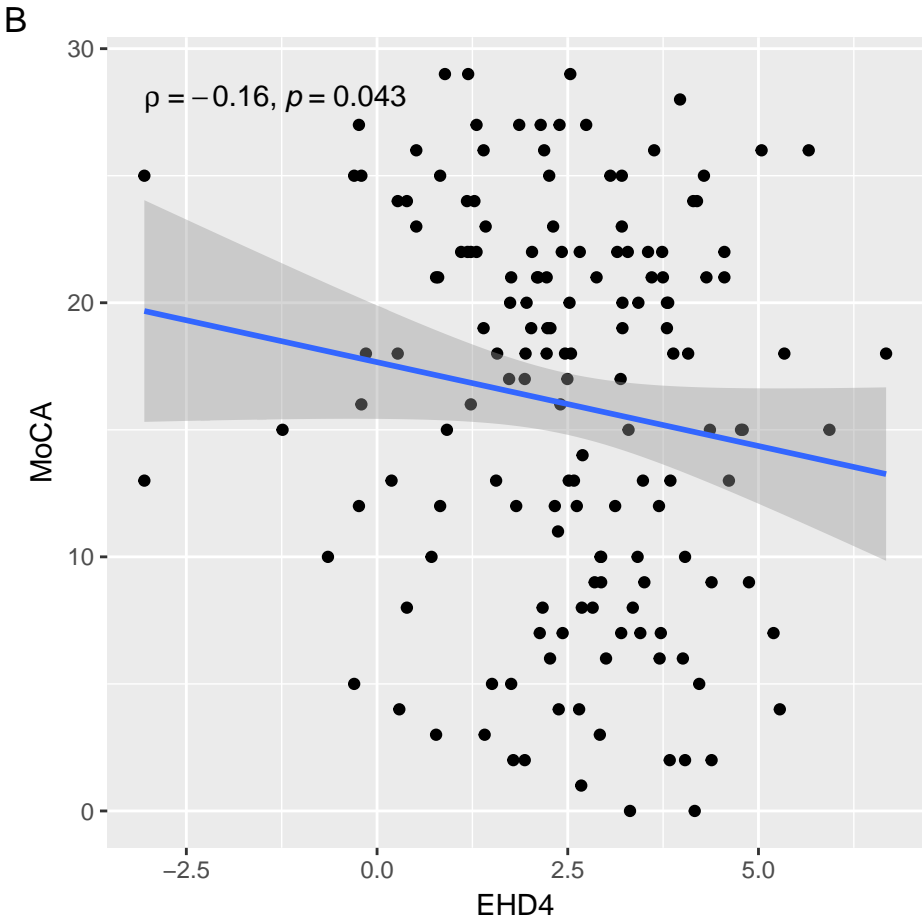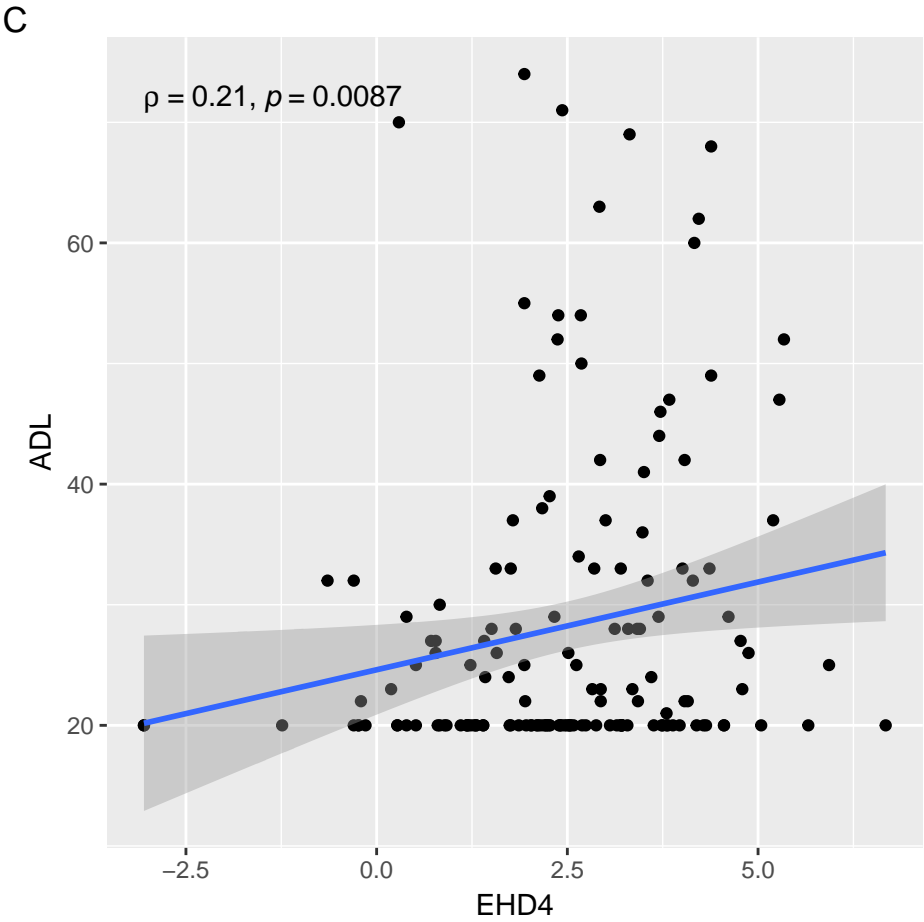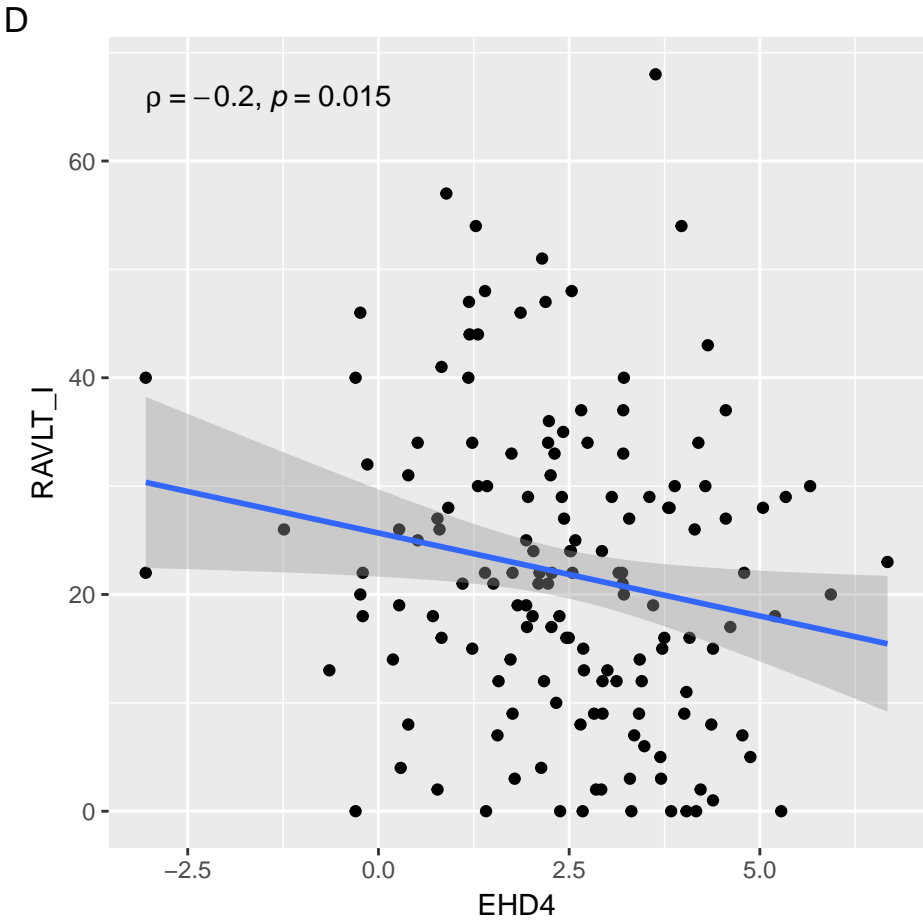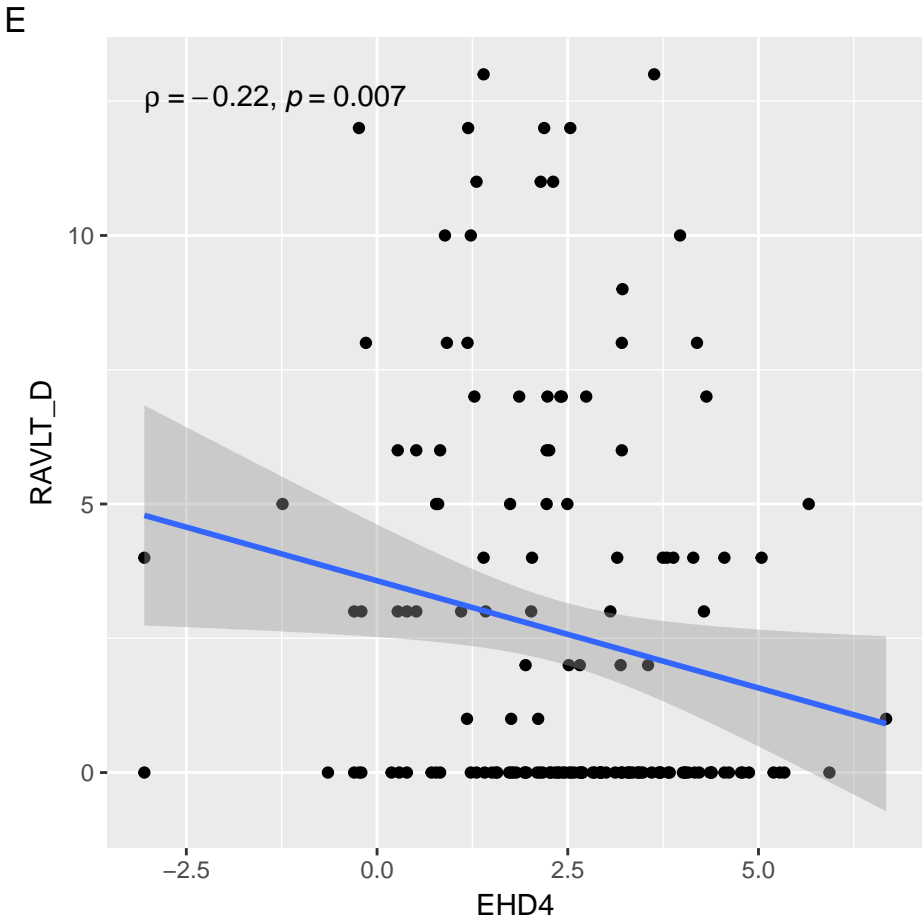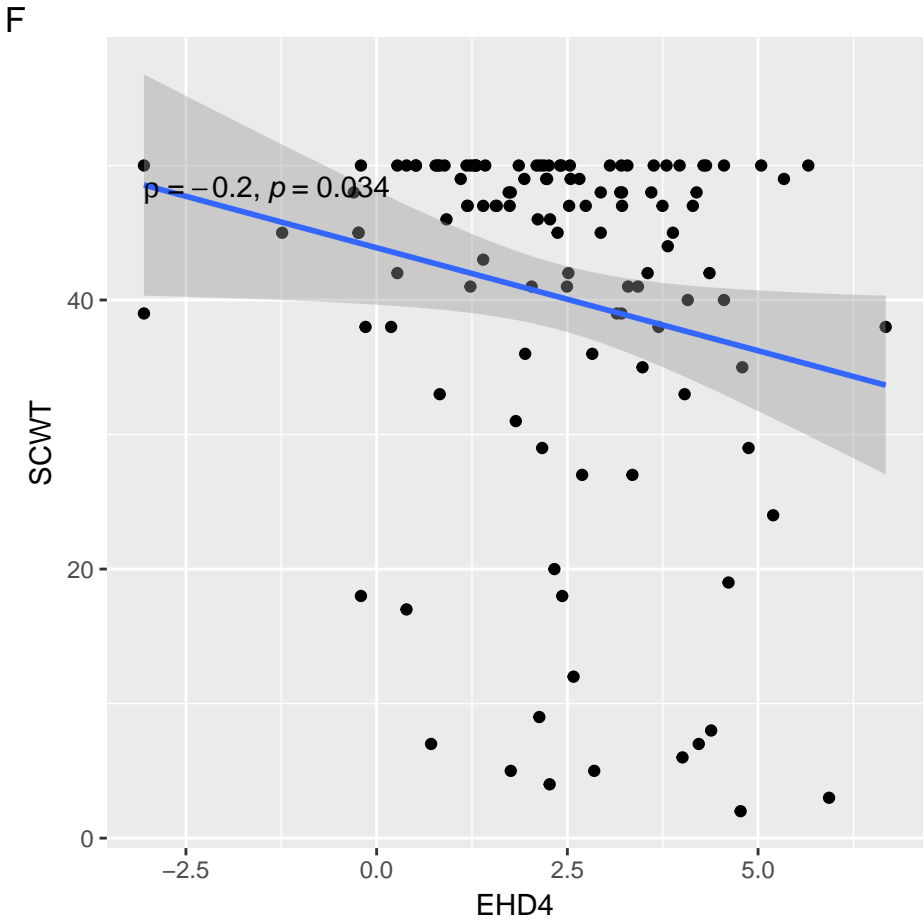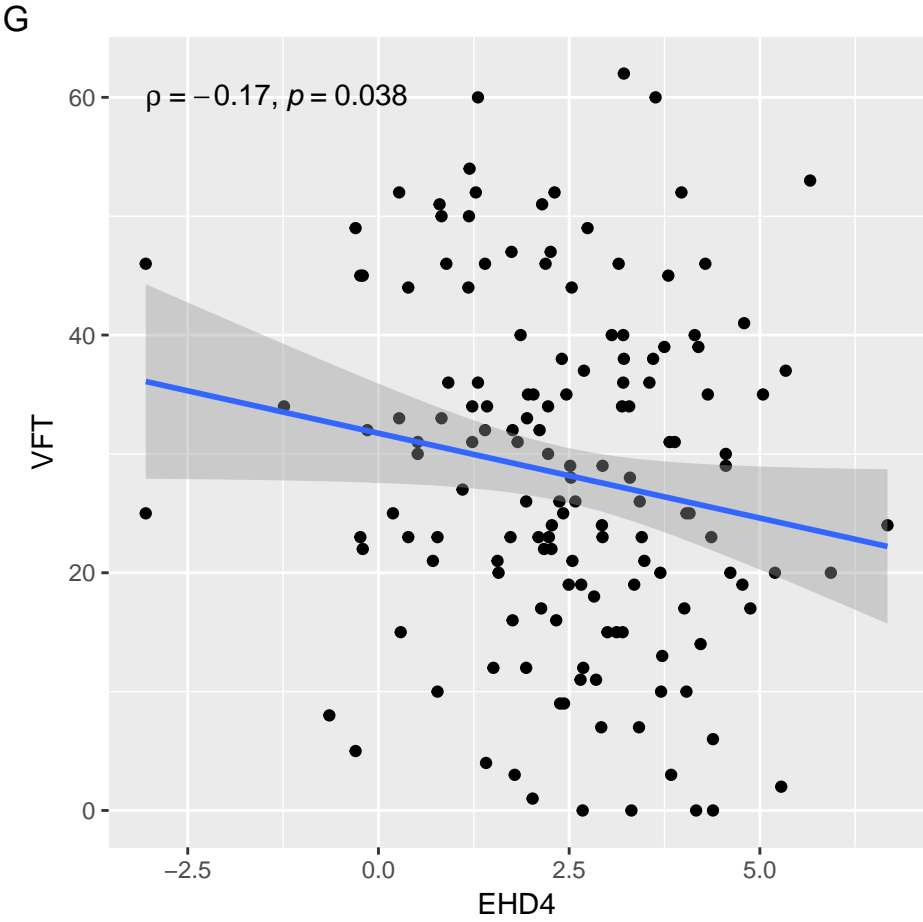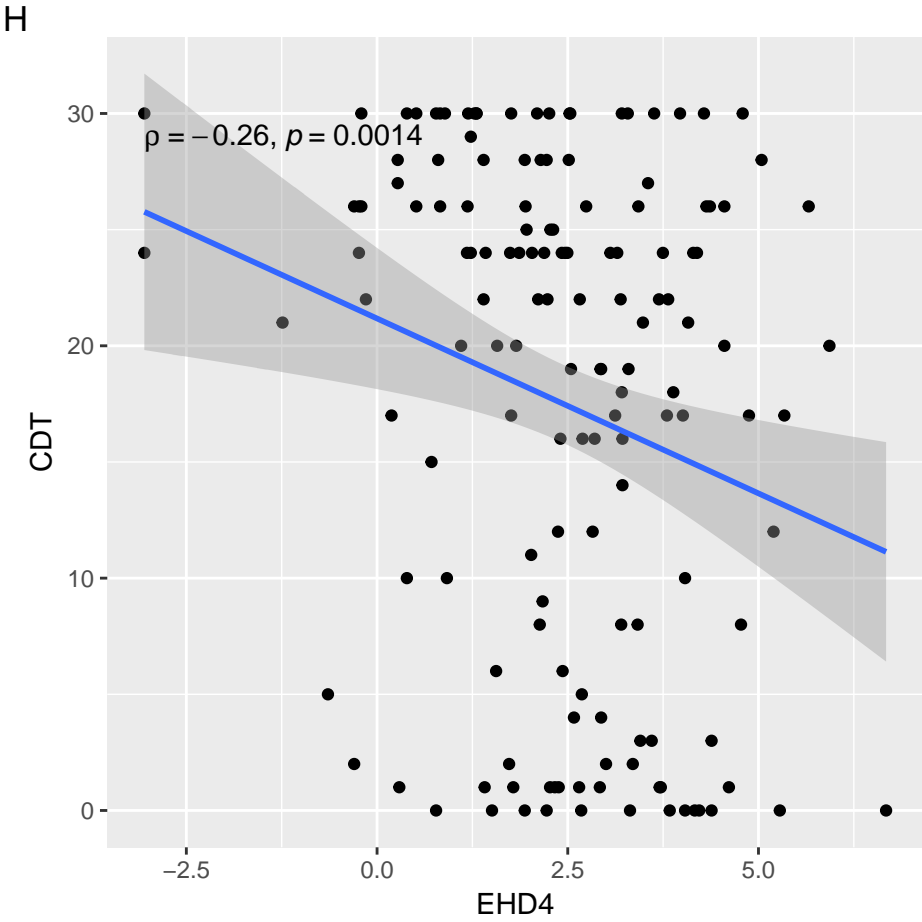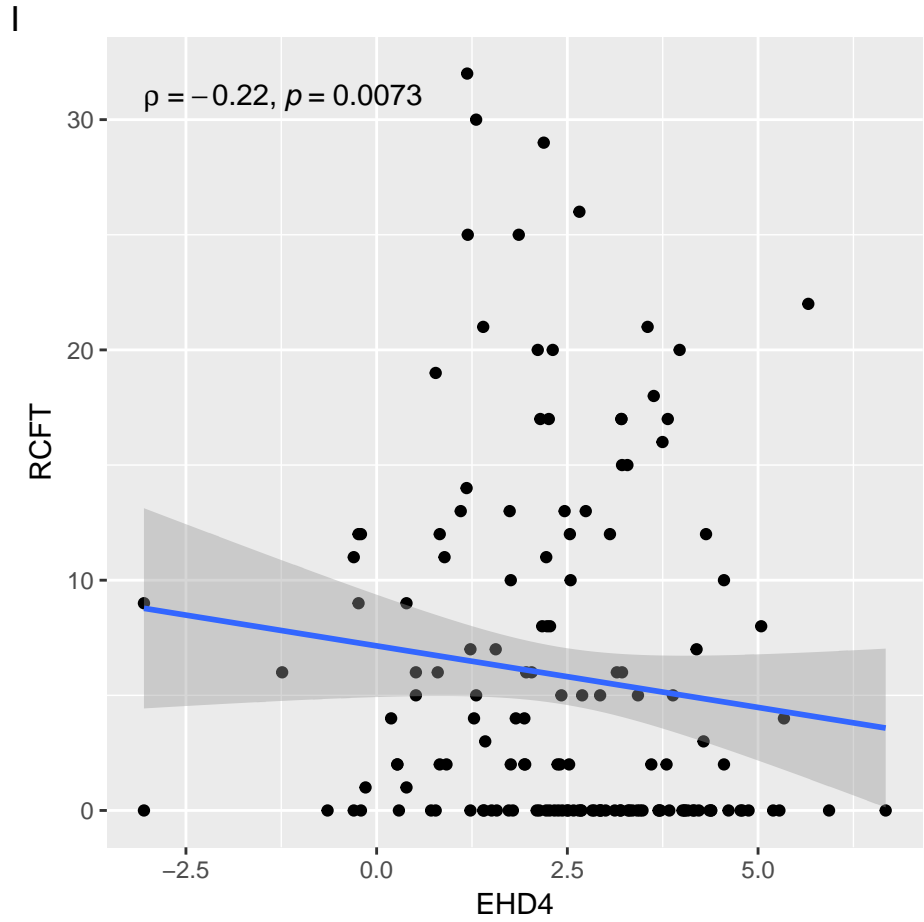

Supplement: Supplementary file 5 — Additional file 5: Fig. S5. Scatter plots of different diagnostic proteins with different cognition tests. [file 13195_2023_1324_MOESM5_ESM.zip › additional Fig 5-EHD4.pdf]

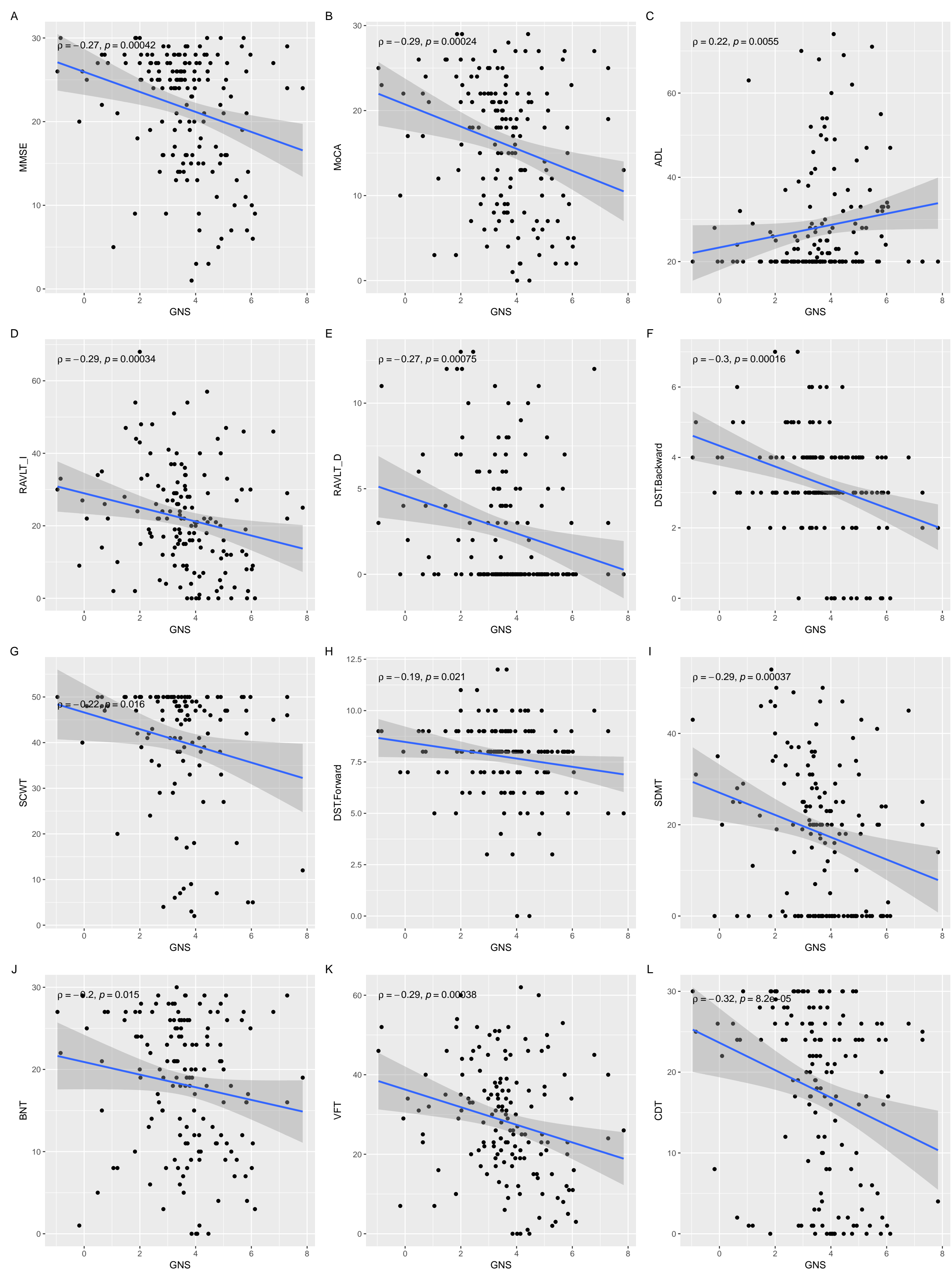

Supplement: Supplementary file 5 — Additional file 5: Fig. S5. Scatter plots of different diagnostic proteins with different cognition tests. [file 13195_2023_1324_MOESM5_ESM.zip › additional Fig 5-GNS.pdf]

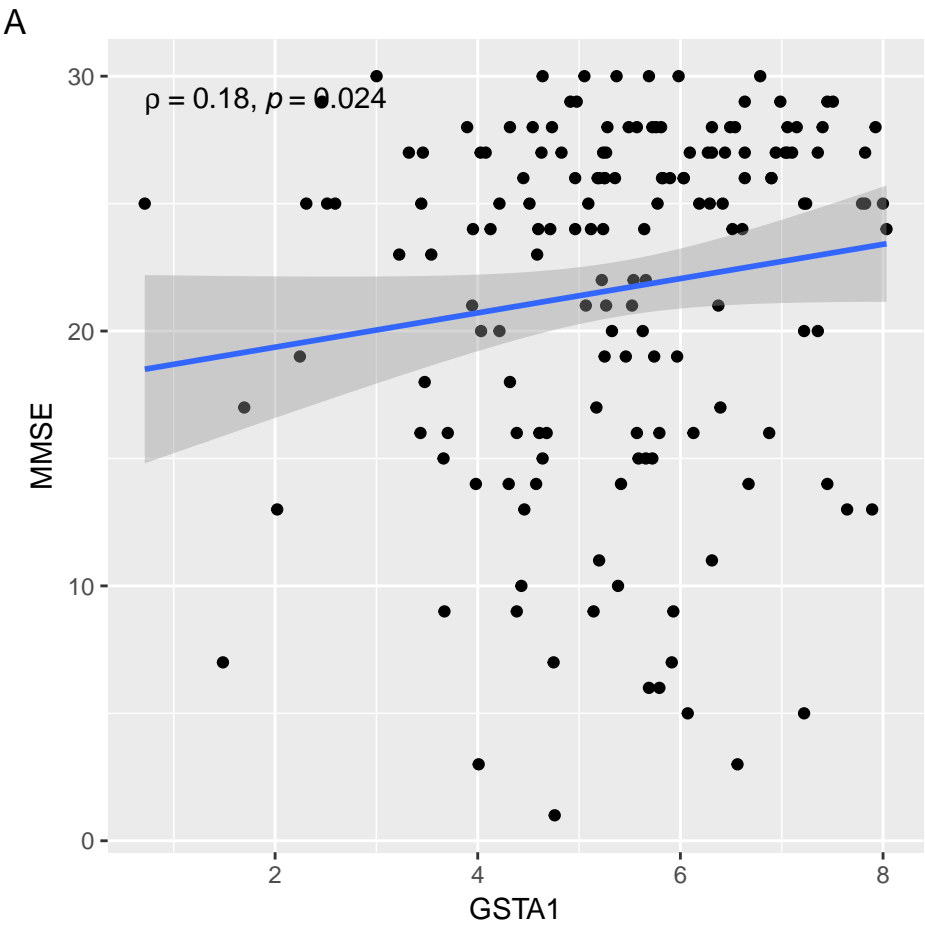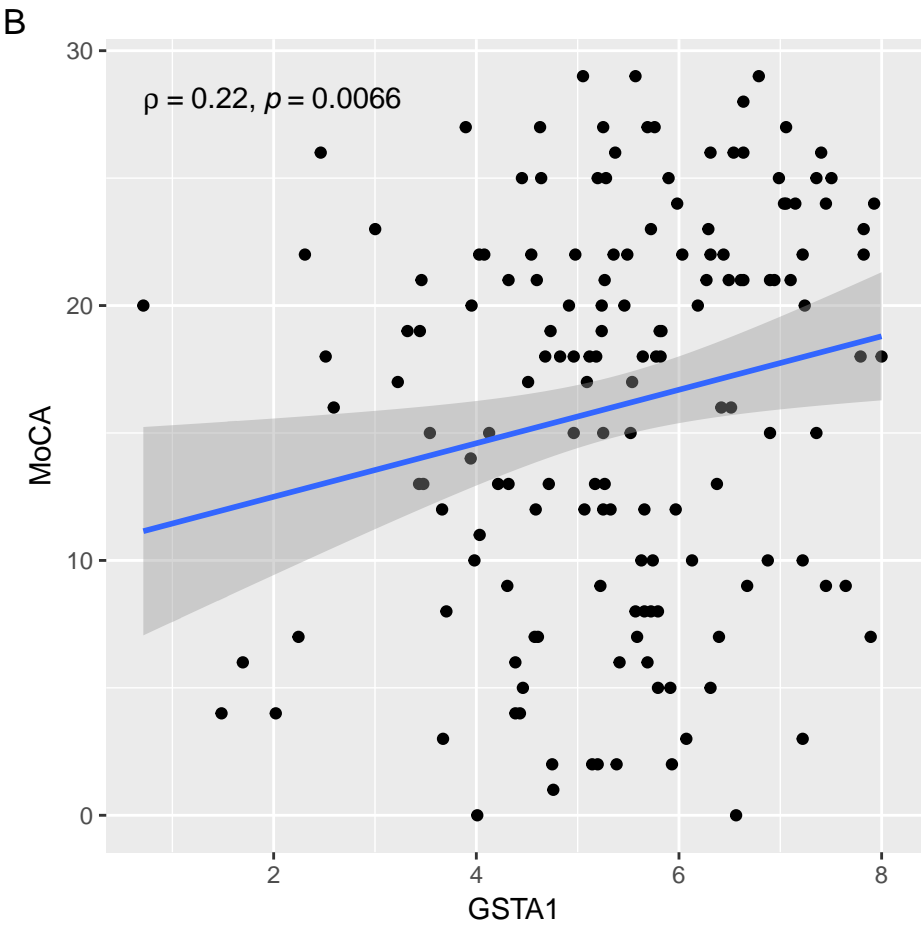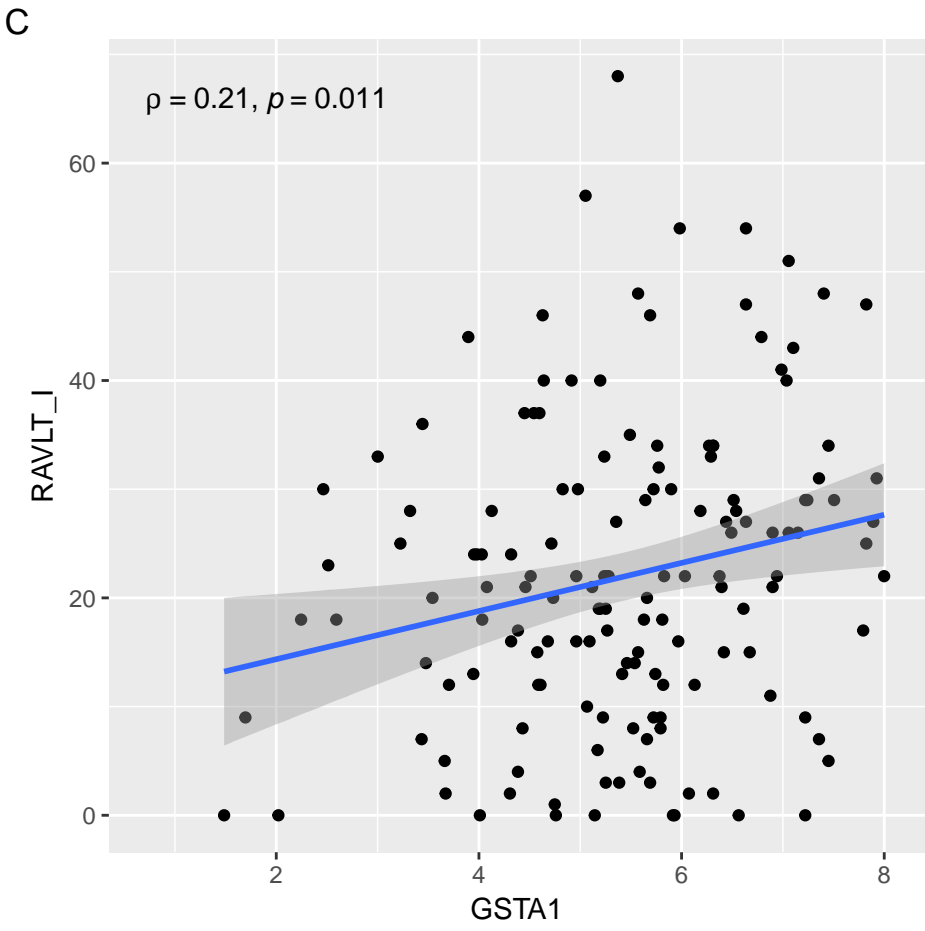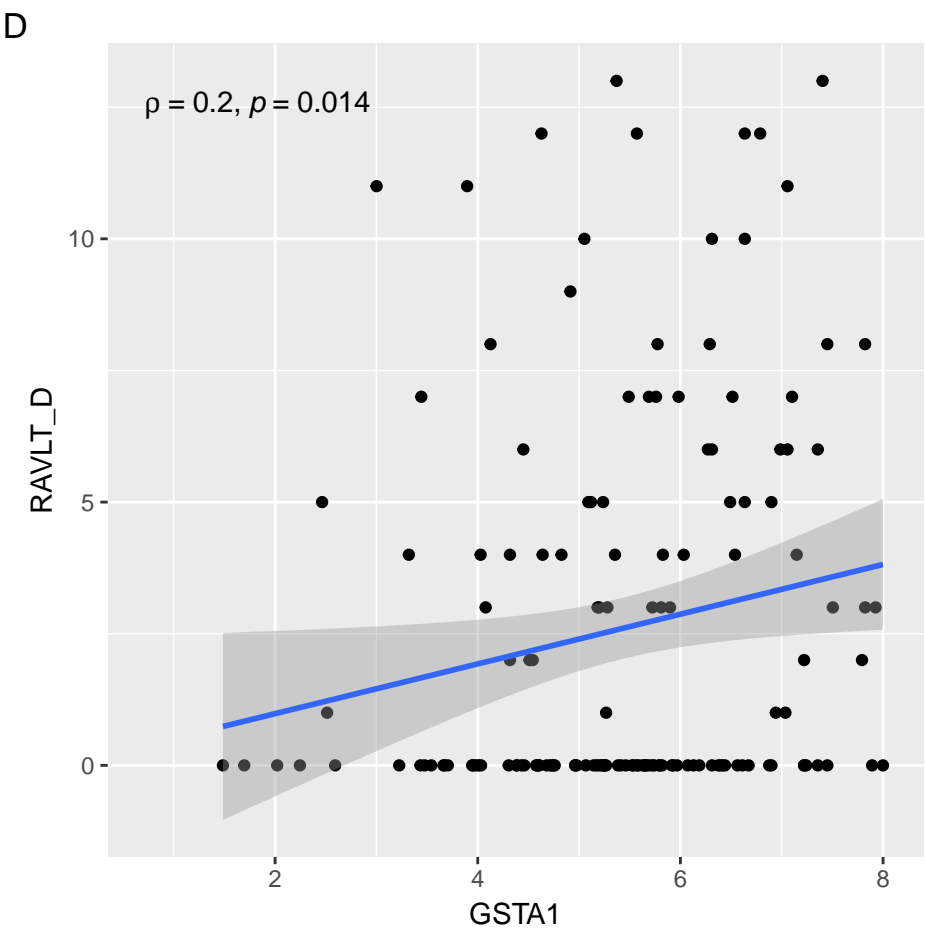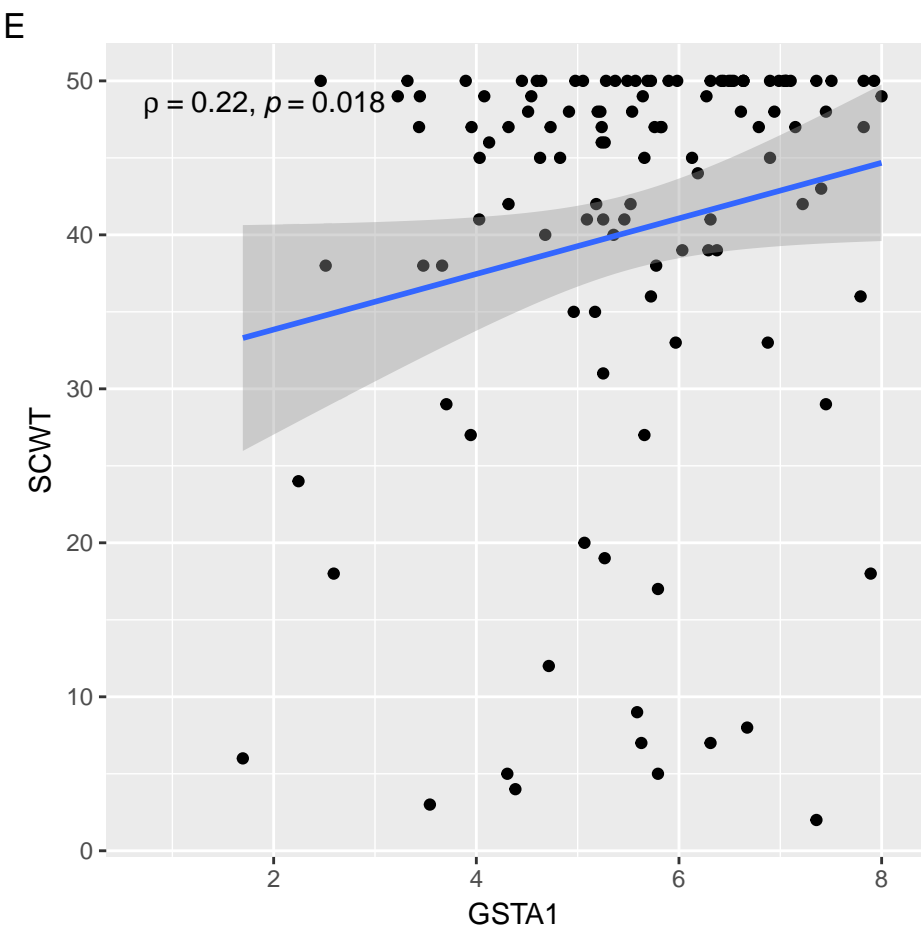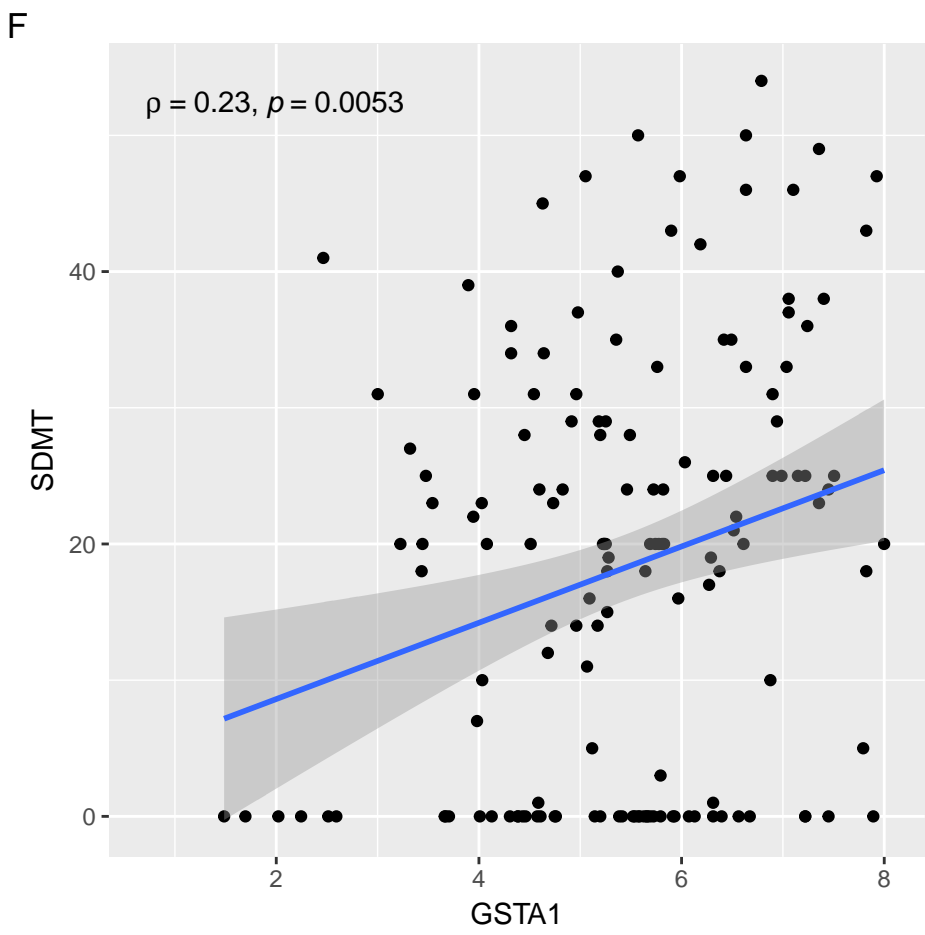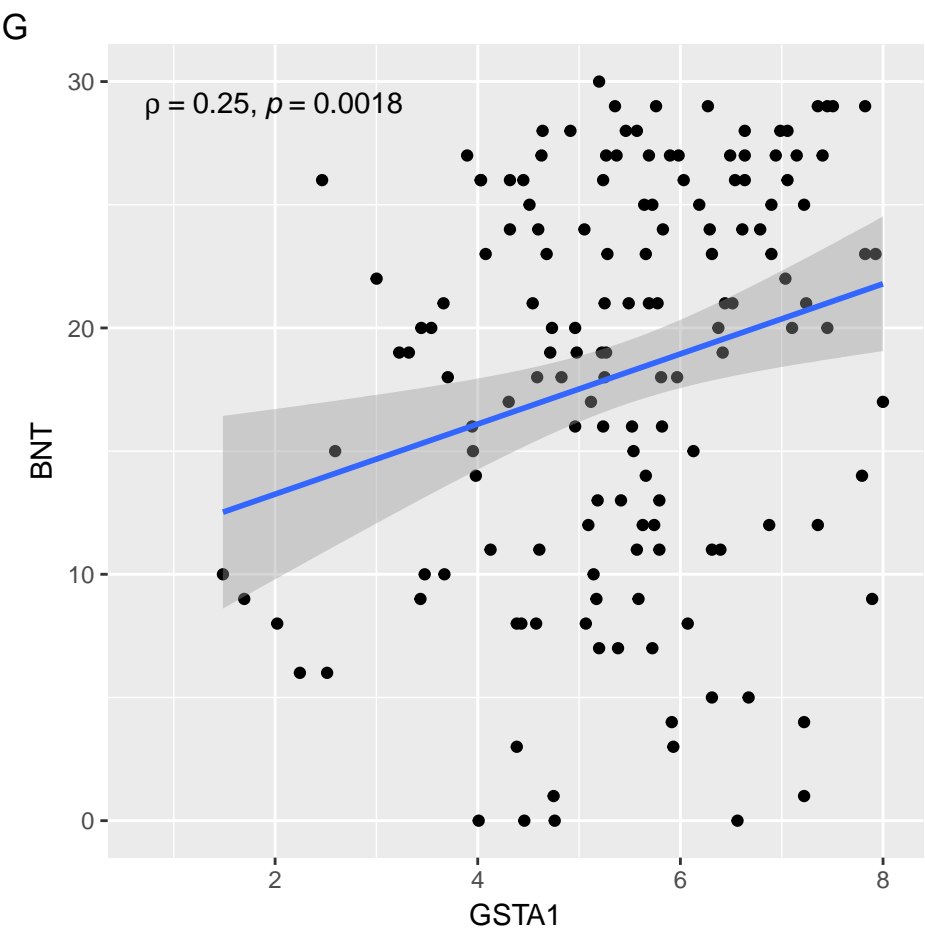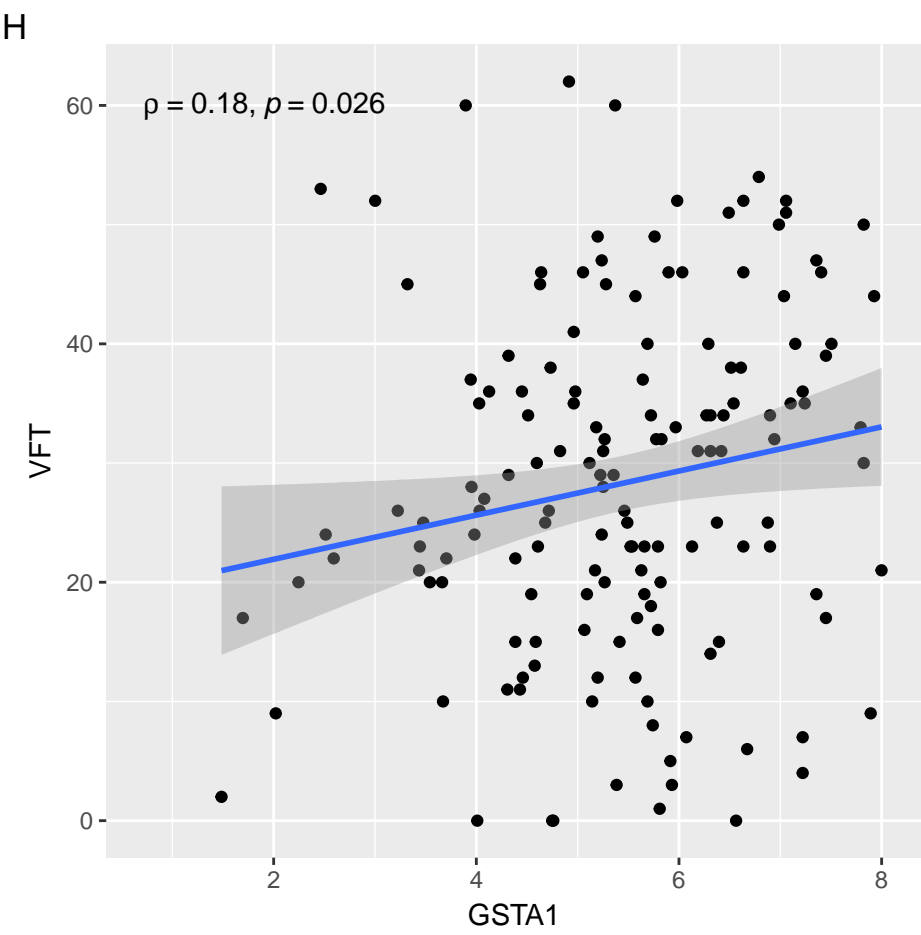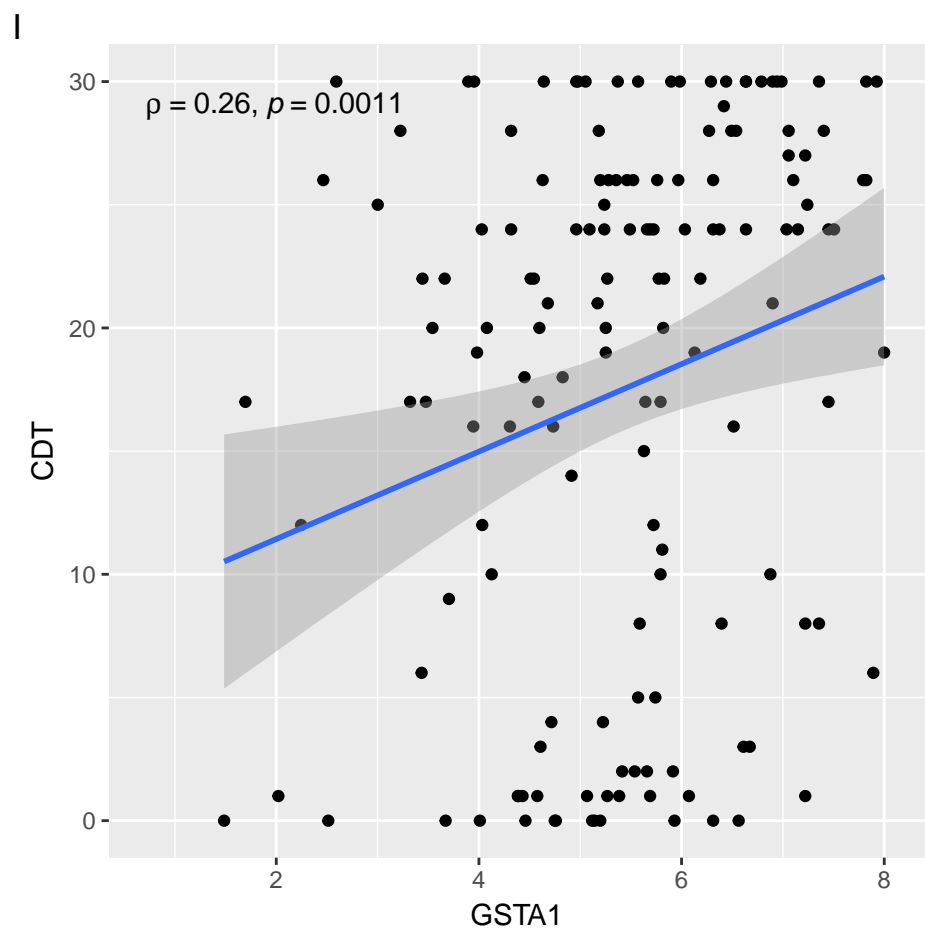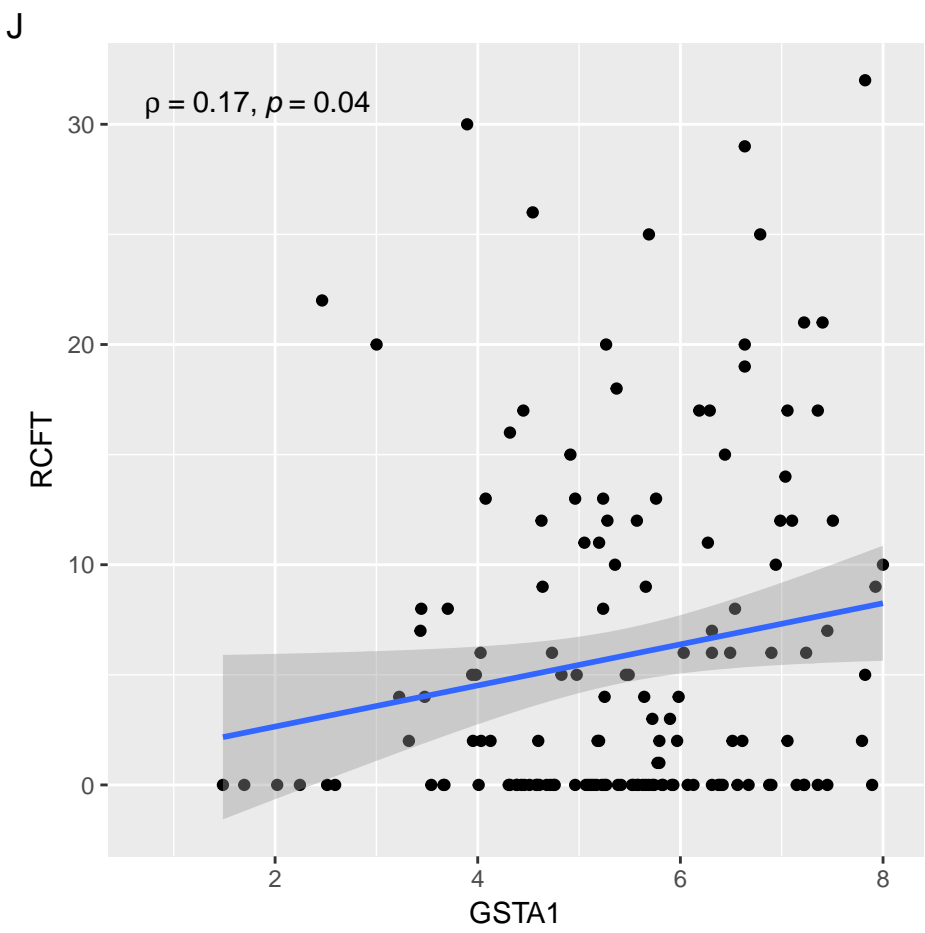

Supplement: Supplementary file 5 — Additional file 5: Fig. S5. Scatter plots of different diagnostic proteins with different cognition tests. [file 13195_2023_1324_MOESM5_ESM.zip › additional Fig 5-GSTA1.pdf]

A

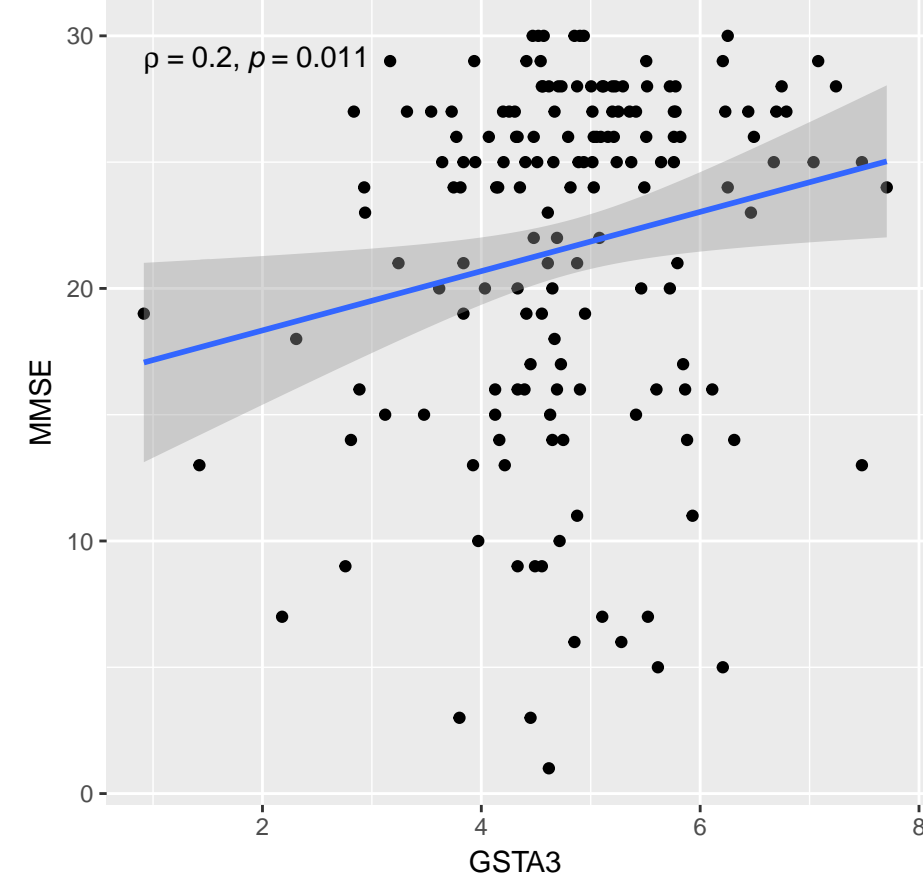

B

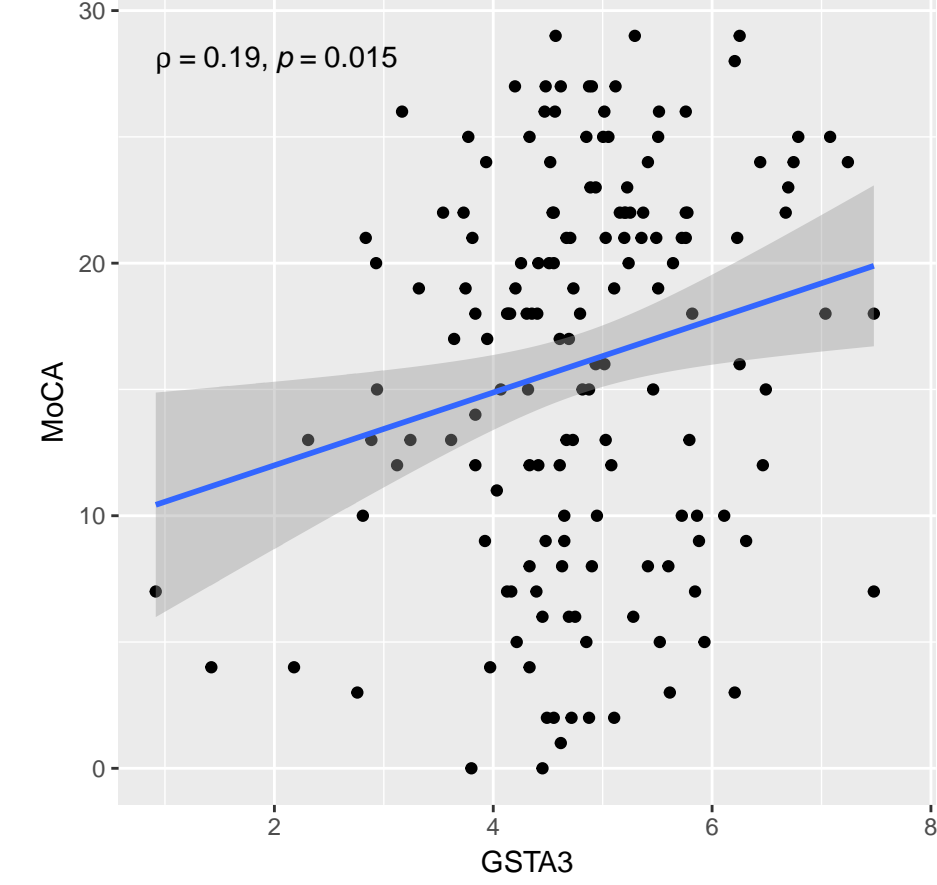

C

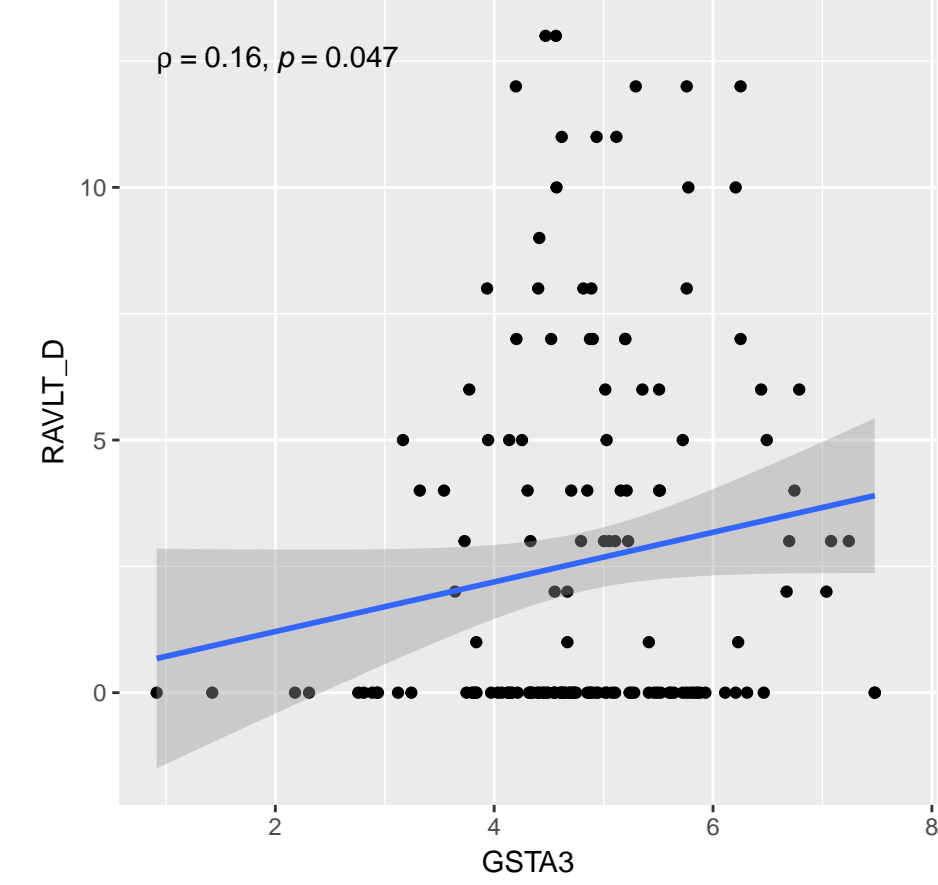

D

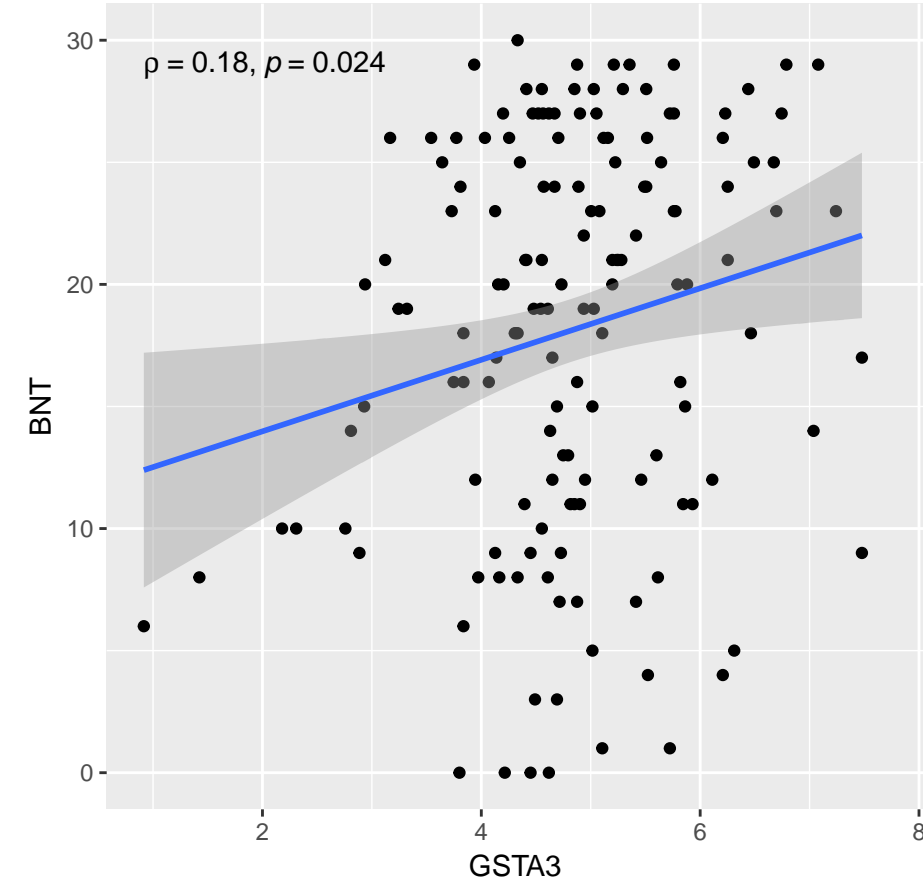

E

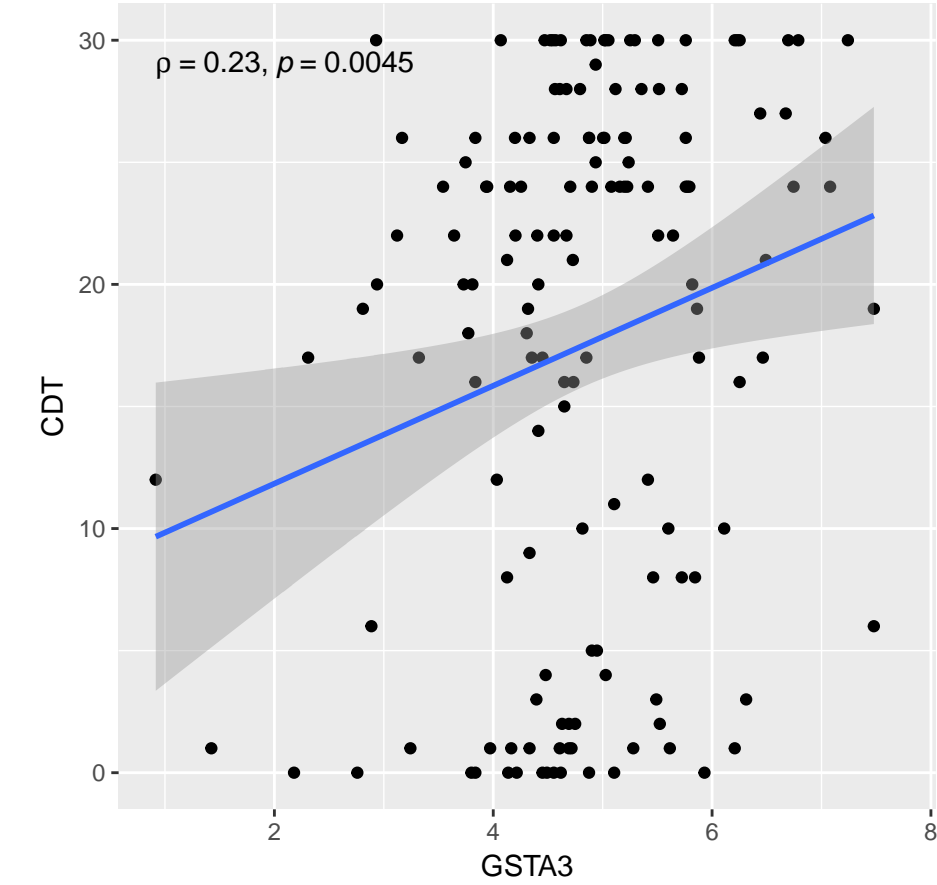

Supplement: Supplementary file 5 — Additional file 5: Fig. S5. Scatter plots of different diagnostic proteins with different cognition tests. [file 13195_2023_1324_MOESM5_ESM.zip › additional Fig 5-GSTA3.pdf]

A

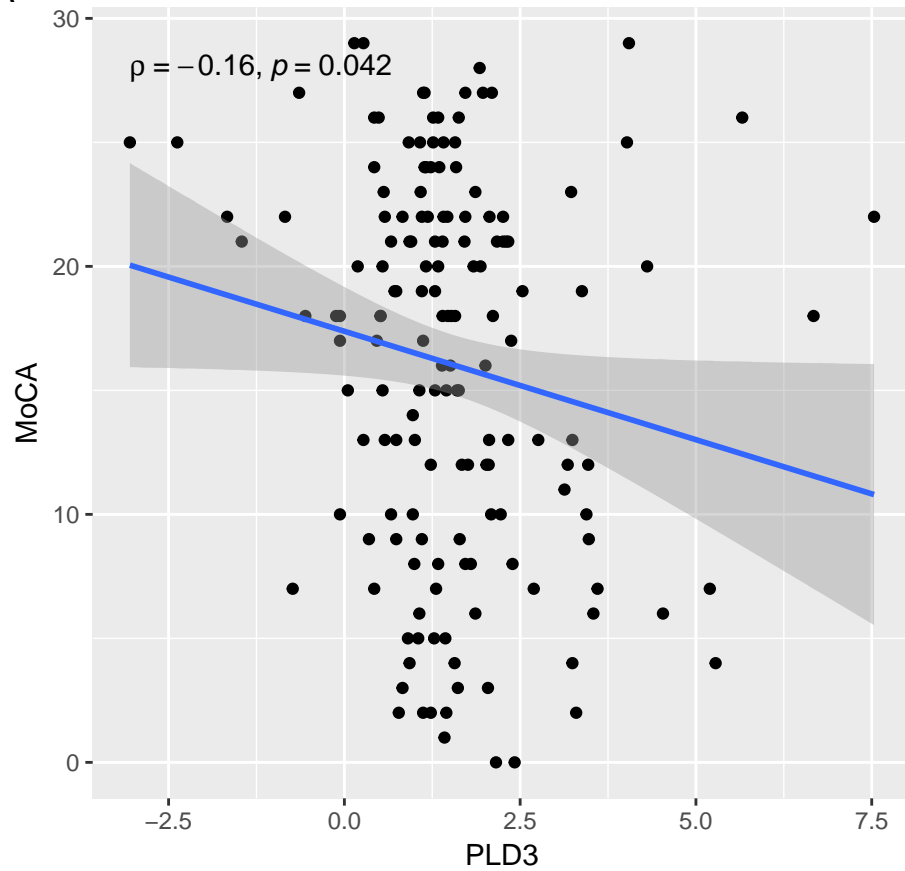

B

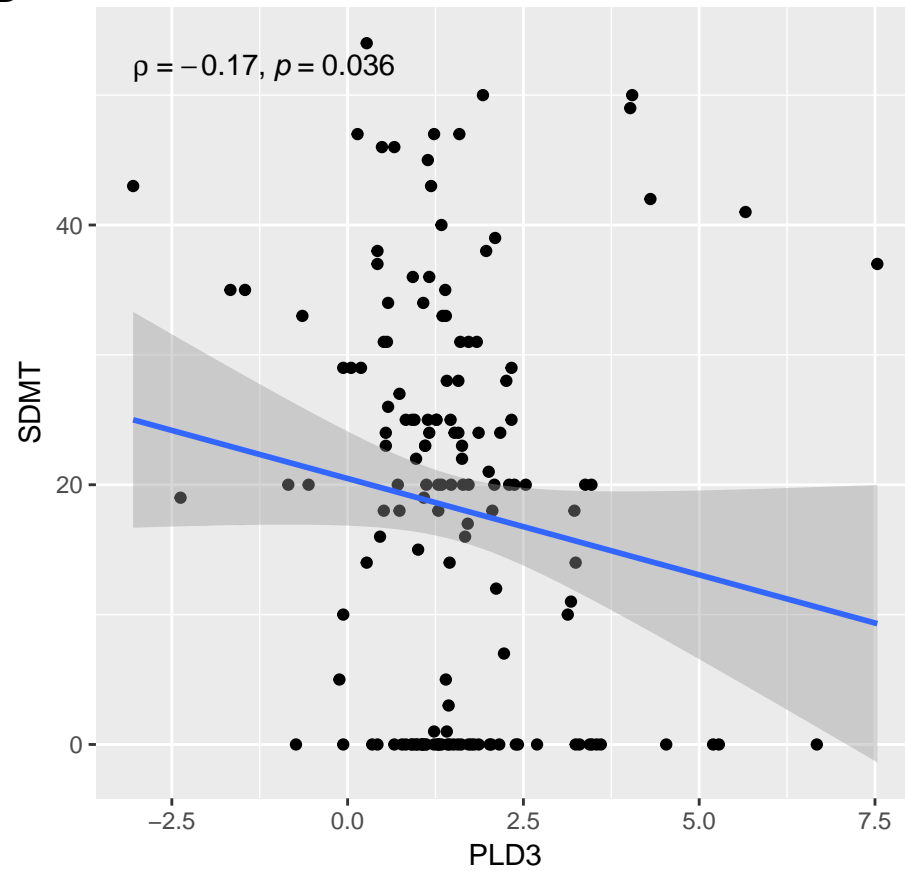

C

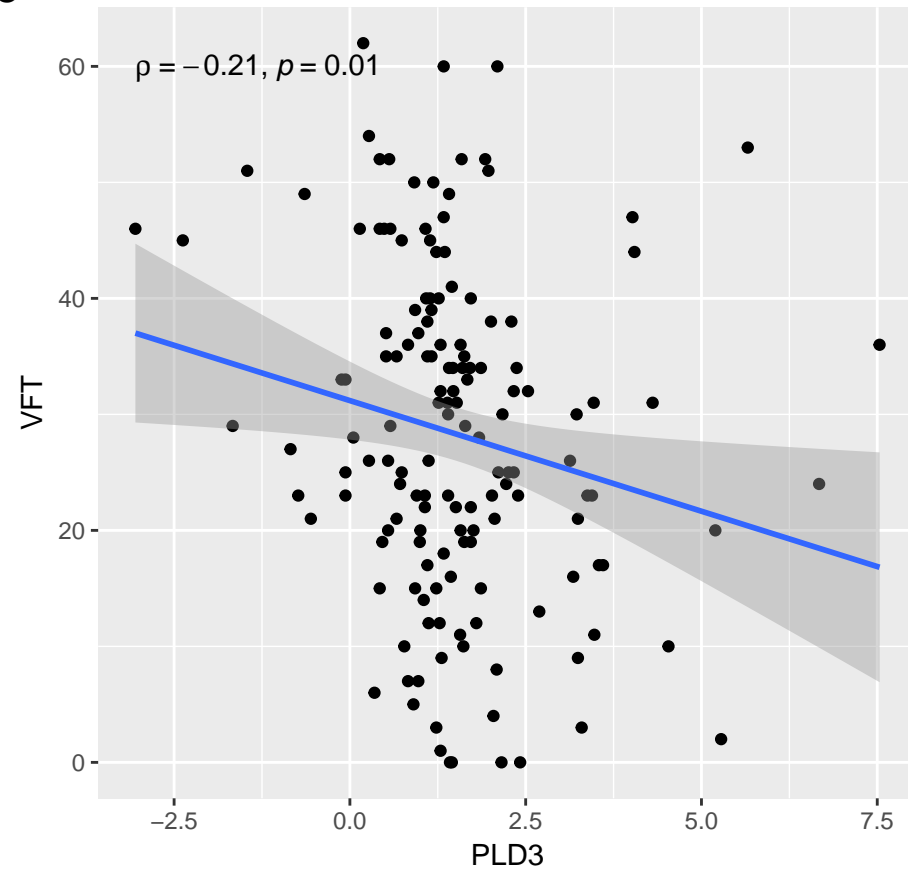

Supplement: Supplementary file 5 — Additional file 5: Fig. S5. Scatter plots of different diagnostic proteins with different cognition tests. [file 13195_2023_1324_MOESM5_ESM.zip › additional Fig 5-PLD3.pdf]

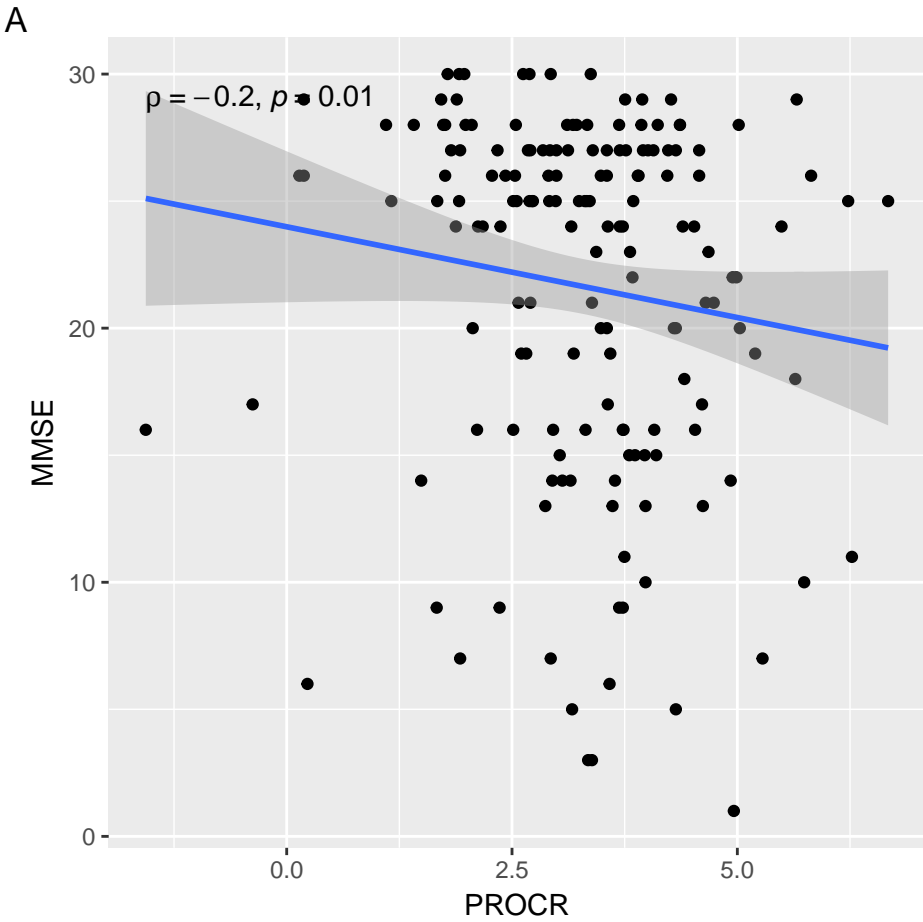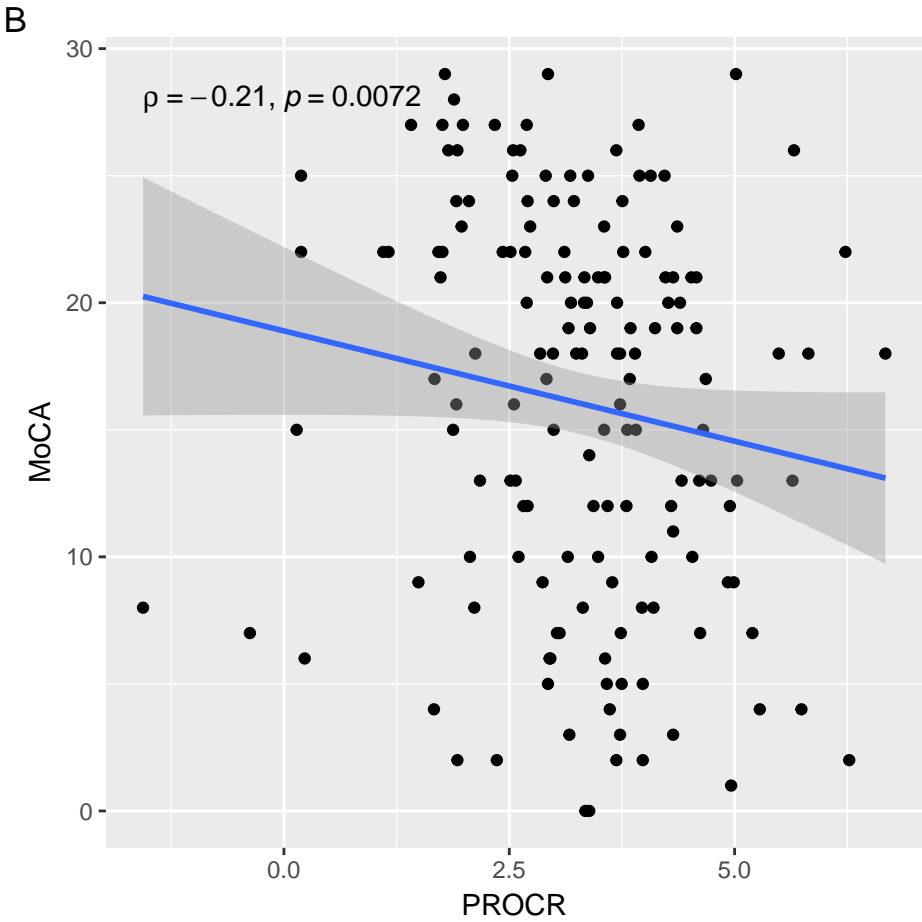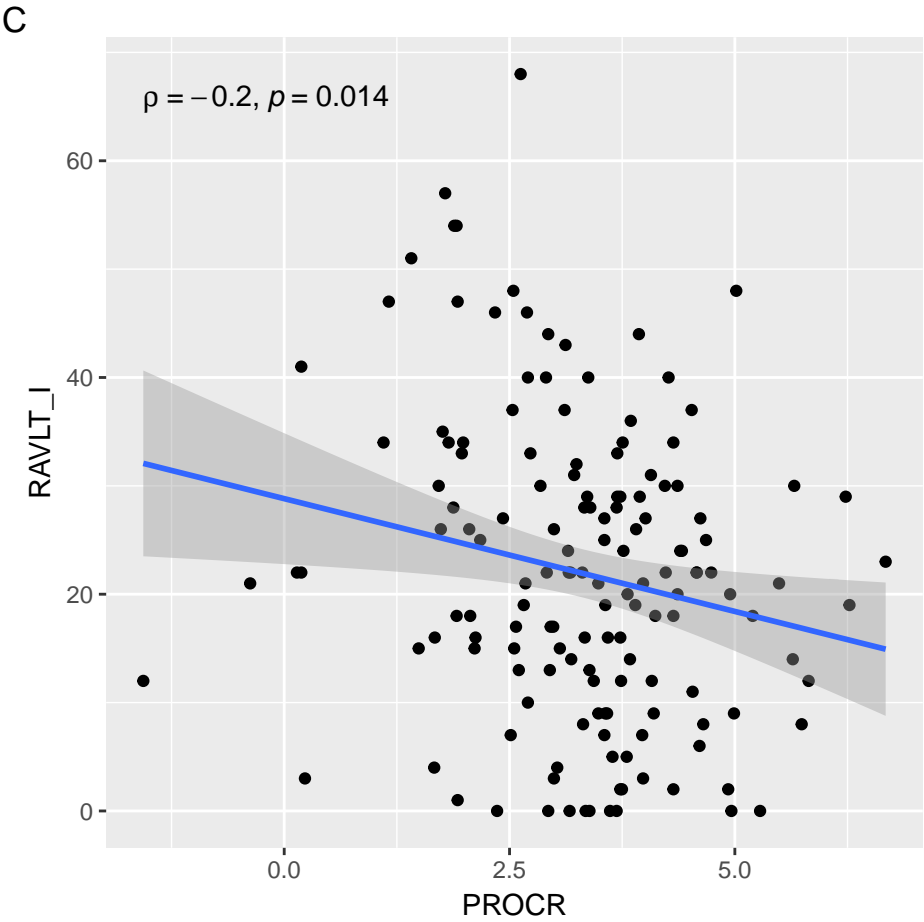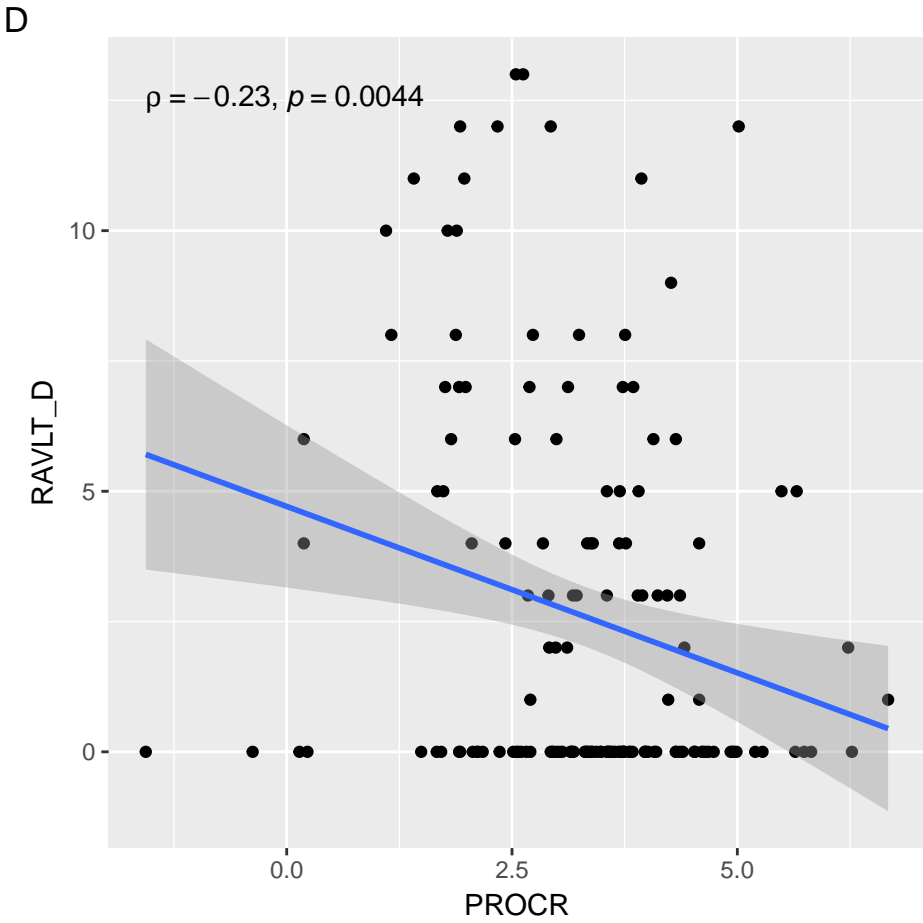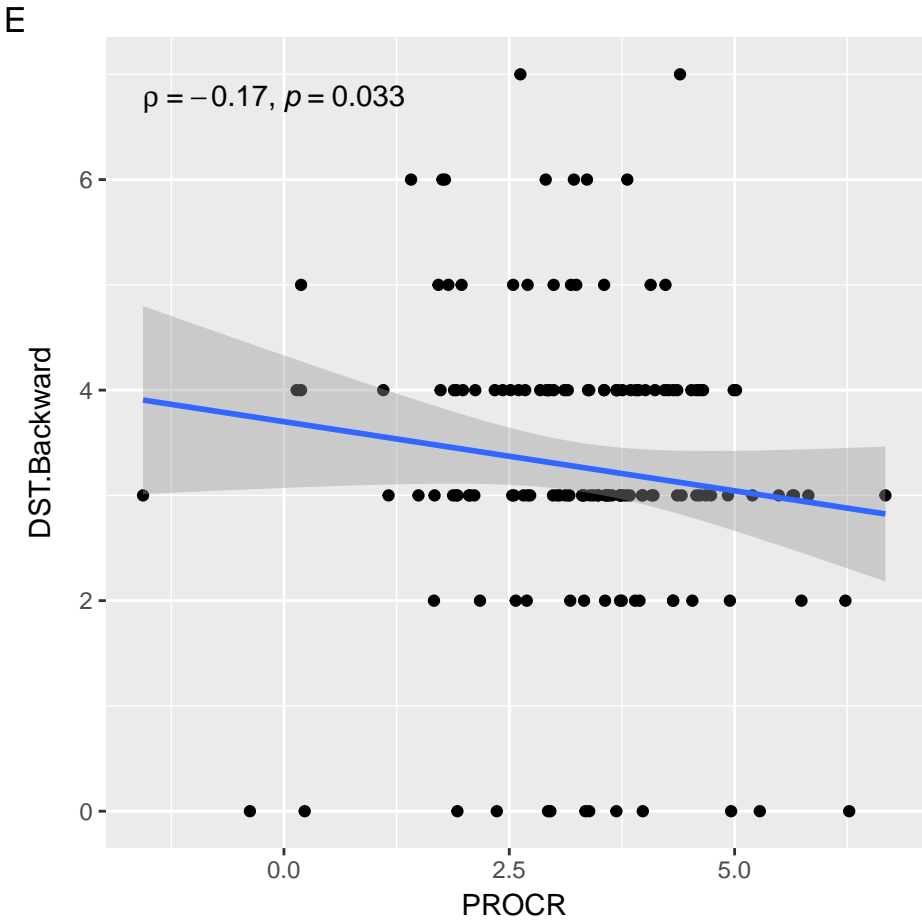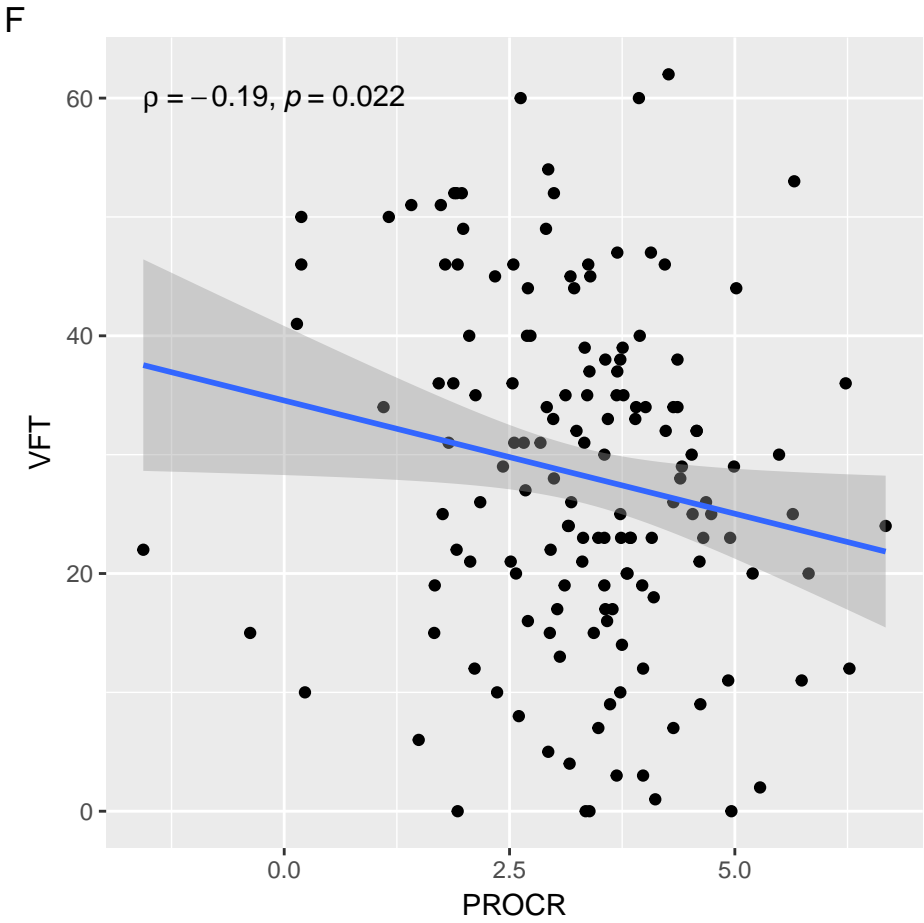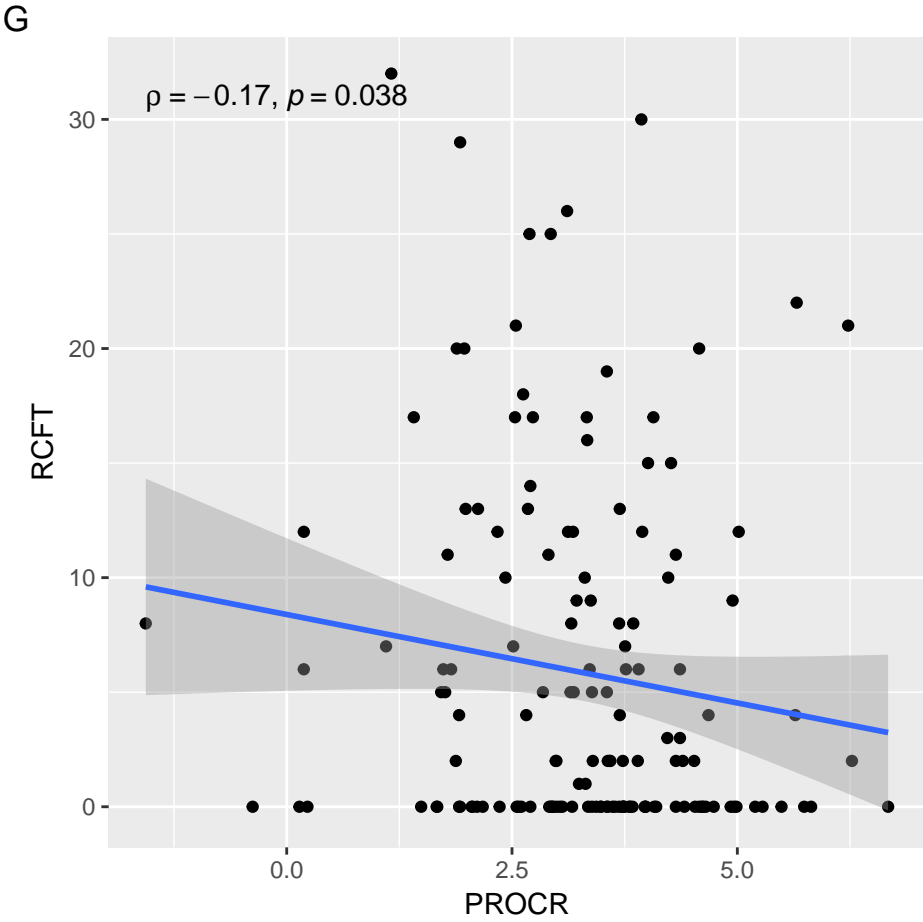

Supplement: Supplementary file 5 — Additional file 5: Fig. S5. Scatter plots of different diagnostic proteins with different cognition tests. [file 13195_2023_1324_MOESM5_ESM.zip › additional Fig 5-PROCR.pdf]

A

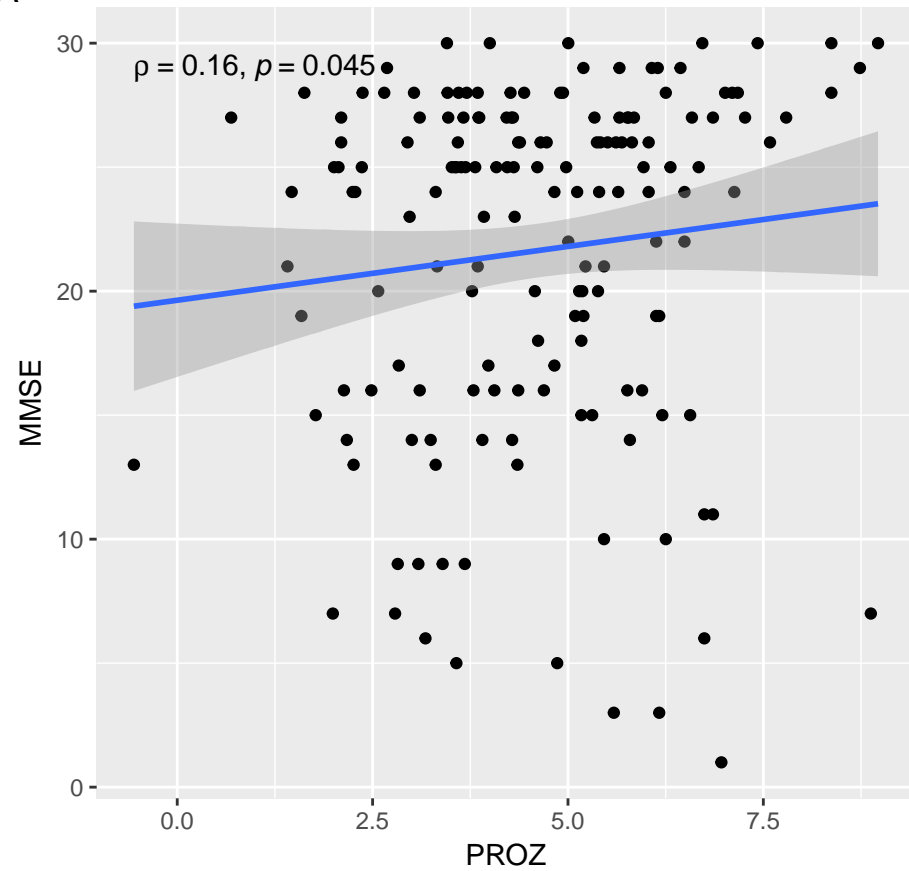

B

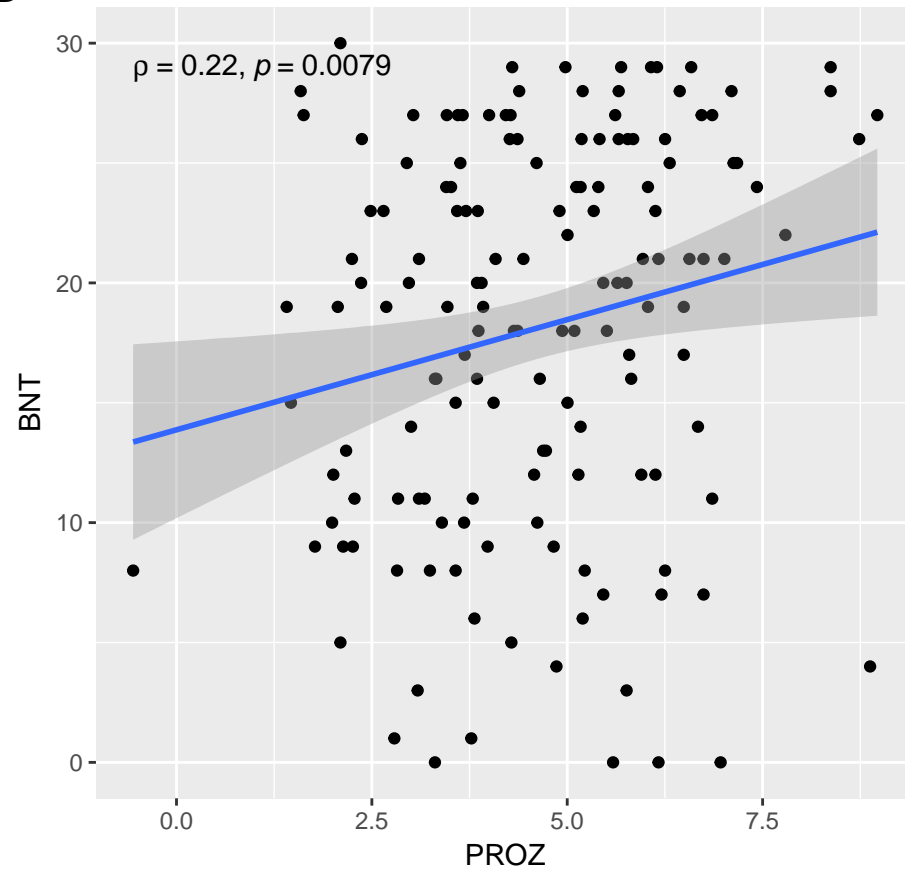

C

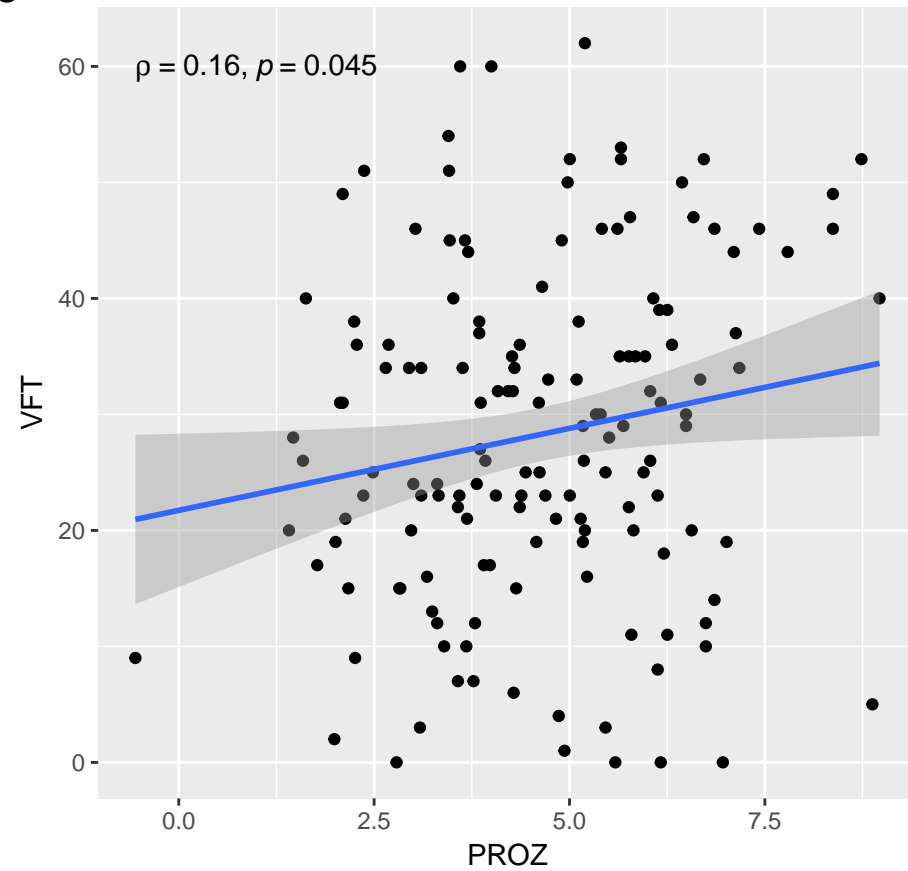

Supplement: Supplementary file 5 — Additional file 5: Fig. S5. Scatter plots of different diagnostic proteins with different cognition tests. [file 13195_2023_1324_MOESM5_ESM.zip › additional Fig 5-PROZ.pdf]

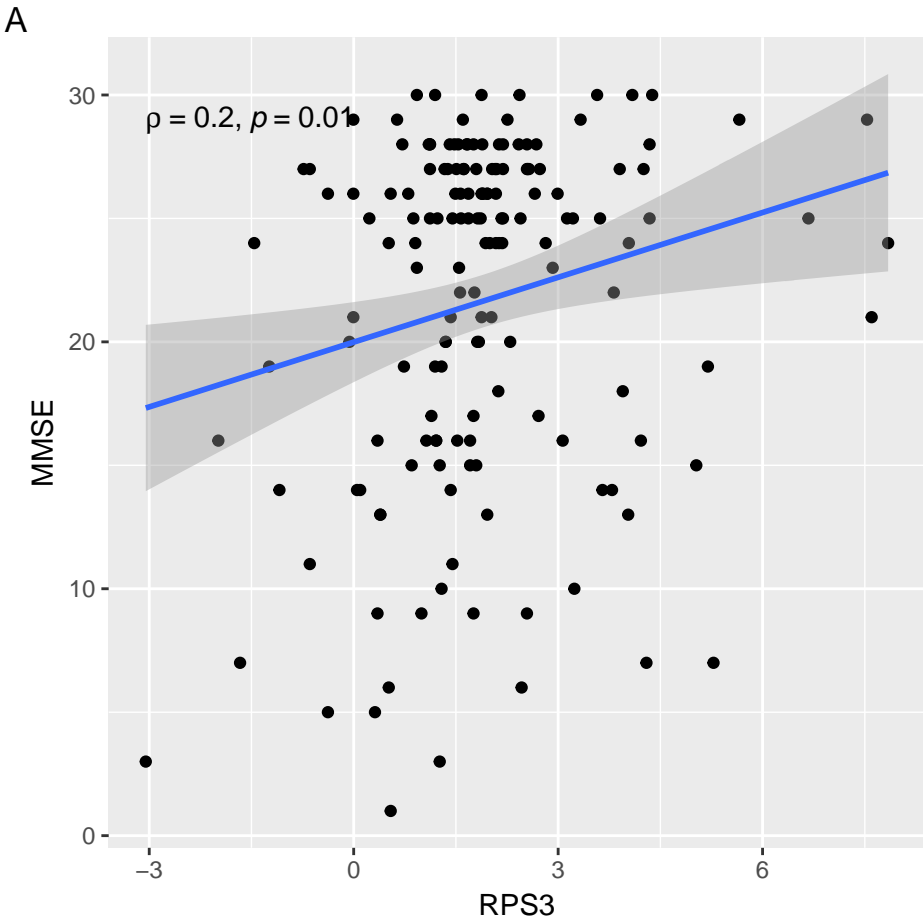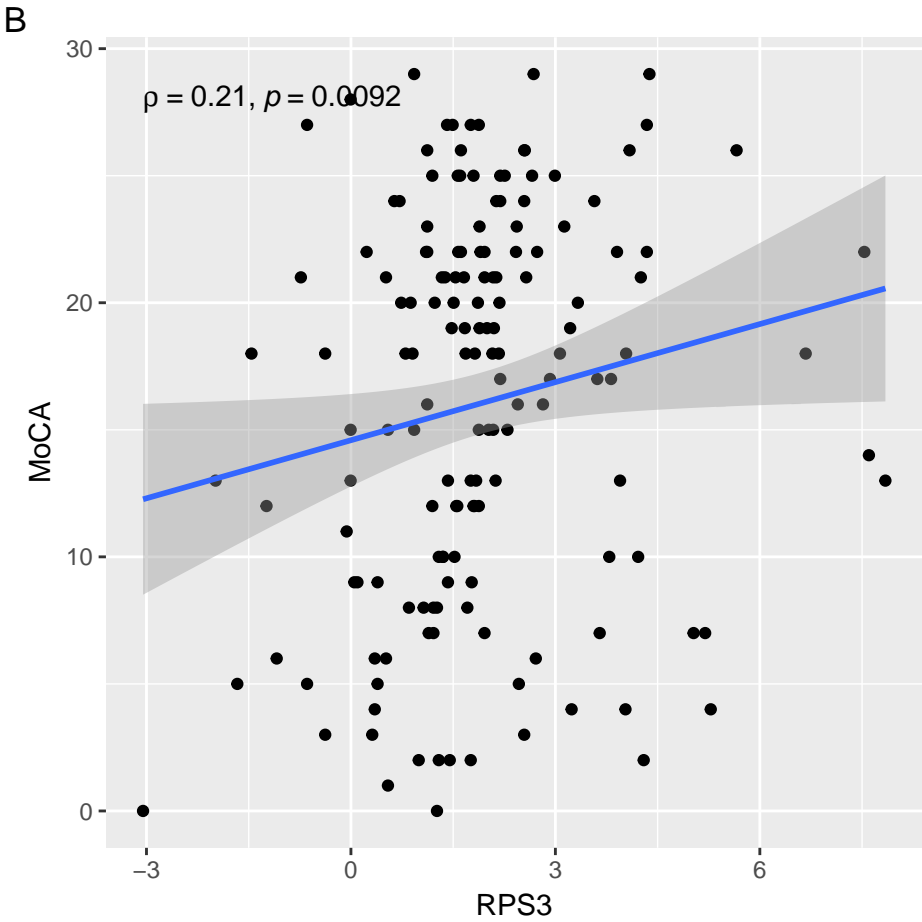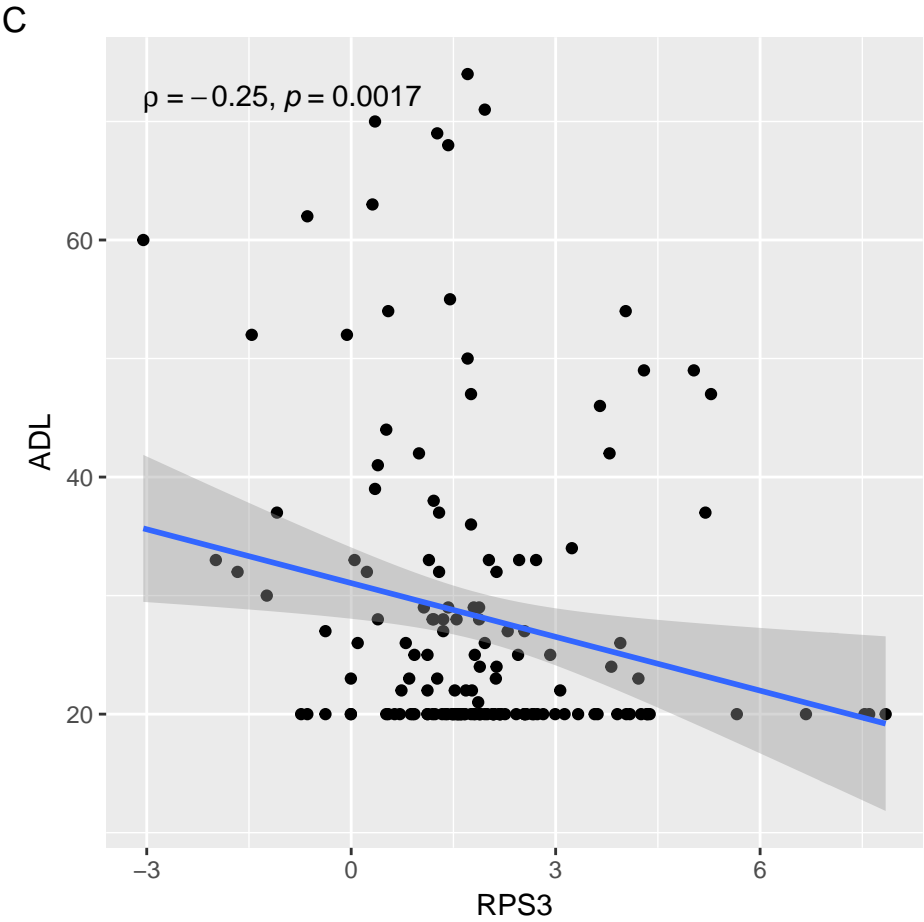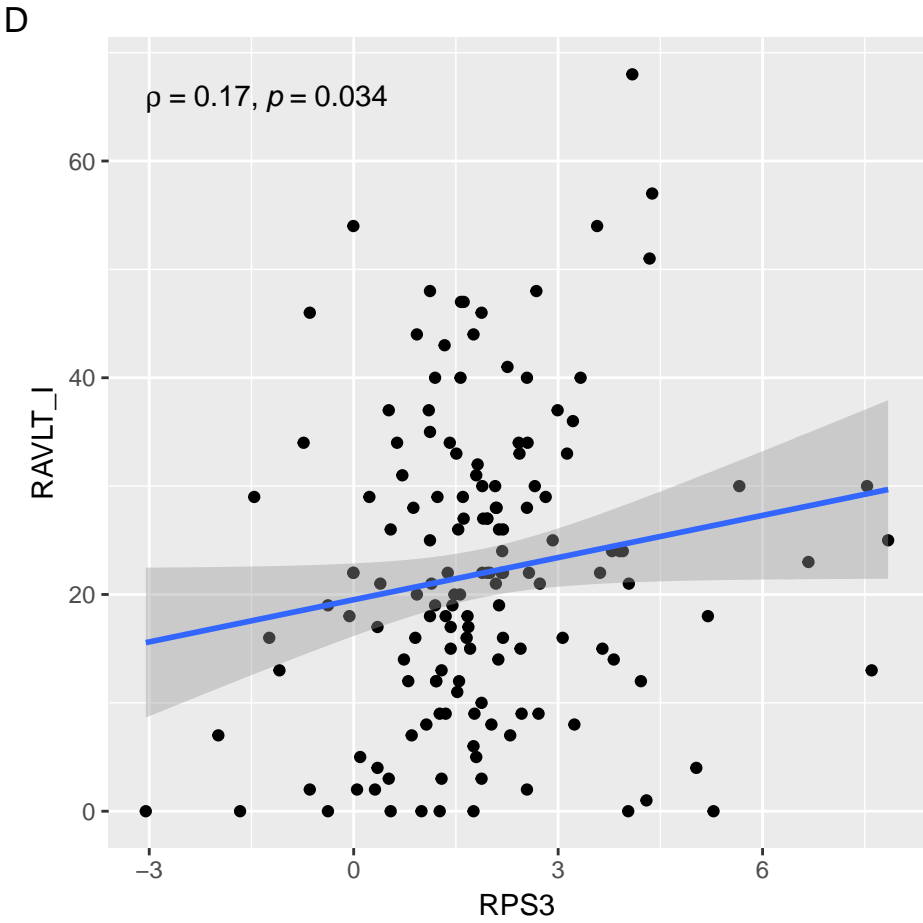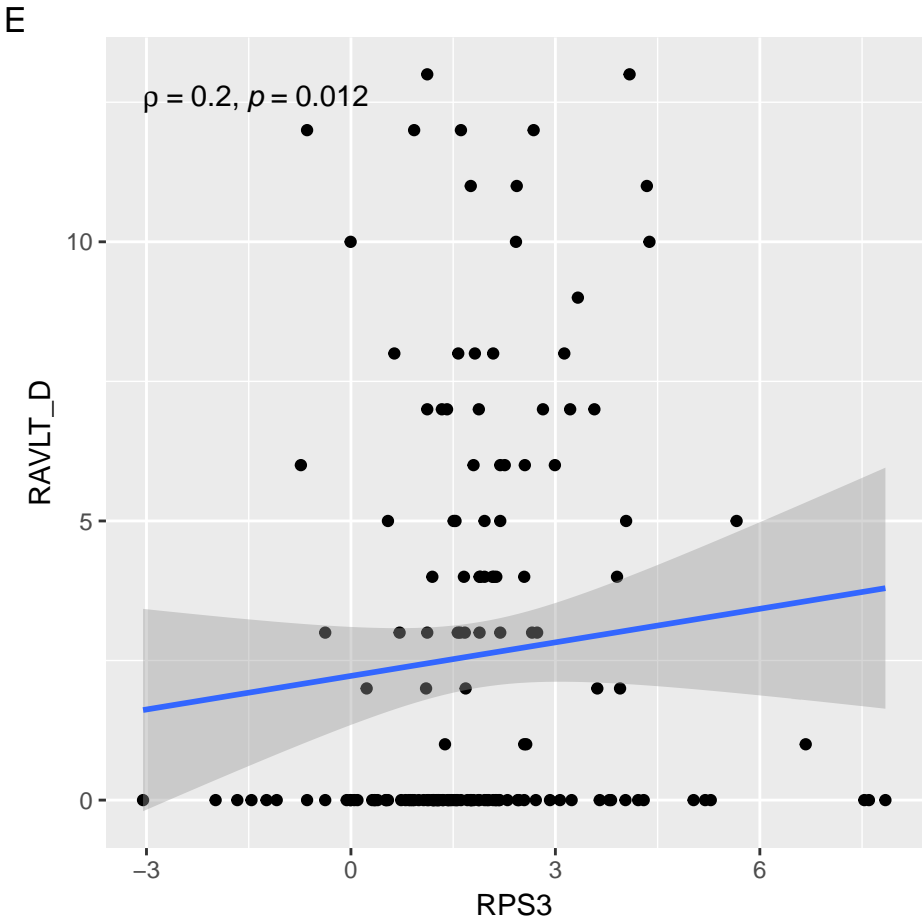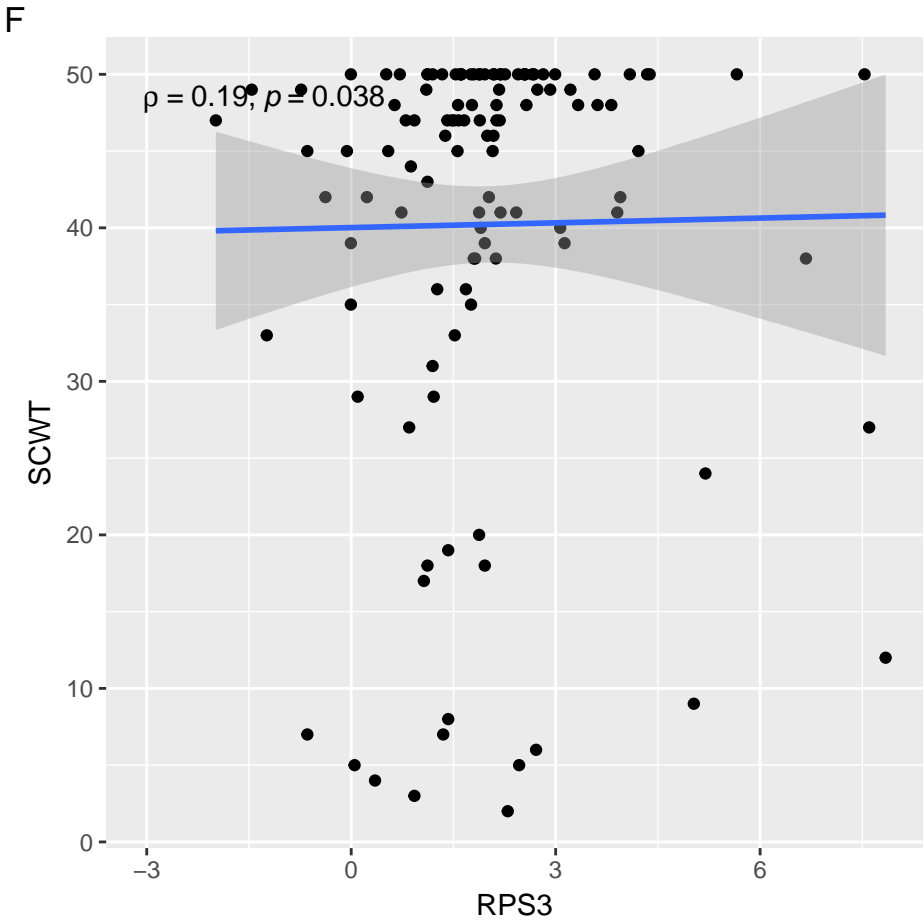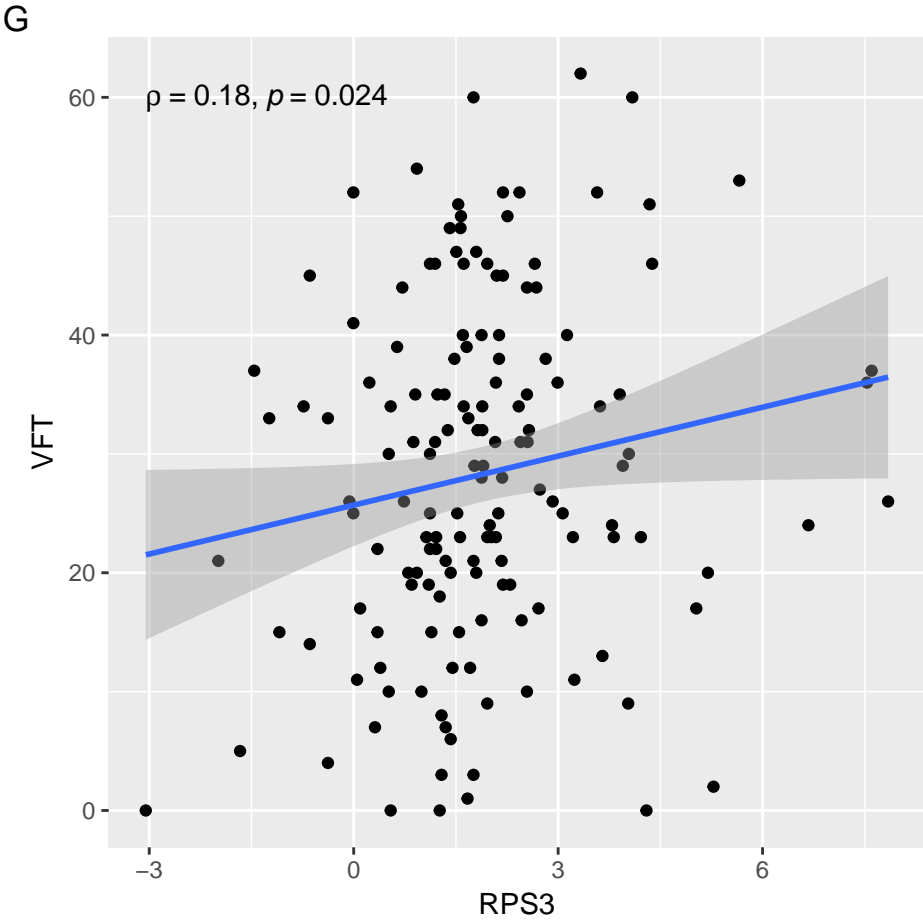

Supplement: Supplementary file 5 — Additional file 5: Fig. S5. Scatter plots of different diagnostic proteins with different cognition tests. [file 13195_2023_1324_MOESM5_ESM.zip › additional Fig 5-RPS3.pdf]

**A**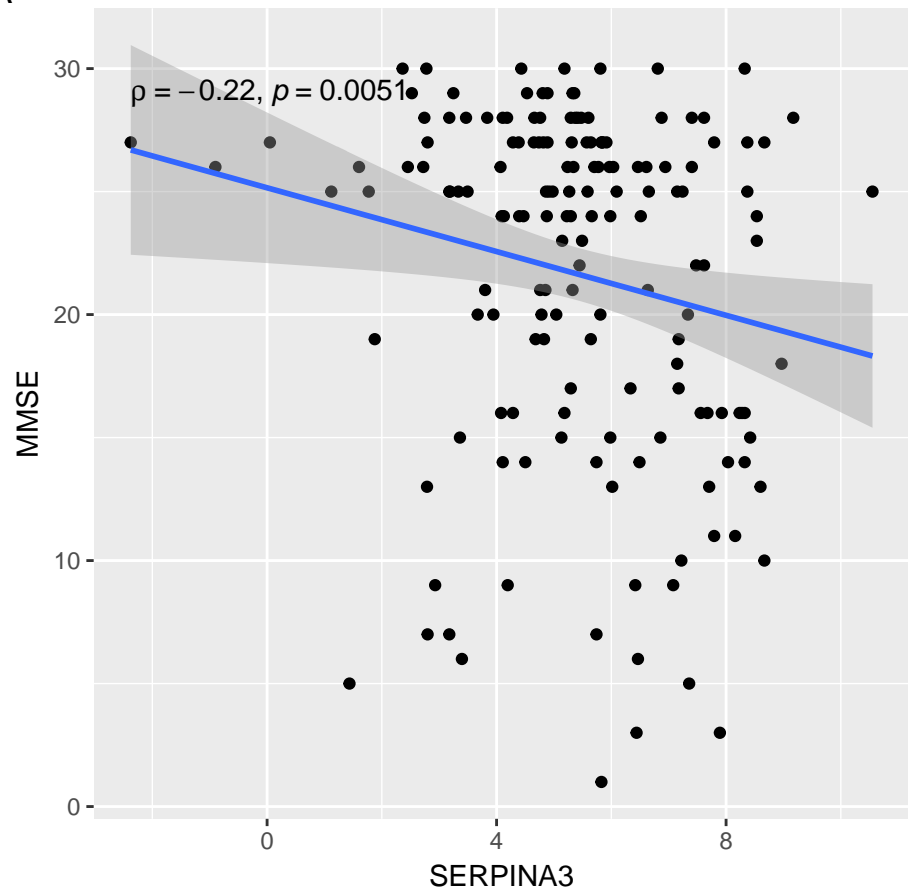**B**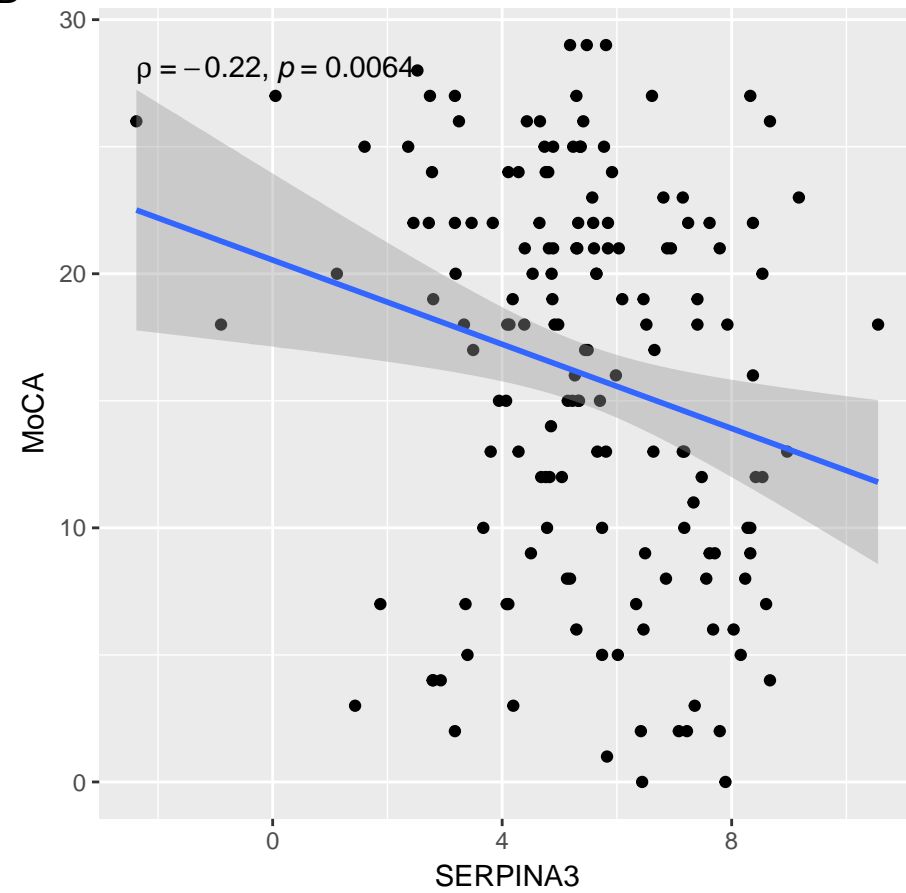**C**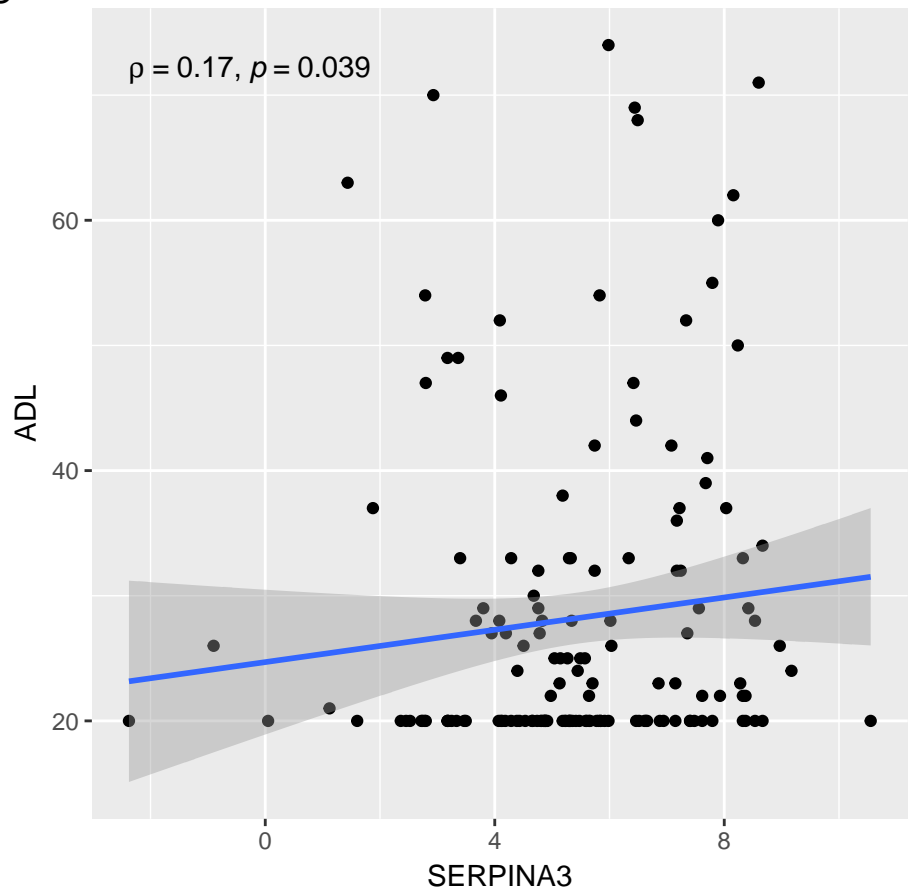**D**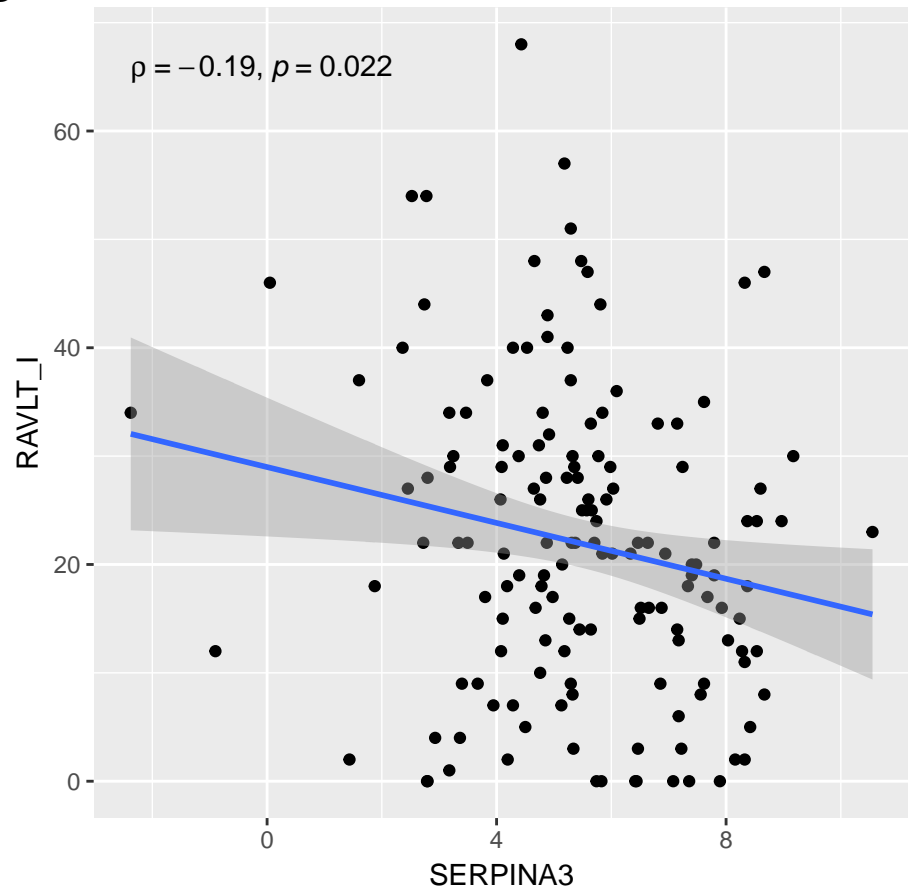**E**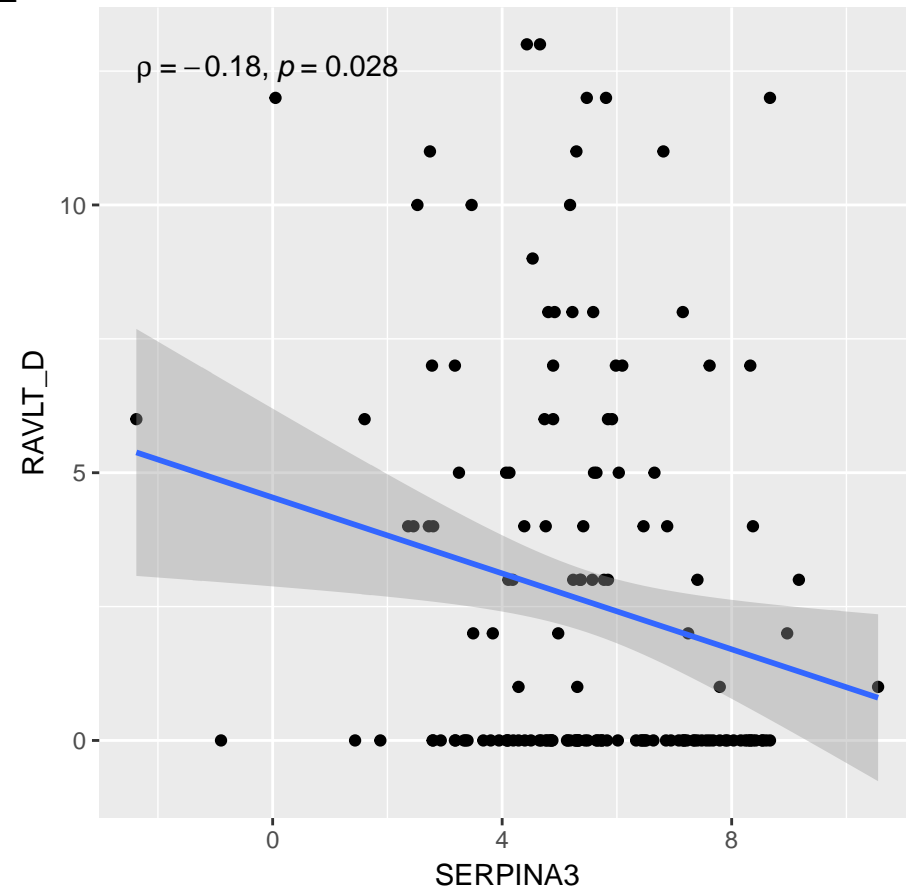**F**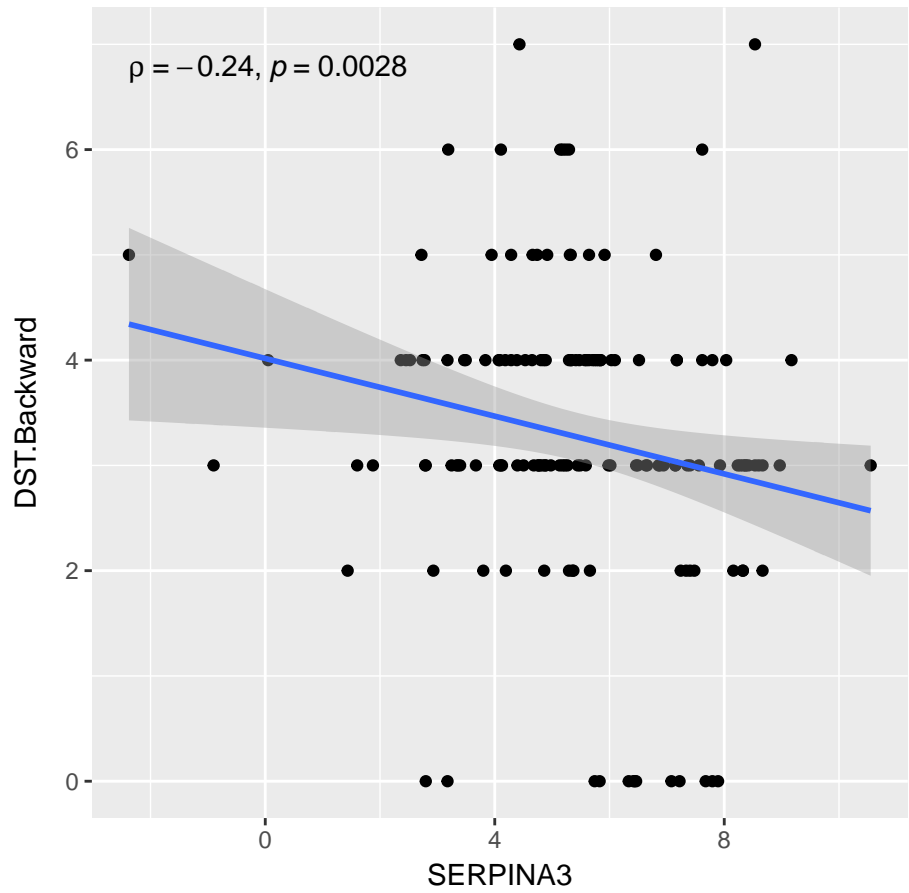**G**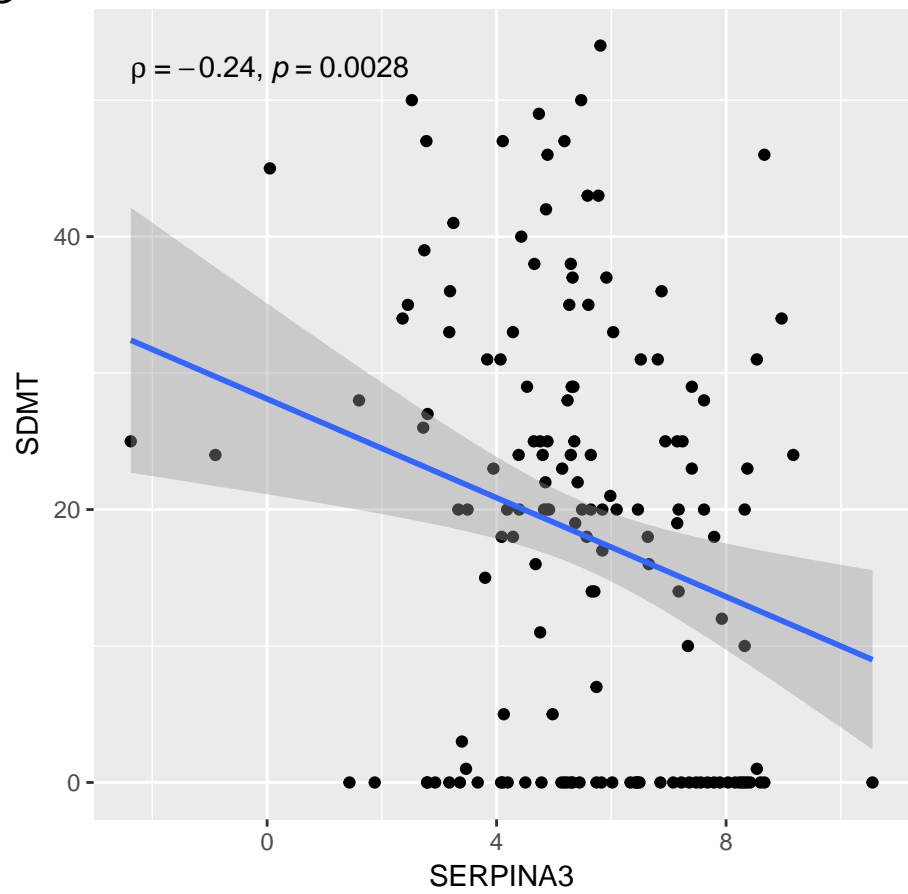**H**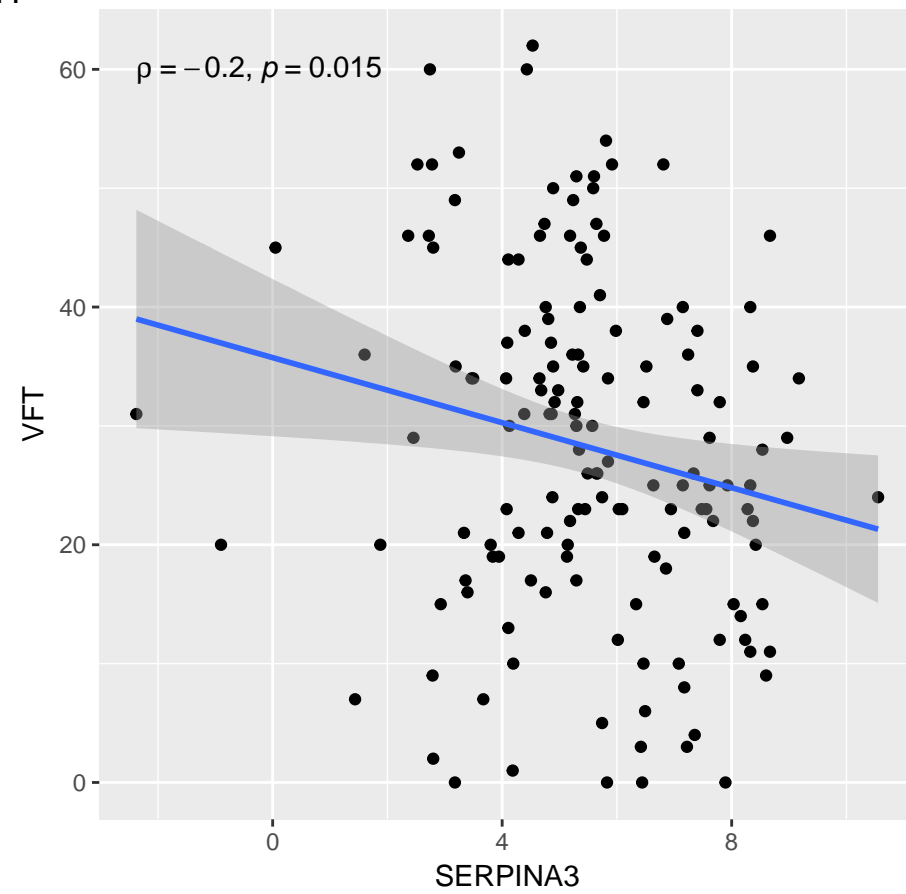**I**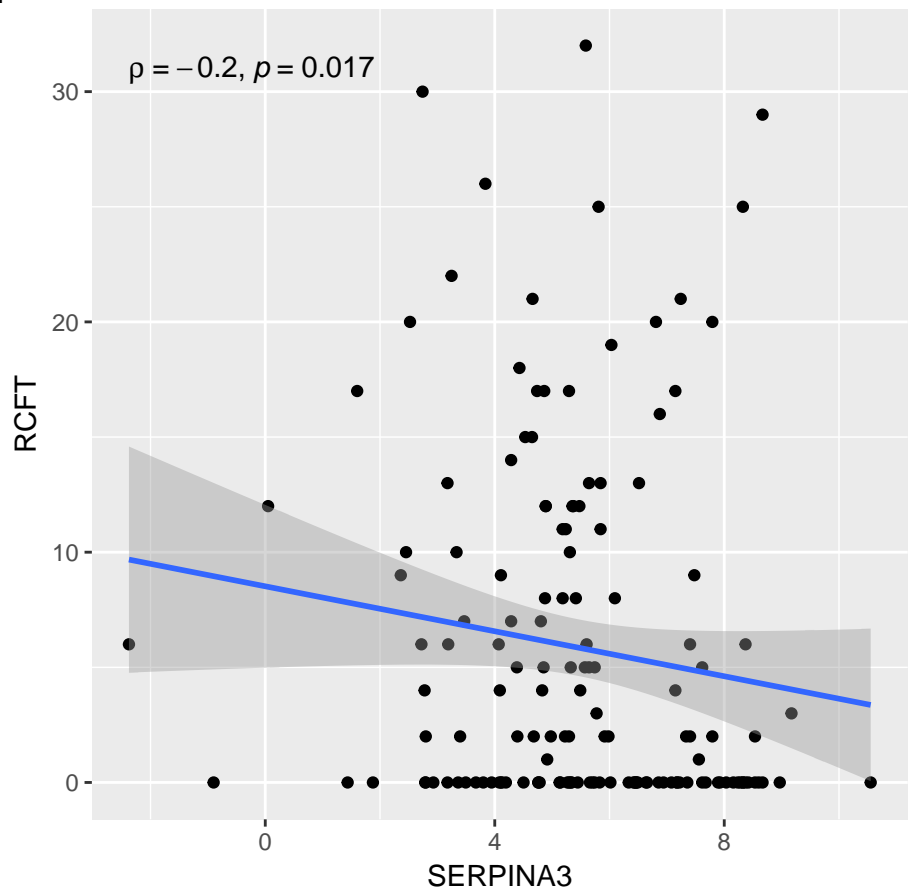

Supplement: Supplementary file 5 — Additional file 5: Fig. S5. Scatter plots of different diagnostic proteins with different cognition tests. [file 13195_2023_1324_MOESM5_ESM.zip › additional Fig 5-SERPINA3.pdf]

A

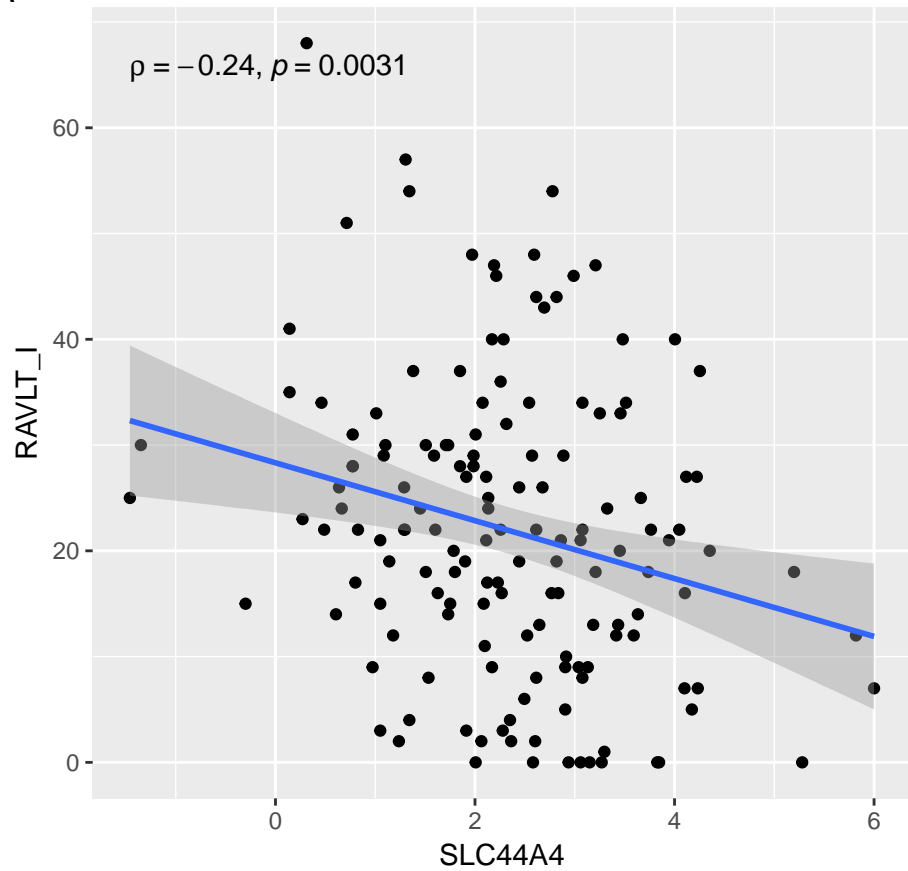

B

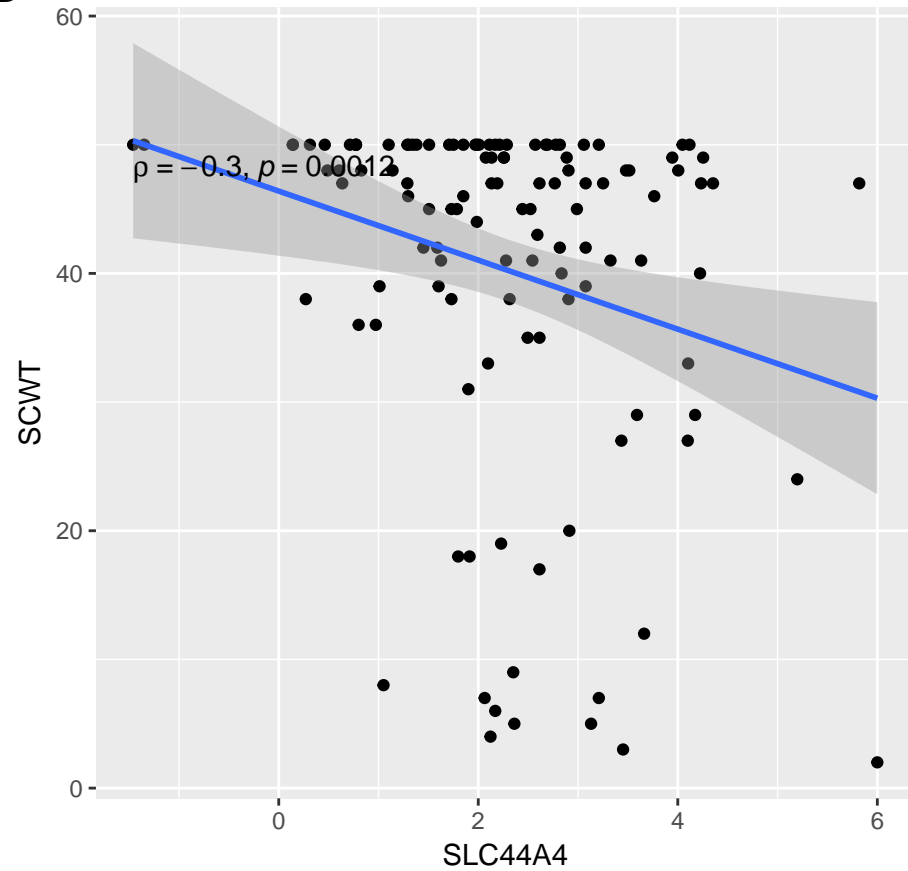

C

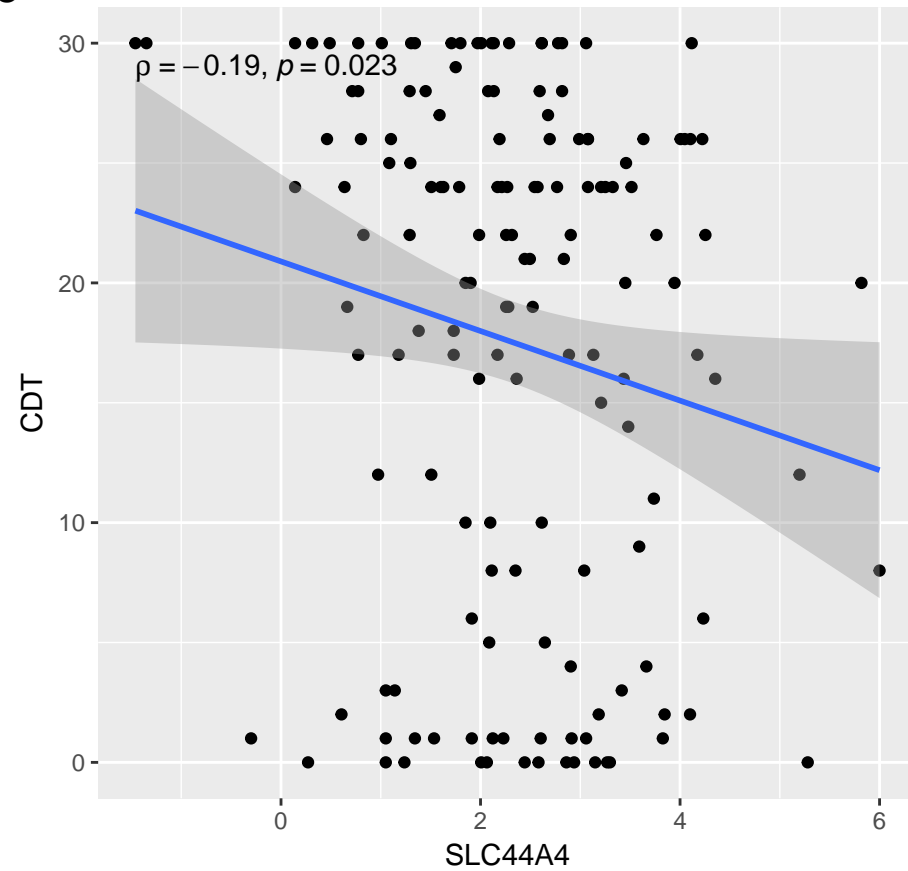

Supplement: Supplementary file 5 — Additional file 5: Fig. S5. Scatter plots of different diagnostic proteins with different cognition tests. [file 13195_2023_1324_MOESM5_ESM.zip › additional Fig 5-SLC44A4.pdf]

A

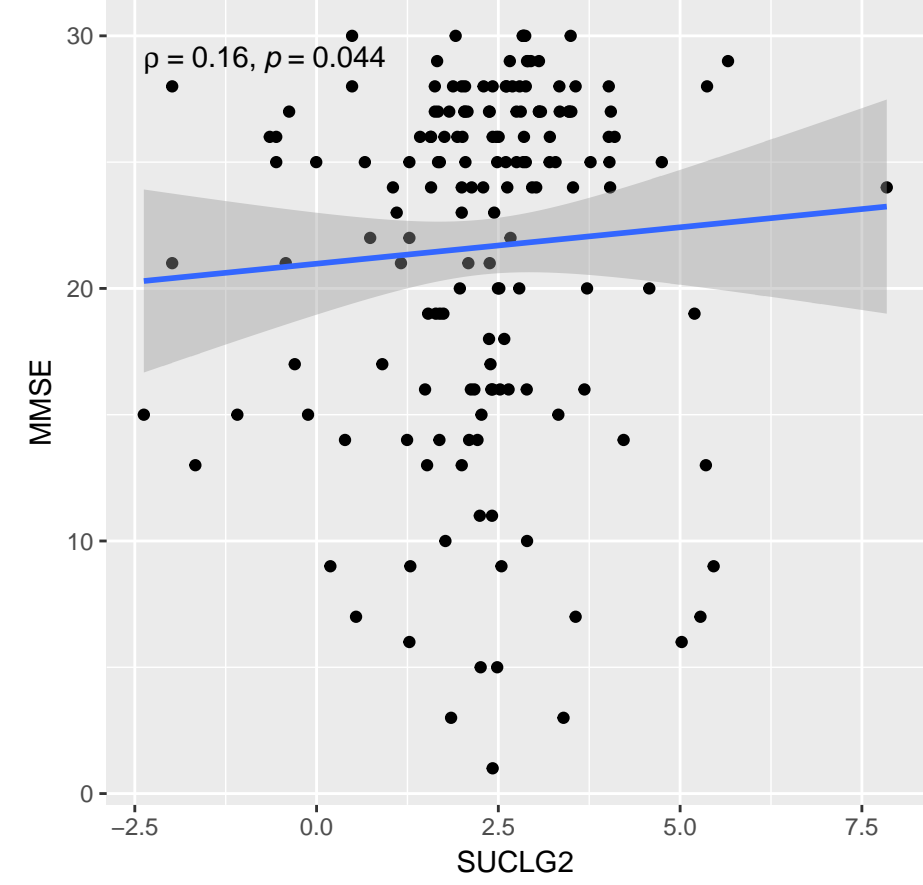

B

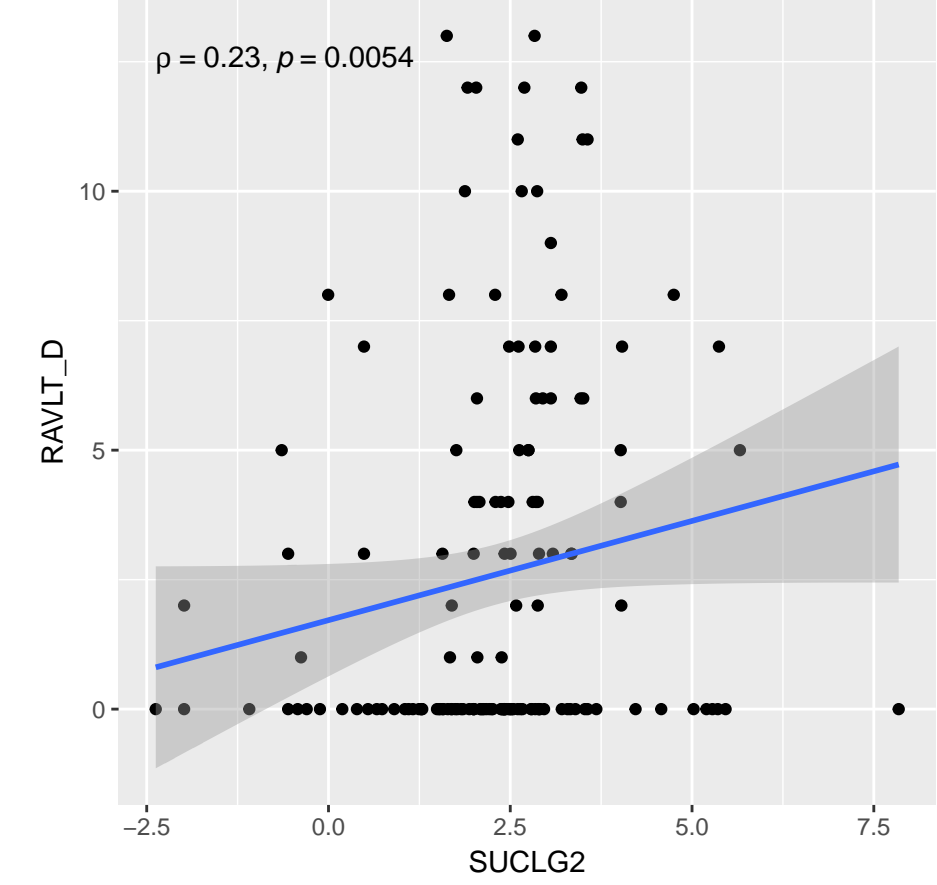

C

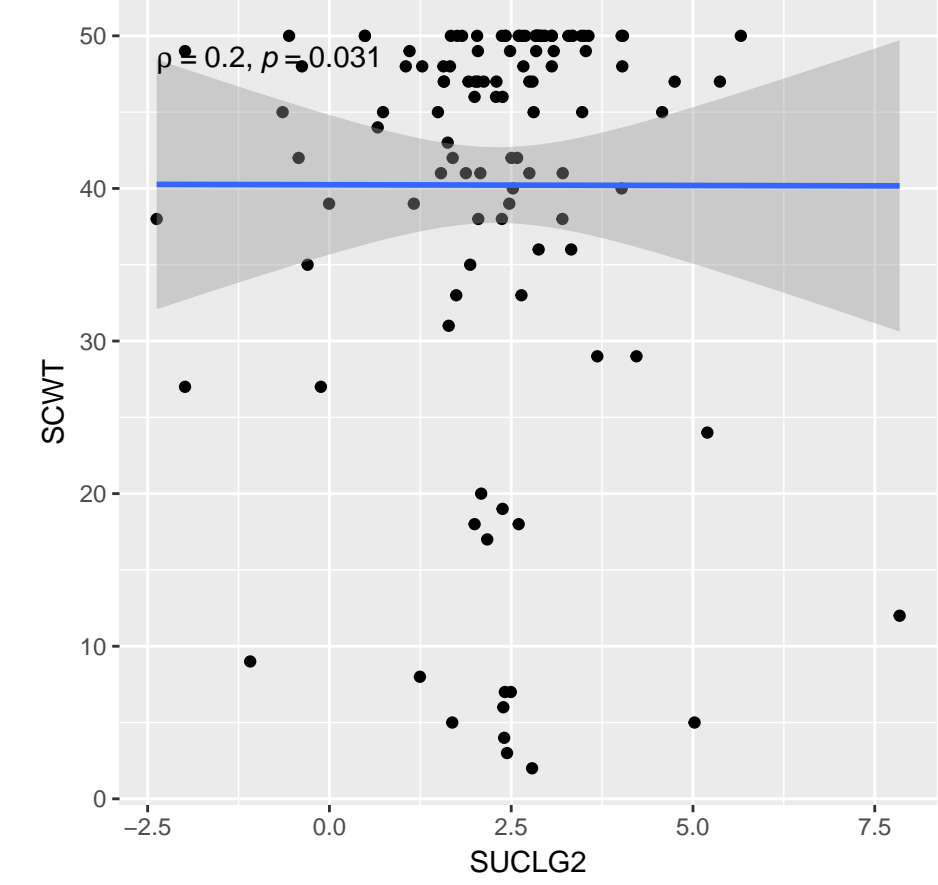

D

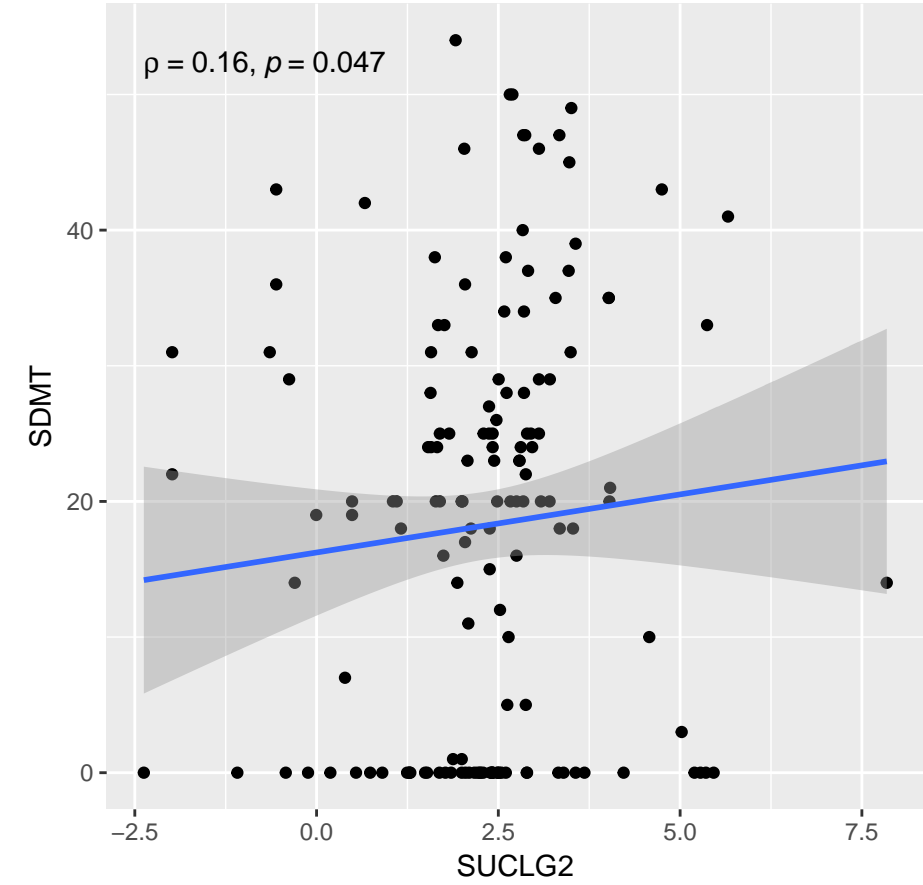

E

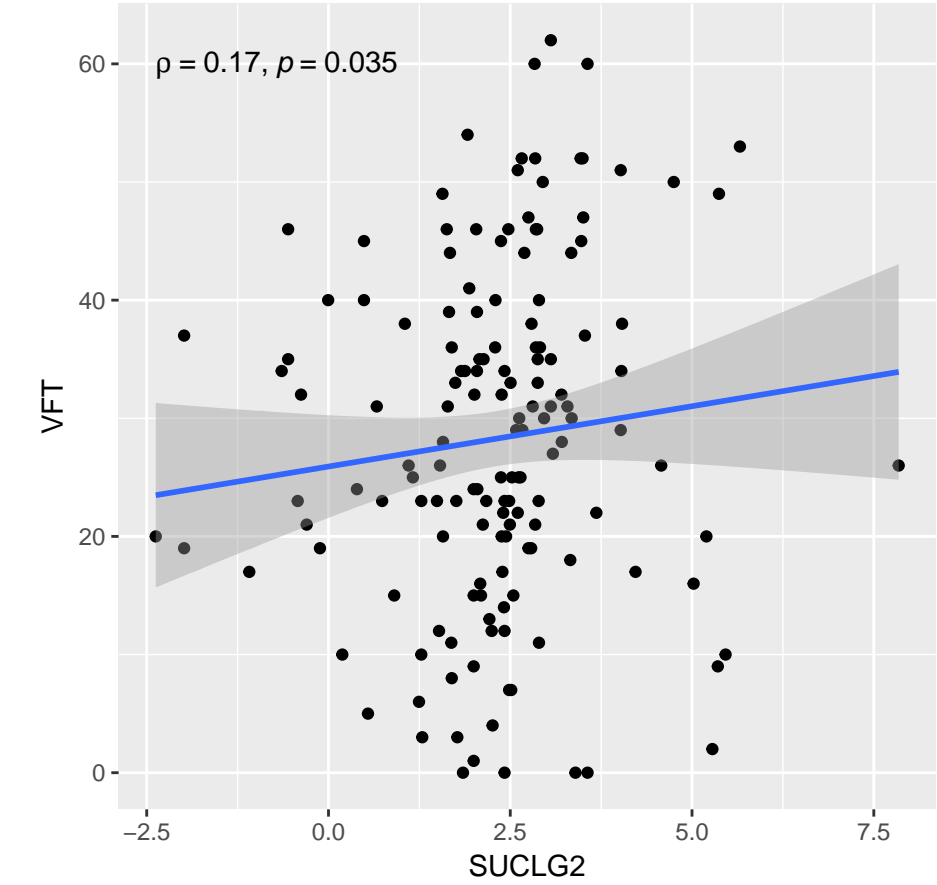

Supplement: Supplementary file 5 — Additional file 5: Fig. S5. Scatter plots of different diagnostic proteins with different cognition tests. [file 13195_2023_1324_MOESM5_ESM.zip › additional Fig 5-SUCLG2.pdf]

A

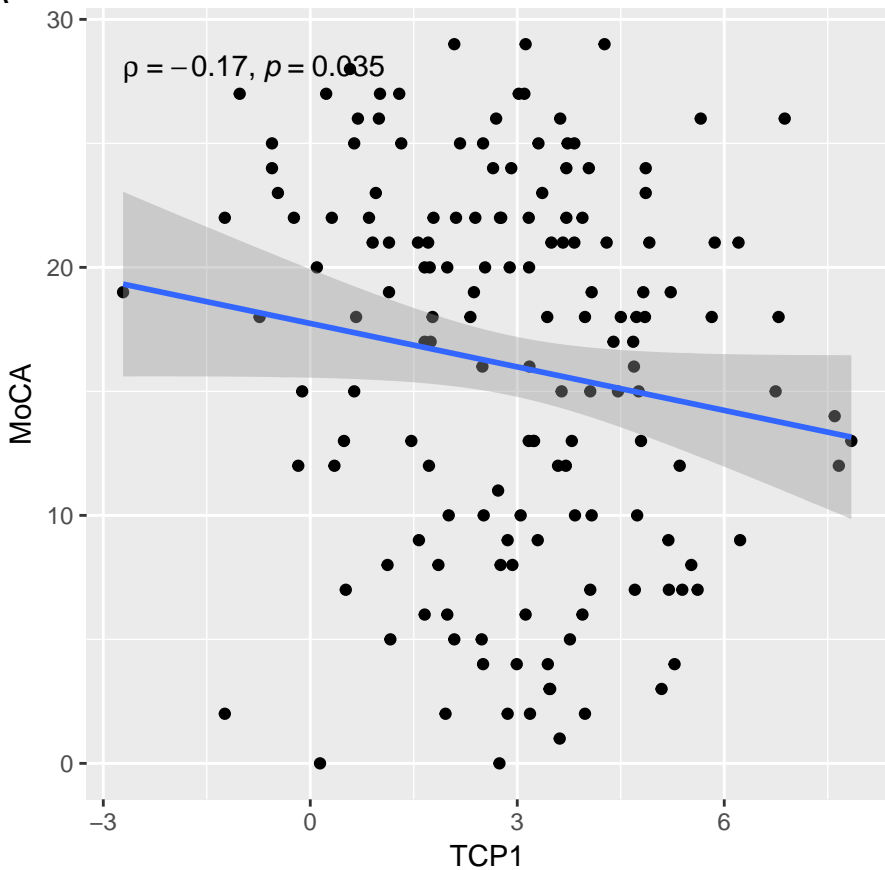

Supplement: Supplementary file 5 — Additional file 5: Fig. S5. Scatter plots of different diagnostic proteins with different cognition tests. [file 13195_2023_1324_MOESM5_ESM.zip › additional Fig 5-TCP1.pdf]
